# Library screening of cell-penetrating peptide for BY-2 cells, leaves of Arabidopsis, tobacco, tomato, poplar, and rice callus

**DOI:** 10.1038/s41598-018-29298-6

**Published:** 2018-07-20

**Authors:** Keiji Numata, Yoko Horii, Kazusato Oikawa, Yu Miyagi, Taku Demura, Misato Ohtani

**Affiliations:** 1 Biomacromolecules Research Team, RIKEN Center for Sustainable Resource Science, 2-1 Hirosawa, Wako-shi, Saitama 351-0198 Japan; 2 Graduate School of Biological Sciences, Nara Institute of Science and Technology, Ikoma, Nara 630-0192 Japan

## Abstract

Cell-penetrating peptides (CPPs) are used for various applications, especially in the biomedical field. Recently, CPPs have been used as a part of carrier to deliver proteins and/or genes into plant cells and tissues; hence, these peptides are attractive tools for plant biotechnological and agricultural applications, but require more efficient delivery rates and optimization by species before wide-scale use can be achieved. Here, we developed a library containing 55 CPPs to determine the optimal CPP characteristics for penetration of BY-2 cells and leaves of *Nicotiana benthamiana*, *Arabidopsis thaliana*, tomato (*Solanum lycopersicum*), poplar (hybrid aspen *Populus tremula* × *tremuloides* line T89), and rice (*Oryza sativa*). By investigating the cell penetration efficiency of CPPs in the library, we identified several efficient CPPs for all the plants studied except rice leaf. In the case of rice, several CPPs showed efficient penetration into rice callus. Furthermore, we examined the relationship between cell penetration efficiency and CPP secondary structural characteristics. The cell penetration efficiency of Lys-containing CPPs was relatively greater in plant than in animal cells, which could be due to differences in lipid composition and surface charge of the cell membranes. The variation in optimal CPPs across the plants studied here suggests that CPPs must be optimized for each plant species and target tissues of interest.

## Introduction

Cell-penetrating peptides (CPPs) are short peptides that penetrate cellular lipid bilayers or destabilize cellular membranes. Based on their chemical structures, CPPs can be classified roughly into three groups: cationic, amphipathic, and hydrophobic^1–3^. Cationic CPPs contain numerous positively charged amino acids, such as lysine (Lys) and arginine (Arg). Amphipathic CPPs are typically composed of an alternating sequence of polar and non-polar amino acids. Hydrophobic CPPs consist of non-polar amino acids with relatively low net charges. The structural and chemical differences among the three groups affect the cellular uptake pathways of the CPPs, which are either energy-independent or energy-dependent^4,5^. The energy-independent mechanism of uptake involves direct translocation across the cell membrane, whereas the energy-dependent mechanisms involve caveolae-dependent endocytosis, clathrin-dependent endocytosis, macropinocytosis, or phagocytosis^6–10^. Thus, although the chemical and physical properties, including the molecular structure, of CPPs affect their cellular uptake mechanism and efficiency, the specific amino acid sequences and structures that induce internalization into plant cells have not been clarified.

CPPs have been used as a part of carriers to deliver several types of molecules, such as DNA, RNA and proteins, into plant cells and tissues. However, CPP-mediated gene/protein delivery is not yet efficient enough for practical applications in plant breeding and modification. CPPs were used to deliver plasmid DNA (pDNA) into intact roots of *Vigna radiata* L. and *Glycine max* L^11^. and permeabilized embryos of *Triticum aestivum* cv^12^. Double-stranded RNA (dsRNA) was delivered into tobacco cells in suspension using CPPs to induce post-transcriptional gene silencing^13^. Unlike the *Agrobacterium*-mediated system, which can only be used for transformation in a limited number of species, the CPP-based system could potentially be used in any plant species. Cationic peptides, which bind to nucleic acids via electrostatic interactions, protect DNA from nuclease degradation and mediate gene transfer across cell and organelle membranes^14,15^. Using specific peptides, genes and proteins can be precisely targeted to intracellular organelles, which is not possible using either biolistic or *Agrobacterium*-mediated transformation^14,16,17^. Thus, this technology can be used to deliver proteins to specific intracellular targets or to express or silence genes of interest using DNA or dsRNA^18^.

In this study, we established a CPP library to screen for CPPs that efficiently penetrate plant suspension culture cells and intact plant leaves of monocots and dicots, including both of model plants and crops. We selected CPPs as a CPP library, based on previous reports on biophysical and biochemical analyses of CPPs as listed in Table 1. The CPPs selected here were reported as a high efficient CPP for animal cells. We determined optimal CPPs for wide-ranges of the plant species and tissues examined and investigated the relationship between cell penetration efficiency and CPP structural properties. As the efficiency of CPP-based delivery systems in plants varies among plant species and tissue types, each CPP should be optimized based on the target organism or tissue.

**Table 1:** Amino acid sequences and properties of CPPs used in this study.

Peptide No.	CPP	Amino acid sequence	Number of amino acids	CPP type^a^	Net charge at pH7	Reference
1	BP100	KKLFKKILKYL	11	A	5	^20^
2	2BP100	KKLFKKILKYLKKLFKKILKYL	22	C	10	^17^
3	Rev(34–50)	TRQARRNRRRRWRERQR	17	C	9	^47^
4	R9	RRRRRRRRR	9	C	9	^48^
5	D-R9	rrrrrrrrr (D form)	9	C	9	^49^
6	R12	RRRRRRRRRRRR	12	C	12	this study
7	KH9	KHKHKHKHKHKHKHKHKH	18	C	9.9	^50^
8	K9	KKKKKKKKK	9	C	9	this study
9	K18	KKKKKKKKKKKKKKKKKK	18	C	18	this study
10	Pen2W2F	RQIKIFFQNRRMKFKK	16	C	7	^51^
11	DPV3	RKKRRRESRKKRRRES	16	C	10	^52^
12	6-Oct	GRKRKKRT	8	C	6	^53^
13	R9-TAT	GRRRRRRRRRPPQ	13	C	9	^47^
14	Tat(49–57)	RKKRRQRRR	9	C	8	^49^
15	Retro - Tat(57–49)	RRRQRRKKR	9	C	8	^49^
16	Sc18	GLRKRLRKFRNKIKEK	16	A	8	^54^
17	KLA10	KALKKLLAKWLAAAKALL	18	A	5	^55^
18	IX	QLALQLALQALQAALQLA	18	H	0	^55^
19	XI	LKTLATALTKLAKTLTTL	18	H	3	^55^
20	No. 14–12	RAWMRWYSPTTRRYG	15	A	4	^56^
21	pVEC	LLIILRRRIRKQAHAHSK	18	A	6.2	^57^
22	PenArg	RQIRIWFQNRRMRWRR	16	A	7	^51^
23	M918	MVTVLFRRLRIRRACGPPRVRV	22	A	6.9	^58^
24	Penetratin	RQIKIWFQNRRMKWKK	16	A	7	^59^
25	PolyP 3 (SAP)	VRLPPPVRLPPPVRLPPP	18	H	3	^60^
26	dhvar5	LLLFLLKKRKKRKY	14	A	7	^61^
27	HPV33L2-445/467	SYFILRRRRKRFPYFFTDVRVAA	23	A	6	^62^
28	buforin II (5–21)	RAGLQFPVGRVHRLLRK	17	A	5.1	^63^
29	scrambled pVEC	IAARIKLRSRQHIKLRHL	18	A	6.2	^64^
30	HPV33L2-DD447	SYDDLRRRRKRFPYFFTDVRVAA	23	A	4	^62^
31	LAH4	KKALLALALHHLAHLALHLALALKKA	26	H	4.4	^65^
32	ppTG1	GLFKALLKLLKSLWKLLLKA	20	A	5	^66^
33	Transportan (TP)	GWTLNSAGYLLGKINLKALAALAKKIL	27	H	4	^67^
34	2 × ppTG1	GLFKALLKLLKSLWKLLLKAGLFKALLKLLKSLWKLLLKA	40	A	10	^15^
35	pAntpHD(Pro50)	RQIKIWFPNRRMKWKK	16	A	7	^68^
36	pAntp(44–58)	QIKIWFQNRRMKWKK	15	A	6	^69^
37	Crot(27–39)	KMDCRWRWKCCKK	13	A	4.8	^70^
38	Crot(27–39) derevative (1)	MDCRWRWKCCKK	12	A	3.8	^70^
39	Crot(27–39) derevative (2)	KCGCRWRWKCGCKK	14	A	5.7	^70^
40	CyLoP-1	CRWRWKCCKK	10	A	4.8	^70^
41	Inv3	TKRRITPKDVIDVRSVTTEINT	22	H	2	^71^
42	Inv5	AEKVDPVKLNLTLSAAAEALTGLGDK	26	H	-1	^72^
43	Inv3.5	TKRRITPKDVIDVRSVTTKINT	22	H	4	^72^
44	Inv3.10	HHHHHHTKRRITPKDVIDVRSVTTEINT	28	H	2.6	^72^
45	ARF(1–22)	MVRRFLVTLRIRRACGPPRVRV	22	A	6.9	^73^
46	Cyt C 77–101	GTKMIFVGIKKKEERADLIAYLKKA	25	A	4	^74^
47	hLF peptide	KCFQWQRNMRKVRGPPVSCIKR	22	A	6.9	^48^
48	Glu-Oct-6	EEEAAGRKRKKRT	13	A	3	^75^
49	M511	FLGKKFKKYFLQLLK	15	A	5	^76^
50	G53-4	FLIFIRVICIVIAKLKANLMCKT	23	H	3.9	^76^
51	M591	YIVLRRRRKRVNTKRS	16	A	8	^76^
52	E162	KTVLLRKLLKLLVRKI	16	A	6	^76^
53	E165	LLKKRKVVRLIKFLLK	16	A	7	^76^
54	M867	KKICTRKPRFMSAWAQ	16	A	4.9	^76^
55	MG2d	GIGKFLHSAKKWGKAFVGQIMNC	23	H	4	^77^

^a^C: cationic, A: amphipathic, H: hydrophobic.

## Results and Discussion

### The secondary structures of CPPs

**Figure 1:**
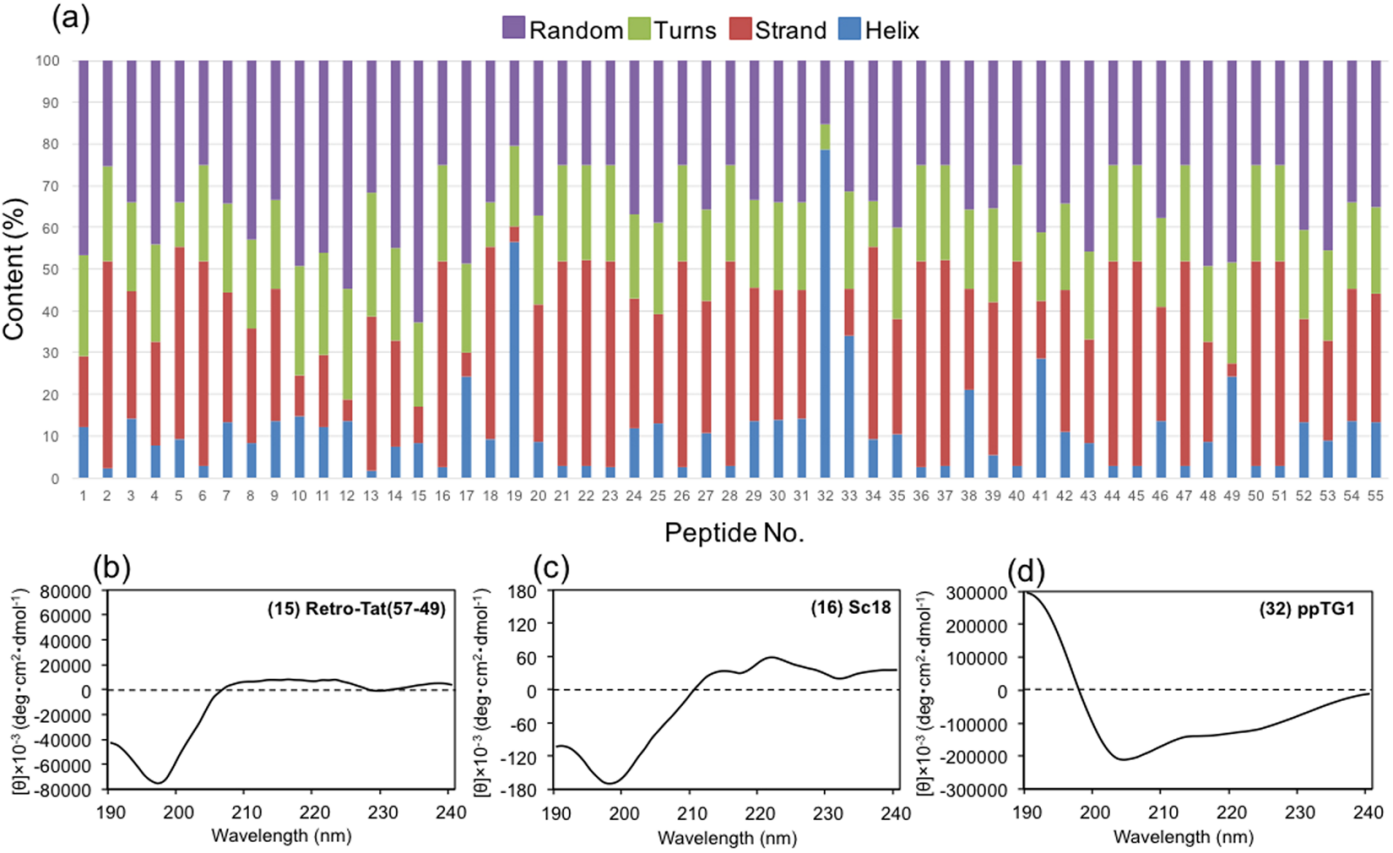
The secondary structures of 55 cell-penetrating peptides (CPPs). The secondary structure contents of CPPs determined by CD. (**b**) CD spectrum of Retro-Tat(57–49) (peptide No. 15) as a typical CD spectrum of a random coil structure-dominated CPP. (**c**) CD spectrum of Sc18 (peptide No. 16) as a typical CD spectrum of a beta-strand structure-dominated CPP. (**d**) CD spectrum of ppTG1 (peptide No. 32) as a typical CD spectrum of a helix structure-dominated CPP.

We prepared a CPP library composed of 55 CPPs (Peptide No. 1–55) labeled with 5-carboxytetramethylrhodamine (TAMRA) at the C-terminal end (Table 1). BP100 (KKLFKKILKYL), which we previously used in a CPP system to deliver genes into plant leaf cells of *A. thaliana* and *N. benthamiana*^19^, was used as the model CPP and Peptide No. 1. BP100 was originally designed and optimized as an antimicrobial peptide to protect against plant pathogens^20^. Some efficient CPPs have been modified and their derivatives were also reported. In those cases, we did not select the derivatives but the original CPP. This is because we needed to limit the number of CPP in the library. For example, KALA is one of the famous CPPs and KALA’s derivatives were also reported. However, in this study, we selected only KALA to limit the number of CPPs. Another reason to select CPPs is their type of CPPs, namely, hydrophobic, cationic and amphipathic. We tried to include those three types into the library. To study the relationship between structural properties and penetration efficiency of CPP, the secondary structures of CPPs were characterized by circular dichroism (CD). In this study, we focus on the structure and function of CPP without any cargo molecules. This is because CPP is widely used as a part of carrier molecules or particles without direct interactions between CPP and cargo molecules^14,21^. Based on the CD spectra (Fig. S1), the secondary structures of CPPs were calculated by the DichroWeb online CD analysis server using SELCON3 algorithms (Fig. 1a). Retro-Tat (Peptide No. 15) had very low ellipticity above 210 nm and negative bands near 195 nm (Fig. 1b) and hence contained the highest random coil content of greater than 60%. The beta-strand content was notably different among CPPs, ranging from 0–50%. The highest beta-strand content was nearly 50% in Sc18 (Peptide No. 16), which had negative bands at 218 nm and positive bands at 195 nm (Fig. 1c). ppTG1 (Peptide No. 32) had negative bands at 222 nm and 208 nm and a positive band at 193 nm and therefore showed the highest helix content of nearly 80% (Fig. 1d). Cationic CPPs containing Lys and Arg formed proportionally more random coils, and amphipathic CPPs consisting of alternating sequences of polar and non-polar amino acids formed proportionally more helices. The CD spectra of the poor water-soluble CPPs, IX (Peptide No. 18), 2 × ppTG1 (Peptide No. 34) and G53-4 (Peptide No. 50), were also obtained (Fig. S1), indicating that those CPPs were dissolved into water via 2.5% DMSO.

### Cell penetration efficiency into BY-2 cells

**Figure 2:**
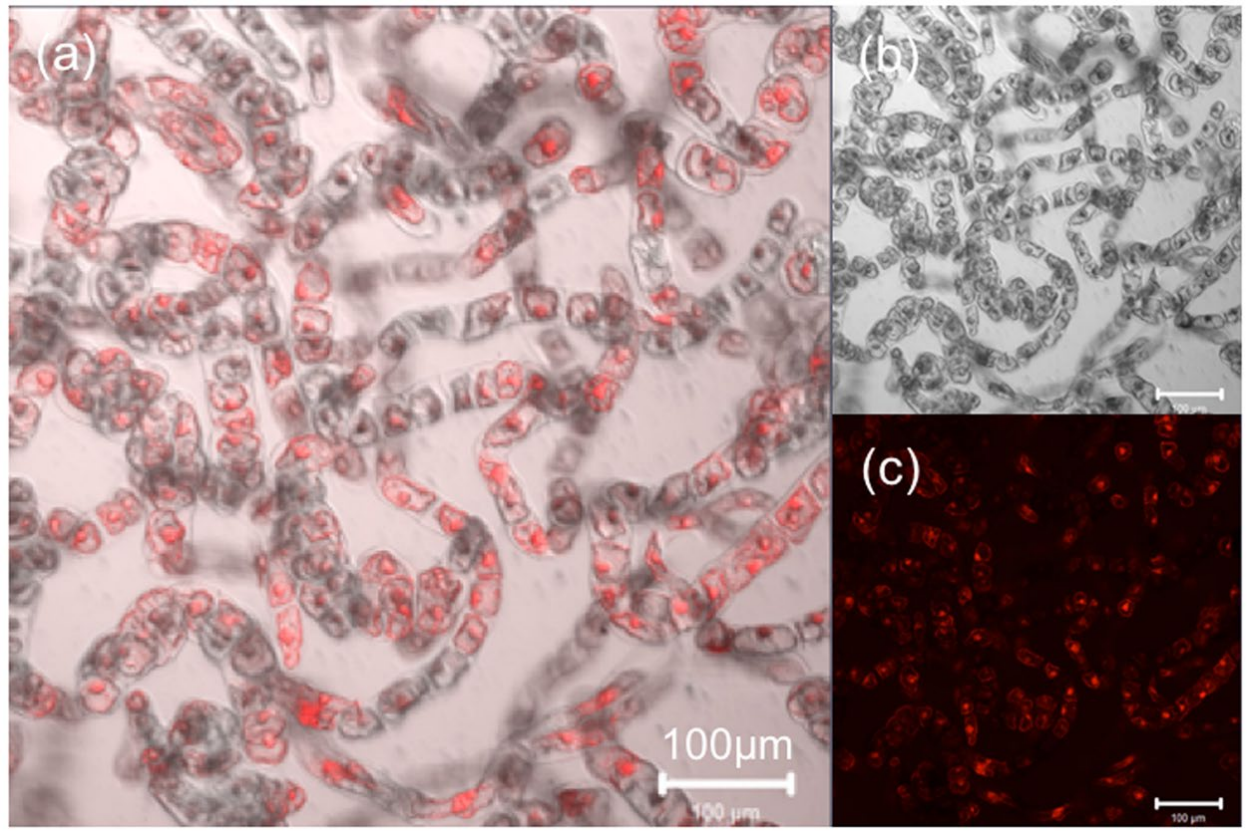
BY-2 cells after incubation with TAMRA-labelled BP100 labeled for 3 h at 26 °C. (**a**) Overlay image, (**b**) DIC image, and (**c**) TAMRA fluorescence image generated by CLSM. Scale bar, 100 µm.

**Figure 3:**
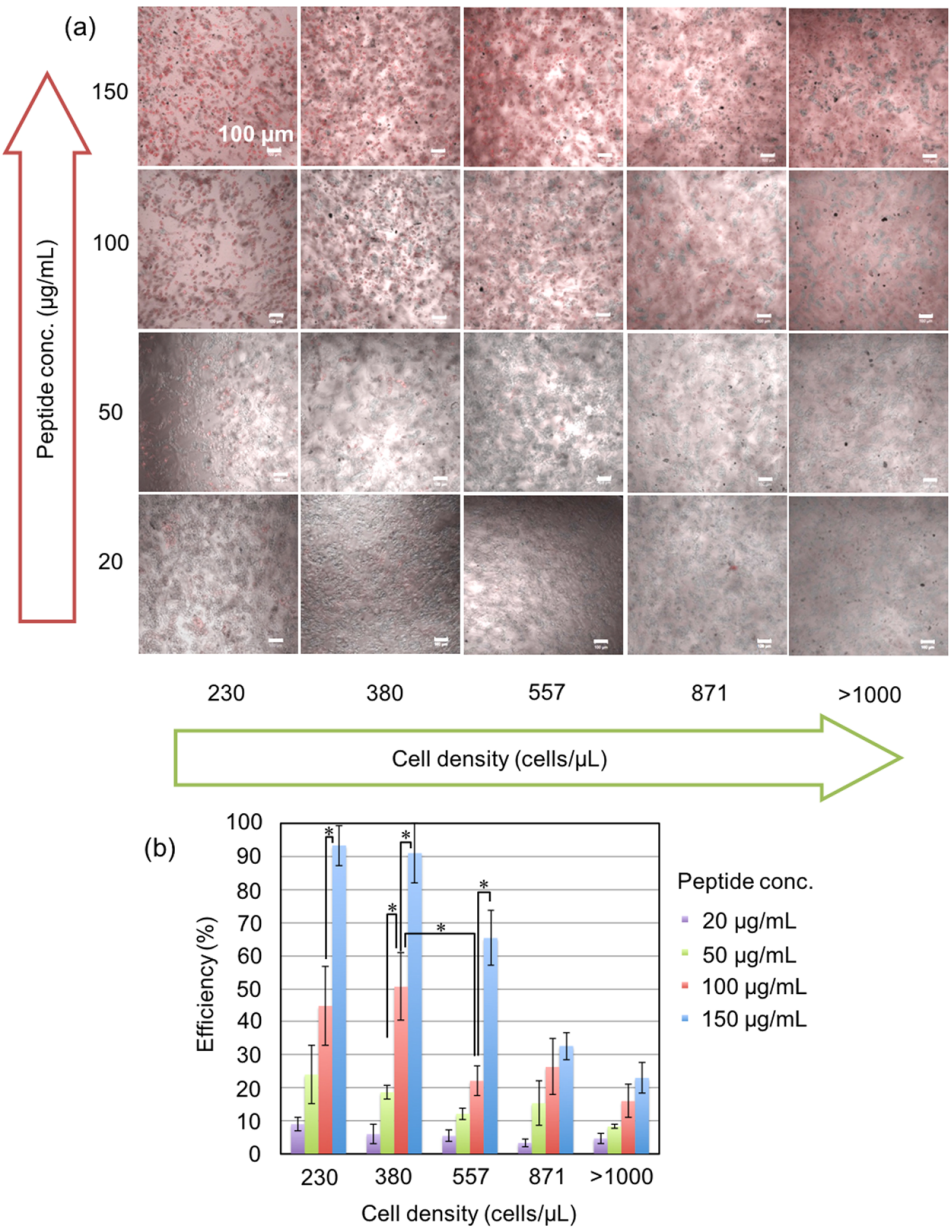
Effects of peptide concentrations and BY-2 cell densities on cell penetration efficiency. (**a**) CLSM overlay images of BY-2 cells treated with TAMRA-labeled BP100. Red signals indicate TAMRA fluorescence. The peptide concentration ranged from 20 to 150 µg/mL. The cell density ranged from 230 to > 1000 cells/µL. (**b**) Cell penetration efficiency of BP100 at different peptide concentrations and BY-2 cell densities. The efficiencies were determined using CLSM images. The error bars show standard deviations of three independent experiments (Tukey’s HSD test, *P < 0.05).

To evaluate the cell penetration efficiency of the 55 CPPs into BY-2 suspension culture cells, we first optimized the CPP concentration and cell density using BP100 as a model CPP. The cell penetration efficiency of CPP into BY-2 cells was determined based on the ratio of numbers of cell with and without TAMRA signals of CPP by confocal laser scanning microscopy (CLSM) (Fig. 2). BY-2 cells showed different peptide uptake depending on the culture time after subculture (Fig. S2a–f). The BY-2 cells at 3 days after subculture, which are at the log growth phase, resulted in higher cell penetrating efficiency of the model peptide, BP100, in comparison to the BY-2 cells at 1 day and 7 days after subculture. In addition, the incubation time of BY-2 cells with CPP was investigated from 1 hour to 96 hours (Fig. S2g–n), resulting that 3 hours were sufficient to detect the cell penetration of TAMRA-labeled CPP. Thus, we used the BY-2 cells at 3 days after subculture as well as the incubation time of 3 hours for all the experiments in this study. The efficiency of cell penetration increased with decreasing cell density and increasing peptide concentration (Fig. 3). We used 100 µg/mL CPP concentration and approximately 380 cells/µL, which provided approximately 50% cell penetration efficiency of BP100 as the baseline, to examine the efficiencies of other CPPs.

**Figure 4:**
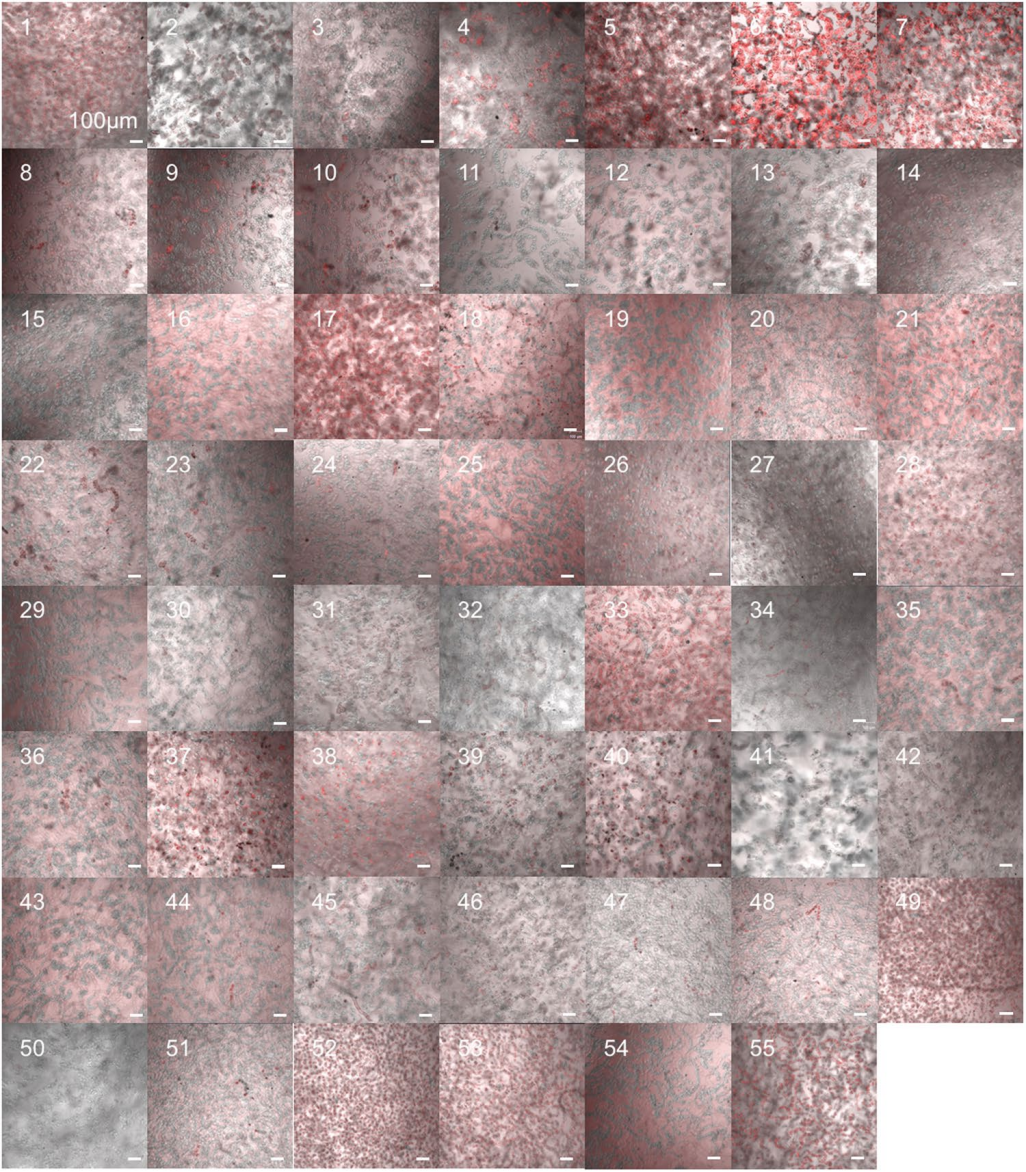
CLSM images of BY-2 cells incubated with 55 types of CPP for 3 h at 26 °C. The images are overlays of DIC (gray scale) and TAMRA fluorescence (red). The type of CPP is shown as peptide number as listed in Table 1. The peptide concentration was 100 µg/mL. The cell density was approximately 380 cells/µL. Scale bars, 100 µm.

**Figure 5:**
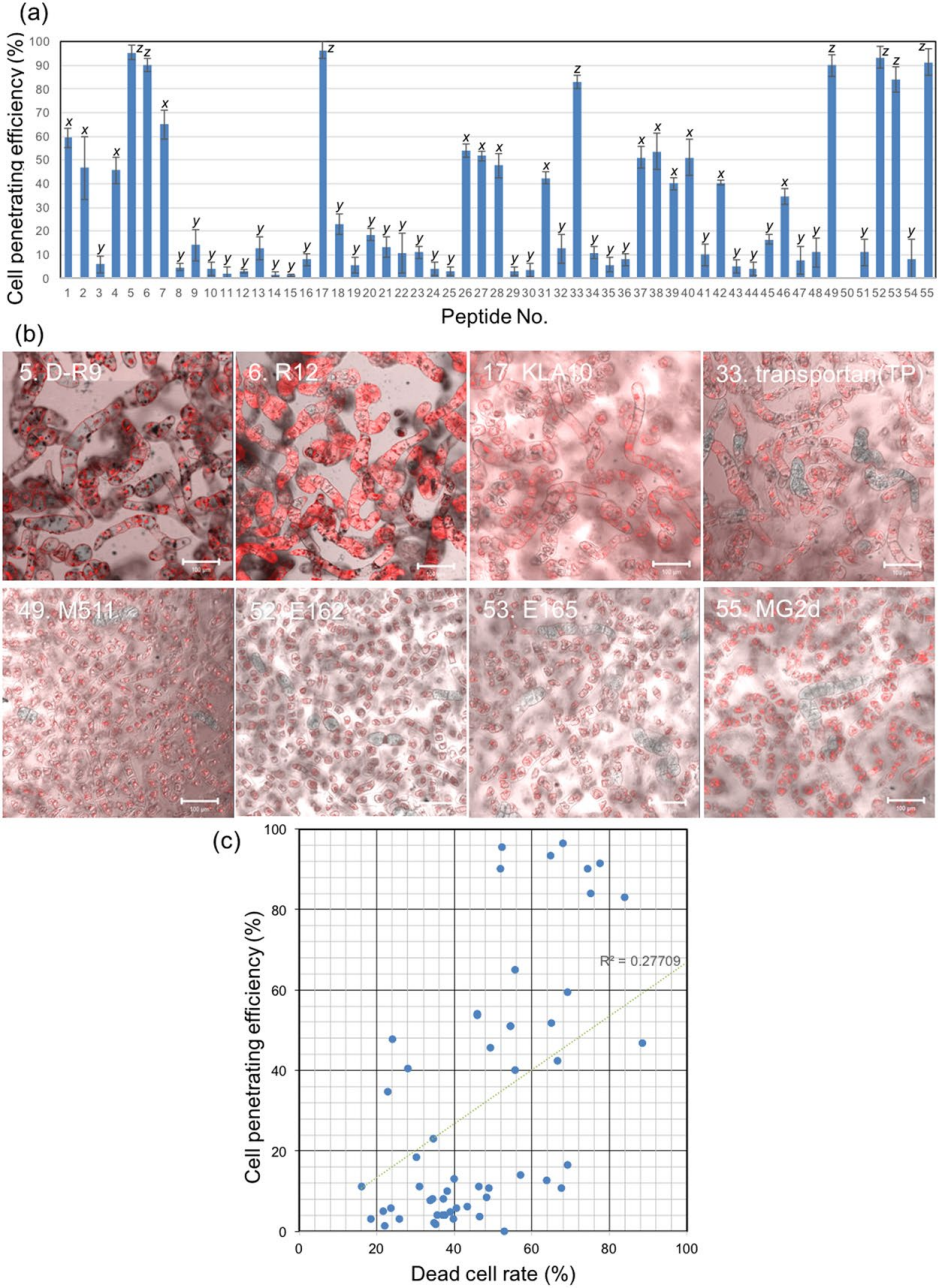
Cell penetration efficiency of the CPP library. (**a**) BY-2 cell penetration efficiency of 55 types of CPPs. The type of CPP is shown as peptide number and is listed in Table 1. BY-2 cell was evaluated after a 3-h incubation at 26 °C. For cell penetration, the peptide concentration and cell density were 100 µg/mL and approximately 380 cells/µL, respectively. All data are expressed as the means ± S.D. from triplicate tests; the mean data labeled with different letters (*x*, *y*, and *z*) are significantly different (Tukey’s HSD test, *P* < 0.05). (**b**) CLSM images of the eight CPPs with the highest cell penetration efficiency. The images are overlays of DIC (gray scale) and TAMRA fluorescence (red). Scale bars, 100 µm. (**c**) Relationship between the cell penetrating efficiency and dead cell rate of 55 types of CPPs. The dead cell rates are converted from Fig. S5.

The peptide library was assayed with BY-2 cells under the optimal conditions determined above (Figs 4 and S3). As a negative control without CPP, TAMRA molecules could not penetrate into BY-2 cells up to 6 hours (Fig. S4). Based on CLSM images, the cell penetration efficiencies of the 55 CPPs into BY-2 suspension culture cells were determined and compared to that of BP100 (Fig. 5a). Eight CPPs, namely, D-R9 (Peptide No. 5), R12 (Peptide No. 6), KLA10 (Peptide No. 17), Transportan (Peptide No. 33), M511 (Peptide No. 49), E162 (Peptide No. 52), E165 (Peptide No. 53), and MG2d (Peptide No. 55) (Fig. 5b), showed significantly greater efficiencies than BP100 (Peptide No. 1). Although there were no obvious similarities in the secondary structures of those CPPs (Fig. 1a and Table 1), those eight efficient CPPs did not show high helix content. This tendency related to the secondary structures indicates that predominant helical structures are not essential to penetrate into plant cells. D-R9 (Peptide No. 5), KLA10 (Peptide No. 17), IX (Peptide No. 18), M511 (Peptide No. 49), E162 (Peptide No. 52), E165 (Peptide No. 53), and MG2d (Peptide No. 55) penetrated cells and preferentially localized in the nucleus (Fig. 5b). These CPPs could function as nuclear localizing signals in addition to cell-penetrating motifs. Notably, M511 (Peptide No. 49), E162 (Peptide No. 52), E165 (Peptide No. 53), and MG2d (Peptide No. 55) caused signs of plasmolysis, i.e., the cell membrane seemed to separate from the cell wall. On the other hand, D-R9 (Peptide No. 5), R12 (Peptide No. 6), KLA10 (Peptide No. 17), and Transportan (Peptide No. 33) did not (Fig. 5b). Plasmolysis generally inhibits cellular activity and viability after CPP treatment, implying that those CPPs might potentially be cytotoxic to plant cells. The cytotoxicity of CPPs was evaluated in terms of BY-2 cell viability by Evans blue assay (Fig. S5a). 2BP100 (Peptide No. 2), ppTG1 (Peptide No. 32) and Transportan (Peptide No. 33) showed relatively higher cytotoxicity to BY-2 cells. The relationship between the cell penetrating efficiency and dead cell rate of 55 peptides is summarized in Fig. 5c, indicating weak correlation between the cell penetrating efficiency and dead cell rate. DMSO of 2.5% for the poor water-soluble CPPs did not show cytotoxicity based on the Evans blue assay (Fig. S5b). Taken together, D-R9 (Peptide No. 5), R12 (Peptide No. 6) and KLA10 (Peptide No. 17) were defined as highly efficient CPPs without lethal toxicity for BY-2 cells. D-R9 (Peptide No. 5) and R12 (Peptide No. 6) are Arg-containing cationic CPPs, while KLA10 (Peptide No. 17) are Lys-containing amphipathic CPPs. We evaluated the relationship between cell penetration efficiency into BY-2 cells and the number of Lys or Arg residues in CPP sequences (Fig. S6). However, no obvious associations between those cationic amino acid residues and the efficiency, indicating that no essential amino acids to achieve penetration of CPPs into BY-2 cells.

**Figure 6:**
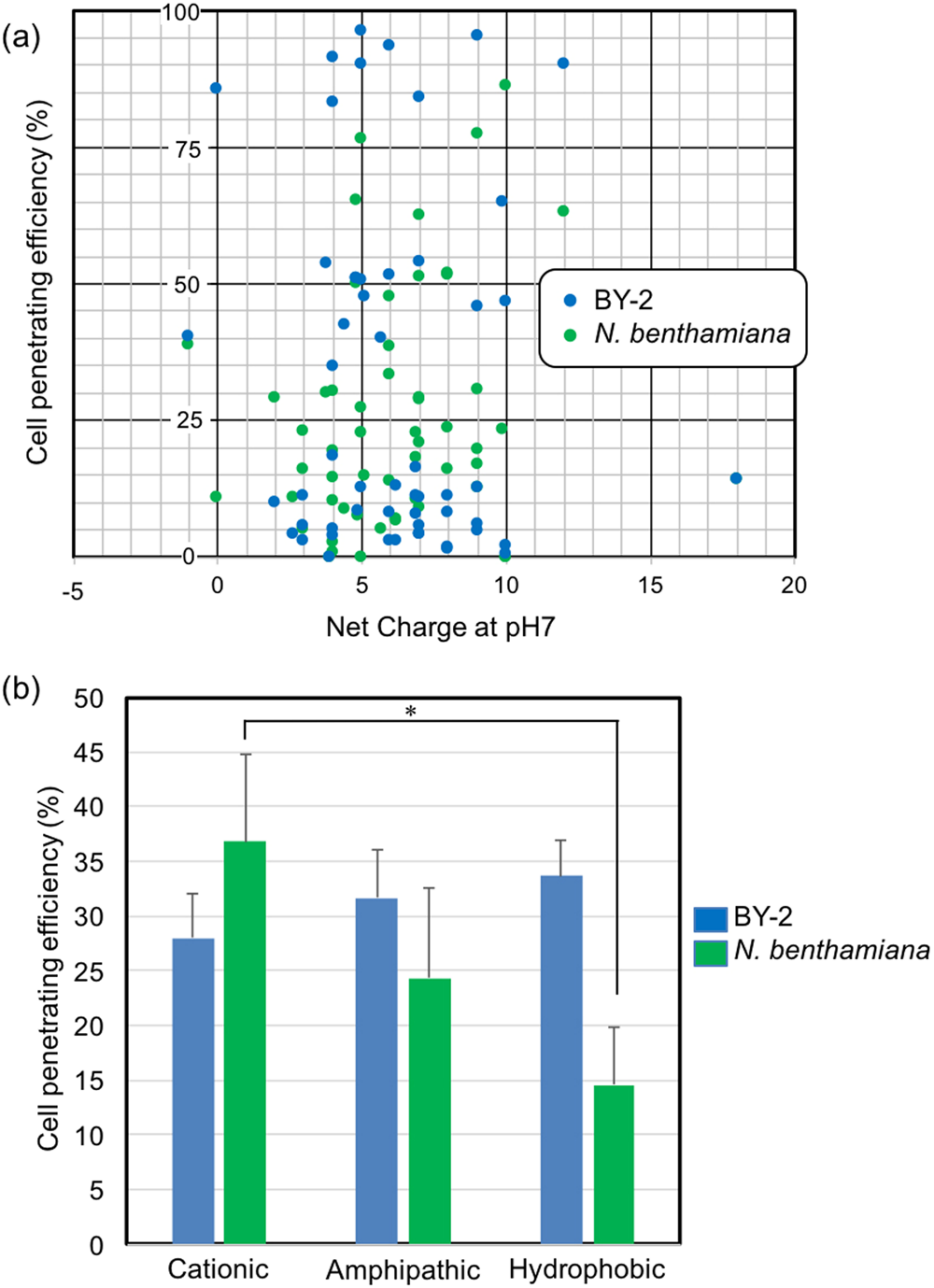
Effects of net charges at pH 7 and types of CPPs on cell penetration efficiencies into BY-2 cells and *N. benthamiana* leaves. (**a**) Relationship between net charges at pH 7 of 55 types of CPPs and the cell penetration efficiency. (**b**) The cell penetration efficiencies of cationic, amphipathic, and hydrophobic CPPs. The CPP types are listed in Table 1. All data are expressed as the means ± S.D. from triplicate tests; the mean data labeled with different letters (*x*, *y*, and *z*) are significantly different (Tukey’s HSD test, *P* < 0.05).

We next evaluated the effect of net charge of CPPs at pH 7 on the cell penetration efficiency into BY-2 cells (Fig. 6a). The net charge of CPPs was roughly related to the type of CPP; cationic, amphipathic, and hydrophobic CPPs tended to have high, medium, and low net charges, respectively (Table 1). However, we did not detect any correlations between the net charges of CPPs and their penetration efficiency into BY-2. Furthermore, we compared the average cell penetration efficiencies of cationic, amphipathic, and hydrophobic CPPs; however, we did not detect any significant difference between the three CPP types in BY-2 (Fig. 6b). Thus, we did not detect a relationship between the chemical properties of CPPs and cell penetration efficiency in the case of BY-2 cells.

### Cell penetration efficiency into *N. benthamiana* epidermal cells

**Figure 7:**
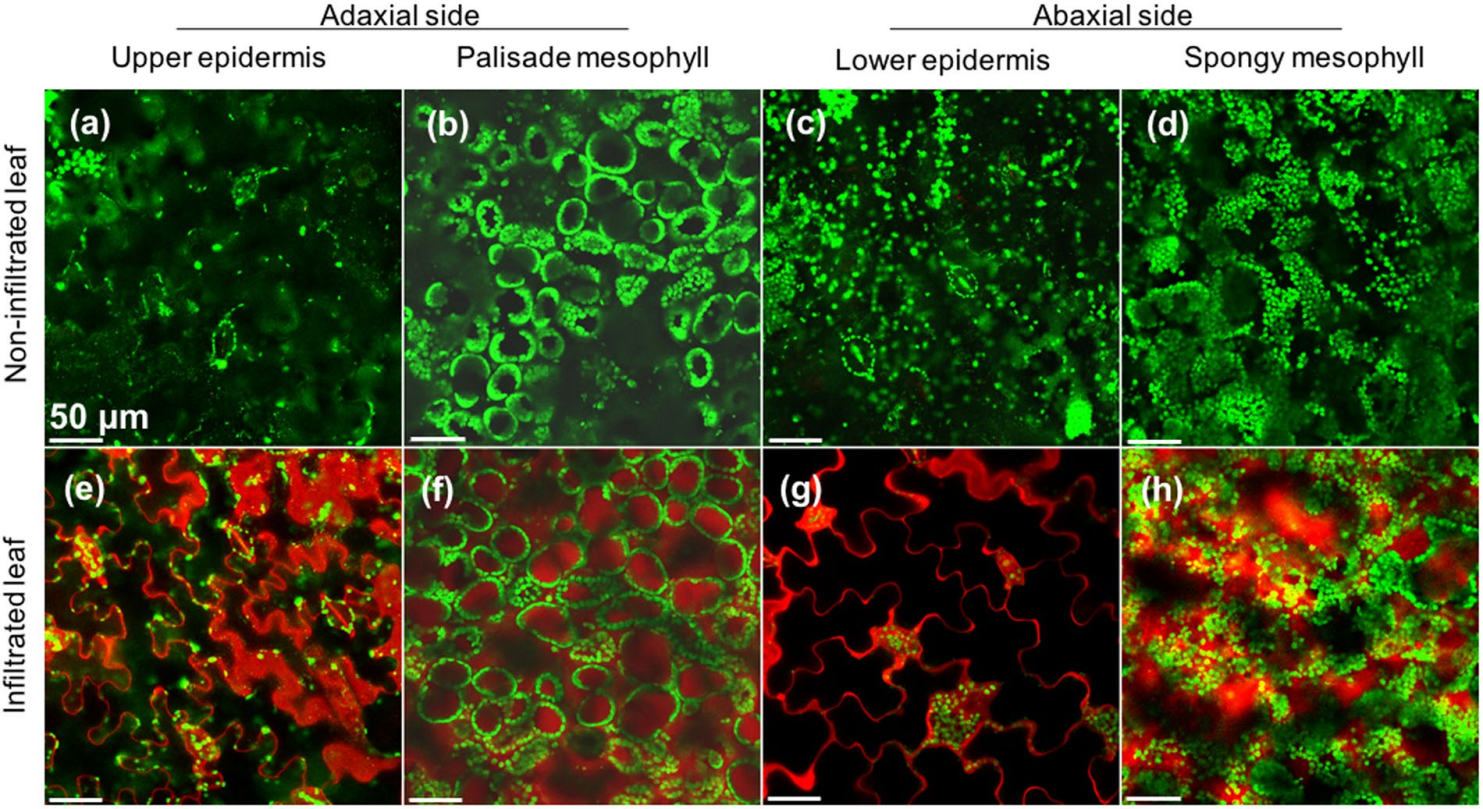
Infiltration of TAMRA-labeled BP100 (Peptide No. 1) from adaxial to abaxial side of *Nicotiana benthamina* leaf surface. The BP100 is introduced from abaxial surface of 2-weeks-old *N. benthamina* leaf by infiltration. After the infiltrated plant were cultured for 3 h at 23 °C in dark, the leaf section was used for CLSM analysis after deairation. (**a**–**d**) The non-infiltrated and (**e**–**h**) filtrated leaves (**e**–**h**) were imaged by CLSM to obtain single images of the TAMRA-labeled BP100 (red) and chloroplast (green). Images show the leaf epidermal (**a**,**c**,**e**,**g**) and mesophyll cells (**b**,**d**,**f**,**h**) in both adaxial (**a**,**b**,**e**,**f**) and abaxial side (**c**,**d**,**g**,**h**) of the leaves.

**Figure 8:**
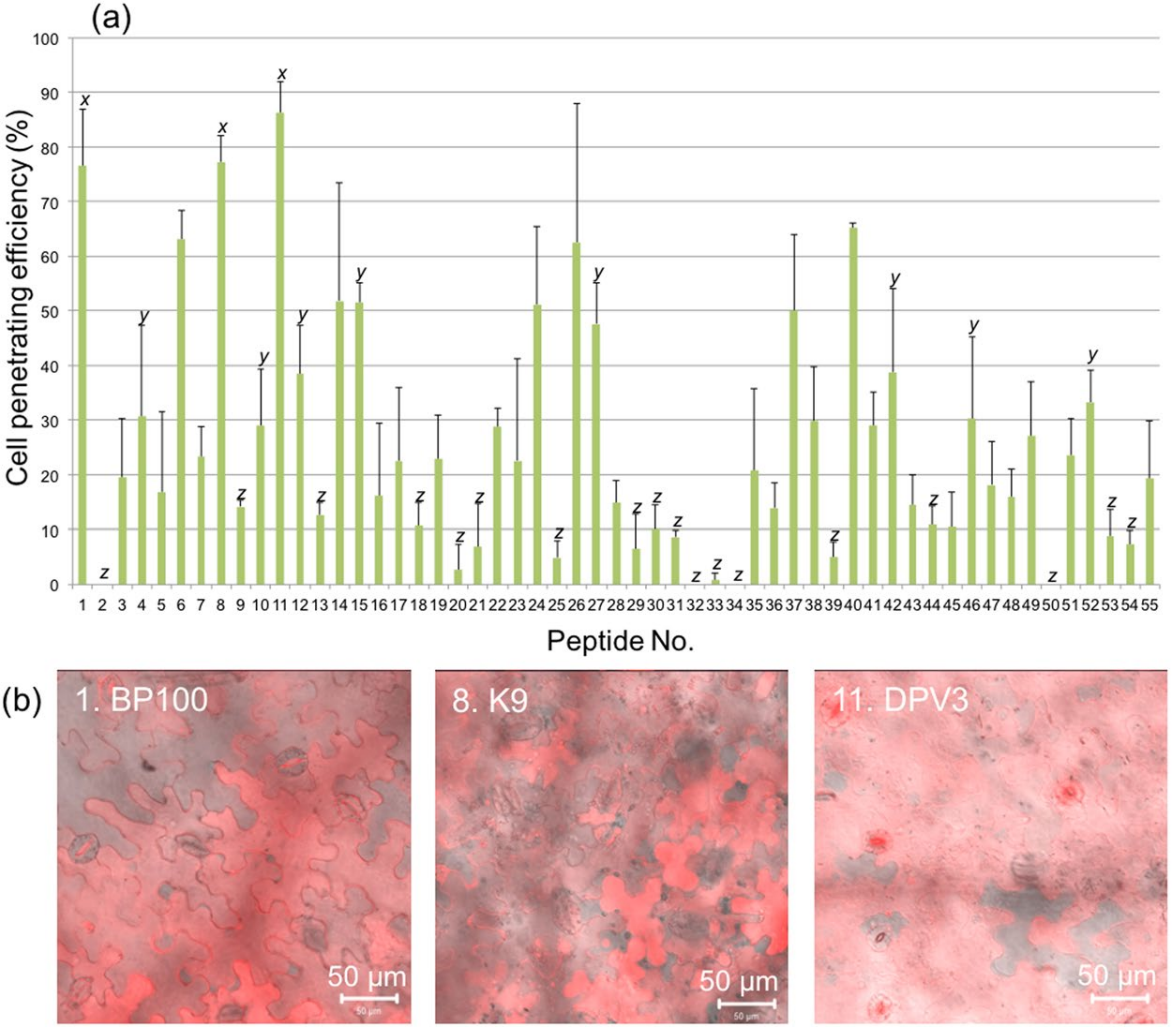
Cell penetration efficiency into *N. benthamiana* leaf epidermal cells by 55 CPPs. (**a**) The efficiency of penetration determined by counting the penetrated cells at 2 h after infiltration at 30 °C. All data are expressed as the means ± S.D. from triplicate tests; the means labeled with different letters (*x*, *y*, and *z*) are significantly different (Tukey’s HSD test, *P* < 0.05). (**b**) CLSM images of the three CPPs with the highest cell penetration efficiency into *N. benthamiana* leaf epidermal cells. The CLSM images are overlay of TAMRA fluorescence signal (red) and DIC.

To evaluate the penetration efficiency into intact plant leaves, we assayed the ability of CPPs in the library to penetrate *N. benthamiana* leaves (Fig. S7). The conditions for infiltration of each CPP into *N. benthamiana* leaves were determined according to a previous study^19^. MilliQ water was used as the solvent for CPP infiltration as reported previously^22^. A model CPP, BP100 (Peptide No. 1), penetrated into *N. benthamiana* leaves from lower to upper epidermis cells together with mesophyll cells (Fig. 7). The cell penetrating efficiencies into cotyledons and true leaves were not significantly different based on the CLSM images (Fig. S8). Thus, the cell penetration efficiency into *N. benthamiana* was calculated by counting the numbers of fully-stained and non-stained epidermal cells in the true leaves. The non-stained cells included cells whose cell membrane was stained but cytosol/vacuole was not. We did not include guard cells in the calculation of penetration efficiency, because guard cells are readily stained and penetrated. CLSM images (Fig. S9) were quantitatively analyzed to determine the cell penetration efficiency of each CPP (Fig. 8a). The highly efficient CPPs for BY-2 cells were not the most efficient CPPs in *N. benthamiana* leaves, even though the efficiency of R12 (Peptide No. 6), one of the most efficient CPPs in BY-2 cells, was over 60%. These different results between BY-2 cells and *N. benthamiana* leaves indicated that the cell penetrating efficiency into a leaf might be under the influence of the intracellular connection and anatomic structure of leaves. On the contrary, similar to the assays with BY-2 cells, several CPPs containing cationic amino acids could function as nuclear localizing signals in addition to cell-penetrating motifs. Three CPPs, BP100 (Peptide No. 1), K9 (Peptide No. 8), and DPV3 (Peptide No. 11), demonstrated relatively high penetration efficiency (approximately 80%) in *N. benthamiana* leaves (Fig. 8a,b). BP100 (Peptide No. 1) is amphipathic but K9 (Peptide No. 8) and DPV3 (Peptide No. 11) are cationic CPPs. The similarity among those three efficient CPPs is Lys-rich sequences. BP100 (Peptide No. 1), K9 (Peptide No. 8), and DPV3 (Peptide No. 11) contain 5, 9 and 4 Lys residues, whereas BP100 (Peptide No. 1) and K9 (Peptide No. 8) lack Arg. Therefore, Lys residues appear to increase the cell penetration efficiency of CPPs targeting *N. benthamiana* leaves.

To discuss the effects of Lys and Arg residues on cell penetrating efficiency, we need to consider previous reports on CPPs with animal cells. In the case of mammalian cells, polyLys-based CPPs are also efficient and interact with membrane lipid head groups to induce wrapping of the membrane monolayers^23^. However, the roles and effects of Arg and Lys are different. Guanidium group of Arg plays a stronger structural effect than ammonium group of Lys in the lipid-assisted translocation of CPPs^24^. In addition, Arg-rich peptides, such as the Tat peptide, which originated from the HIV transactivator protein, are considered to be among the most efficient CPPs^25,26^. Arg-rich CPPs may promote cell penetration by generating negative Gaussian membrane curvature, which is generally found in pores, protrusions from macropinocytosis, and endocytosis^27^. The difference between Lys and Arg interactions with lipids originates from the side chain functional groups. However, the cell penetration efficiency of CPPs containing polyArg is reportedly higher than those containing polyLys when their molecular weights are similar^23^. In contrast to findings in animal cells, this study and previous research in plants indicate that Arg-rich CPPs are not the most efficient at penetrating plant cells^28–31^. Arg-rich CPP was reported to interact with proteoglycans and lipids of the cell membranes^32^, suggesting that the difference in membrane compositions between the plant and animal cells could affect the effects of Lys and Arg. The major lipid components of plant cell membranes are phospholipids, glycolipids, and sterols^33,34^, whereas the membranes of animal cells additionally contains cholesterol as a major component^35^. As well, it is possible that proteoglycans in plant cell wall could affect the behavior of Arg-rich CPP. Further studies should examine how the CPP internalization mechanisms differ between plant and animal cells.

### Effect of plant species on cell penetration efficiency

**Figure 9:**
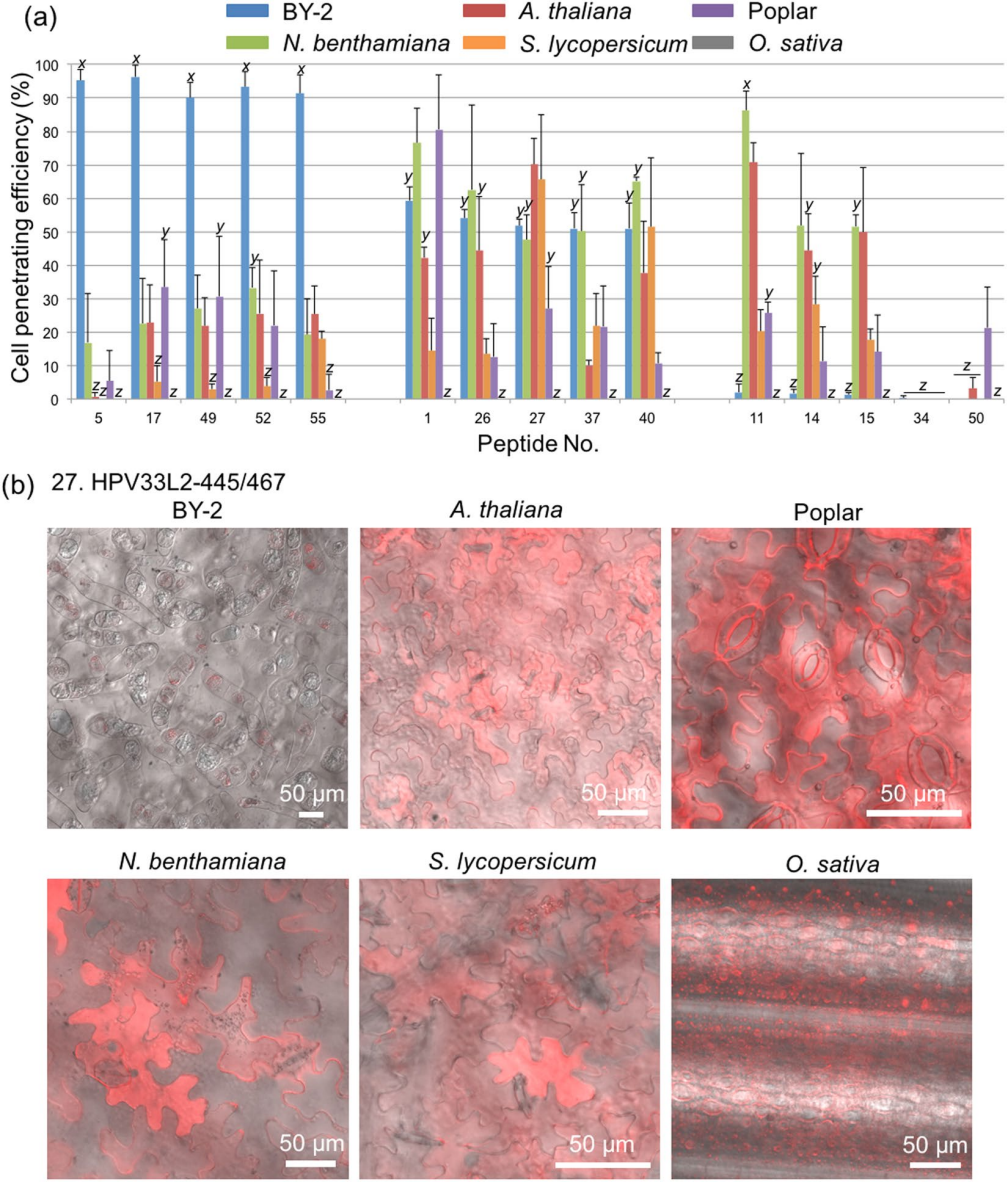
Comparison of cell penetration efficiency by 15 CPPs into different plant species. Based on the results from BY-2 cells, five high efficiency CPPs (peptide No. 5, 17, 49, 52, and 55), five medium efficiency CPPs (peptide No. 1, 26, 27, 37, and 40), and five low efficiency CPPs (peptide No. 11, 14, 15, 34, and 50) were selected. In addition to BY-2 cells, leaves of *N. benthamiana*, *A. thaliana*, Moneymaker tomato, poplar, and rice were used. (**a**) Cell penetration efficiency of 15 CPPs. All data are expressed as the means ± S.D. from triplicate tests; the mean data labeled with different letters (*x*, *y*, and *z*) are significantly different (Tukey’s HSD test, *P* < 0.05). (**b**) CLSM images of six plant species treated with a TAMRA-labeled CPP (Peptide No. 27. HPV33L2-445/467). The CLSM images are overlay of TAMRA fluorescence signal (red) and DIC.

In addition to BY-2 cells and *N. benthamiana* leaves, *Arabidopsis thaliana*, *Solanum lycopersicum* (Moneymaker tomato), hybrid aspen *Populus tremula* × *tremuloides* line T89 (poplar), and *Oryza sativa* (rice) leaves were assayed to study the cell penetration efficiency of each CPP across species. We selected five high, five medium, and five low efficiency CPPs from the BY-2 cell results for these assays. The most efficient CPPs were D-R9 (Peptide No. 5), KLA10 (Peptide No. 17), M511 (Peptide No. 49), E162 (Peptide No. 52), and MG2d (Peptide No. 55). The five medium efficiency CPPs were BP100 (Peptide No. 1), dhvar5 (Peptide No. 26), HPV33L2-445/467 (Peptide No. 27), Crot(27–39) (Peptide No. 37), and CyLoP-1 (Peptide No. 40). The least efficient CPPs were DPV3 (Peptide No. 11), Tat(49–57) (Peptide No. 14), Retro-Tat(57–49) (Peptide No. 15), 2 × ppTG1 (Peptide No. 34), and G53-4 (Peptide No. 50). The epidermal cells of *A. thaliana*, Moneymaker tomato, and poplar tree were observed by CLSM at 3 h after the infiltration of CPP solutions were infiltrated into the leaves. Using the CLSM images taken after infiltration (Figs S10, S11 and S12), the cell penetration efficiency was calculated for each of the 15 CPPs in each species (Fig. 9a, Table S1), as the case of *N. benthamiana* leaves. HPV33L2-445/467 (Peptide No. 27) showed intermediate efficiency of penetration into all the tested plant species except *O. sativa* (Fig. 9a,b). Any CPPs evaluated in this study did not penetrate the epidermal cells of rice, *O. sativa*. Although TAMRA signals were detected at the surface of rice leaves, staining was absent from the cytosol of the epidermal cells. BP100 (Peptide No. 1) also efficiently penetrated BY-2 cells and *N. benthamiana*, *A. thaliana*, and poplar leaves, but did not penetrate the epidermal cells of rice. Even though rice was the only monocot studied here, this result implies that dicots may be more amenable to penetration by CPPs than monocots. Perhaps the silica layer surrounding rice cells in addition to the cuticle resists internalization of CPPs^36^.

**Figure 10:**
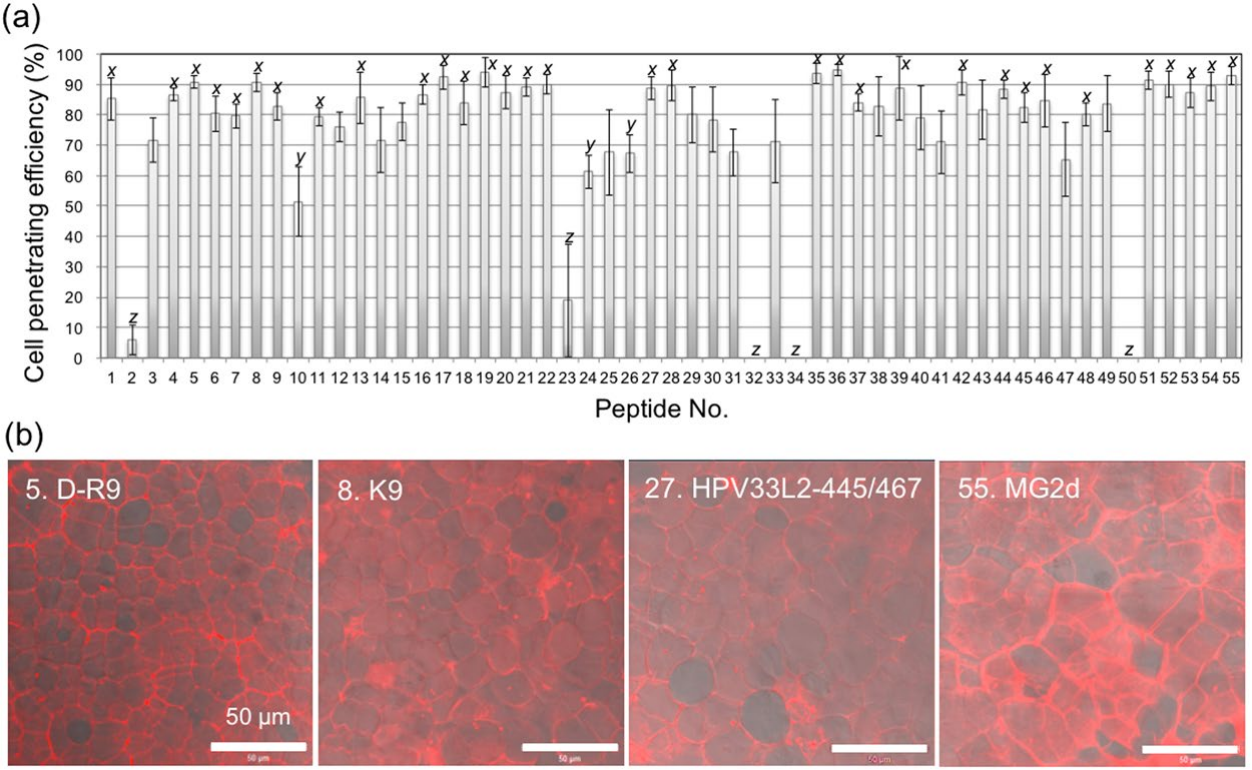
Cell penetration efficiency into rice (*O. sativa*) callus by 55 CPPs. (**a**) The efficiency of penetration determined by counting the penetrated cells at 2 h after infiltration at 30 °C. All data are expressed as the means ± S.D. from triplicate tests; the means labeled with different letters (*x*, *y*, and *z*) are significantly different (Tukey’s HSD test, *P* < 0.05). (**b**) CLSM images of the four CPPs of the highest cell penetration efficiency into the rice callus. The CLSM images are overlay of TAMRA fluorescence signal (red) and DIC.

To clear the effect of rice leaf structure, rice callus was assayed with 55 CPPs. Rice callus with a diameter of approximately 1 mm was treated with CPP solution. At 3 hours after the treatment, the callus was imaged by CLSM (Fig. S13). The penetrating efficiency into rice callus was determined based on the ratio of numbers of cell with and without TAMRA signals of CPP (Fig. 10a). Many CPPs showed relatively high penetration efficiency (over 80%), and also nine CPPs demonstrated penetration efficiency of over 90%. Based on the CLSM images (Fig. 10b), efficient CPPs penetrated into rice callus homogeneously and induced no obvious cytotoxicity.

**Figure 11:**
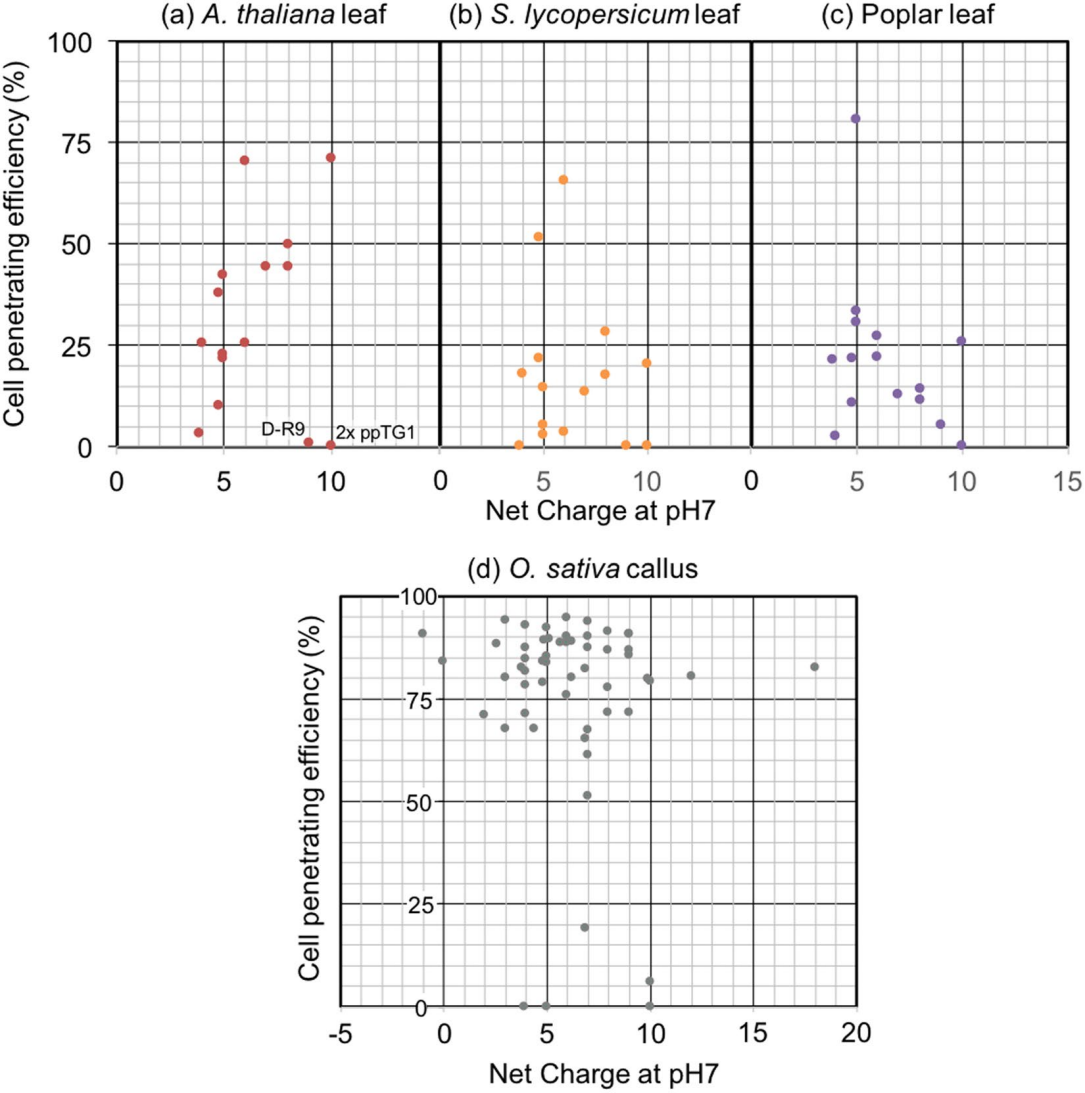
Relationship between net charges at pH 7 of CPPs and cell penetration efficiencies into leaf epidermal cells. *A. thaliana* (**a**), Moneymaker tomato (**b**), and poplar (**c**) epidermal cells 2 h after infiltration by the indicated CPPs.

We next examined the relationship between the net charges of CPPs and cell penetration efficiencies into *A. thaliana*, Moneymaker, and poplar leaves as well as rice callus, because net charges influence the chemical properties of the CPPs (Fig. 11). However, we did not detect any trends between the net charges and efficiency of penetration for the plant species studied. In the case of *A. thaliana* leaves, D-R9 (net charge 9) and 2 × ppTG1 (net charge 10) largely failed to penetrate cells and are outliers among the CPPs (Fig. 11a). CPPs with low water solubility such as 2 × ppTG1 (Peptide No. 34) and G53-4 (Peptide No. 50) showed low cell penetration efficiencies across most species (Fig. 11b,c). This might be because these CPPs cannot function as expected based on the amino acid sequence as they form non-specific aggregations. Although the leaf of rice, *O. sativa*, was not penetrated efficiently by any CPPs, many CPPs showed efficient penetrating efficiency into rice callus. We investigated the relationship between the net charges of CPPs and penetration efficiency into rice callus (Fig. 11d), which cleared the electrical charge of CPPs was not critical to the cell penetrating efficiency. Furthermore, we could conclude that rice callus is appropriate material for CPP-mediated introductions.

**Figure 12:**
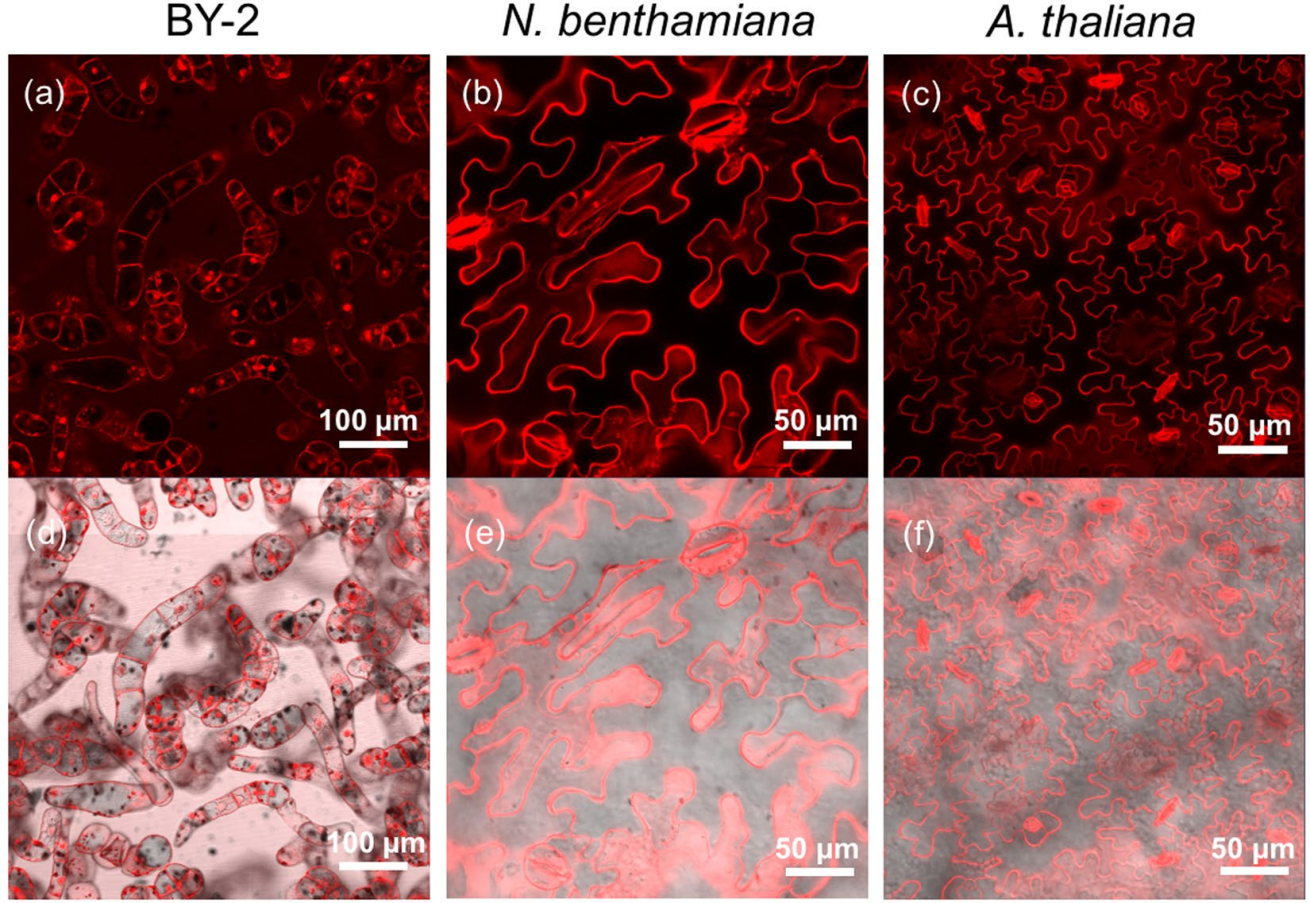
CLSM images of various targets infiltrated with TAMRA-labeled CPP peptide No. 5 D-R9. BY-2 cells (**a**,**d**) and *N. benthamiana* (**b**,**e**) and *A. thaliana* (**c**,**f**) leaf epidermal cells 2 h after infiltration. (**a**–**c**) TAMRA fluorescence images. (d–f) Overlay images of TAMRA fluorescence and DIC images.

D-R9 (Peptide No. 5) was the only peptide in this experiment that was composed of D-form amino acids. If the cell membrane biologically recognizes the amino acid sequences of CPPs, unnatural D-form amino acids would prevent cellular uptake through the membrane. We found that D-R9 exhibited a high efficiency of penetration into BY-2 cells, but a low efficiency of penetration into plant leaves (Figs 5b and 12a,d). However, D-R9 bound preferentially to the membrane and did not penetrate the cytosol or vacuole (Fig. 12b,c,e,f). Thus, binding efficiency might be determined by electrical net charges, but the penetration efficiency might be related to biological interactions between L-Arg and plant cell membranes. The molecular mechanism underlying this phenomenon of D-form amino acids’ binding and penetration behaviors is one of the further targets of investigation.

**Figure 13:**
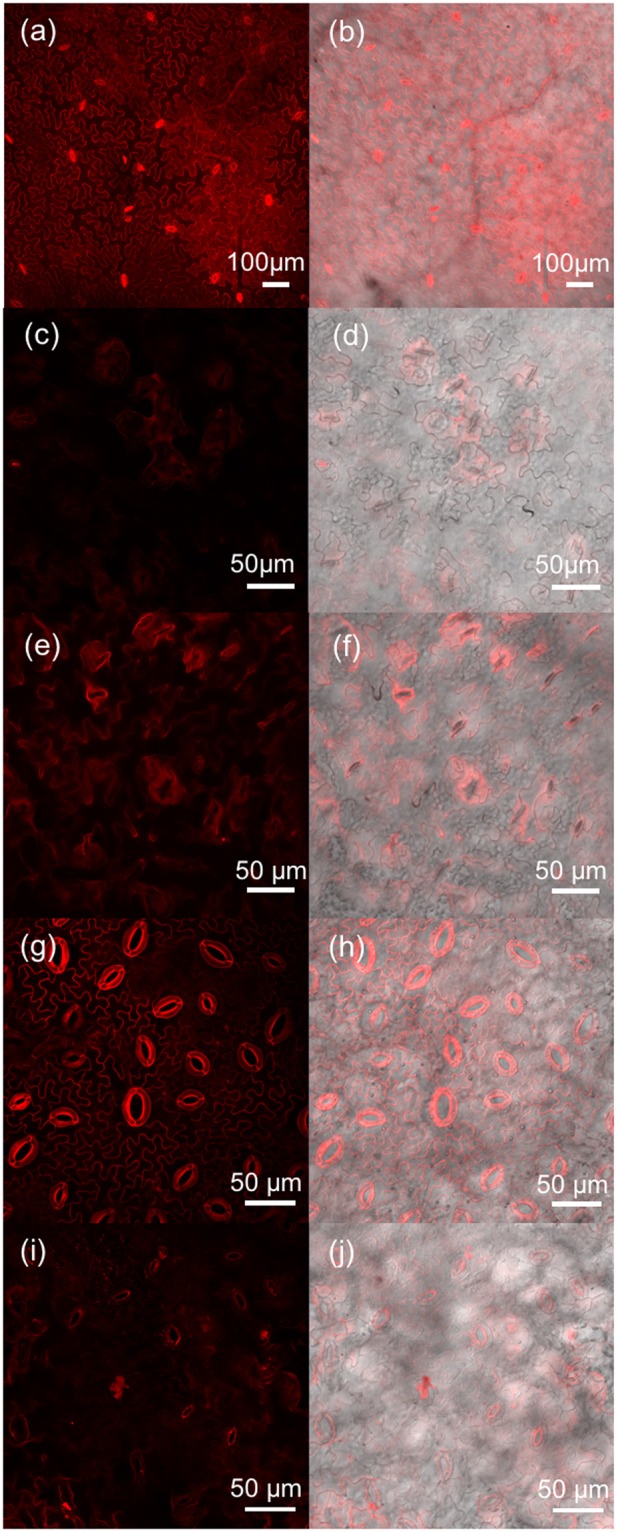
CLSM images of leaf epidermal cells infiltrated with TAMRA-labeled CPPs that showed low cell penetration efficiency. (**a**,**b**) Peptide No. 5 in a *N. benthamiana* leaf. (**c**,**d**) Peptide No. 34 and (**e**,**f**) peptide No. 50 in an *A. thaliana* leaf. (**g**,**h**) Peptide No. 5 (**i**,**j**) and peptide No. 34 in a poplar leaf. (**a**,**c**,**e**,**g**,**i**) TAMRA fluorescence images. (**b**,**d**,**f**,**h**,**j**). Overlay images of TAMRA fluorescence and DIC images.

As 2× ppTG1 (Peptide No. 34), G53-4 (Peptide No. 50), and D-R9 (Peptide No. 5) showed low penetration efficiencies in some species, we further examined the detailed CLSM observations of these CPPs infiltrated into *A. thaliana* and poplar. In the case of *N. benthamiana*, typical signals in nuclei and cytosols were detected by the infiltration of D-R9 (Fig. 13a,b). When *A. thaliana* was infiltrated with D-R9, 2× ppTG1, or G53-4, and when poplar was infiltrated with D-R9 or 2× ppTG1 (Fig. 13c–j), the TAMRA signals localized around stomata including guard cells. When plant leaves are infiltrated with CPPs, CPPs seem to enter the leaves through stomata. The low efficiency CPPs were concentrated around the stomata, whereas the high efficiency CPPs did not accumulate in the guard cells around stomata, but penetrated the epidermal cells evenly. In this study, we infiltrated plant leaves with CPP solutions, and hence the solubility of the CPPs affected their ability to penetrate the epidermis, and CPPs with low solubility tended to enter the leaf through stomata. Thus, the penetration efficiency of CPPs may change if methods other than infiltration are used. We plan to examine the effects of the shape and number of stomata on cell penetration efficiency in a future study.

CPPs are short peptides used more frequently in biomedical applications than in biotechnological and agricultural applications. One example of CPP applications in plant biotechnology is transient modifications of plants using chemicals and enzymes. We previously introduced neomycin phosphotransferase II (NPTII) into apple leaf cells to add transient kanamycin resistance using the fusion peptide system^37^. Moreover, gene delivery systems for plant cells have been developed using CPP as a part of carrier molecules, indicating that CPP can be combined with the other functional molecules and will become versatile tools for gene delivery and transformation of plants without requiring special equipment. In addition to the transformation technology, CPP can be used for chemical probes to detect viral diseases as well as molecular sensors to monitor cellular state in crop plants^38,39^. This is because the chemical probes and molecular sensors in agriculture cannot be introduced into intact plants through cell walls. Thus, CPP are applicable to various plant biotechnology fields with the combination of another functional molecule such as sensor, probe, signal and carrier. However, we lack information regarding the efficiency of specific CPPs in different plant species and tissues. In this study, we characterized the ability of 55 CPPs to penetrate six different species of plant. Based on the current results, although we found several CPPs that functions with specific plant species and tissues, we could not identify “champion” CPP with high cell penetration efficiency across all plant species and cell types. Hence, we concluded that optimal CPPs will need to be determined for each plant type and target tissue. Furthermore, plant tissues need to be optimized for CPP-mediated treatments. Although old BY-2 cells and rice leaf cells did not uptake CPPs efficiently, BY-2 cells at 3 days after subculture and rice callus demonstrated high efficiencies with several CPPs. Perhaps, we need to consider some additives, such as surfactants or chelating agents, to enhance the CPP efficiency. This study is the first case of test for the plant species-specific cell penetration efficiency of CPP library; the results described here should provide a platform for the biological and chemical properties of CPPs related to their cell penetration efficiency in plants, which would improve the utility of CPPs as one of promising efficient tools in plant biotechnology.

### Experimental procedures

#### Plant preparations and growth conditions

Suspension-cultured tobacco (*Nicotiana tabacum*) cv. BY-2 cells were provided by the RIKEN Bio Resource Center. The BY-2 cells were subcultured in Murashige-Skoog medium with 2 mg/L 2,4-D once a week at 26.5 °C in the dark with an orbital shaker (130 rpm). For the study on the growth phase of BY-2 cells, we prepared one-day-old, three-day-old and 7-day-old BY-2 cells from the subculture were used. Based on the cell penetrating efficiency, three-day-old BY-2 cells from the subculture were used for further experiments. Seeds of *Nicotiana benthamiana, Arabidopsis thaliana* (Columbia), and *Solanum lycopersicum* (Moneymaker tomato) were germinated in pots containing a mixture of soil (Pro-Mix, Premier Tech Ltd, Canada) and vermiculite in a ratio of 2:1. *A. thaliana* was grown and incubated under long-day conditions (16 h light/8 h dark) at 21 °C in a plant incubator (Biotron NK System, Japan). *N. benthamiana* was grown and incubated under all-day conditions (24 h light) at 30 °C. Moneymaker tomato was grown and incubated under long-day conditions (16 h light/8 h dark) at 26 °C. The poplar tree, hybrid aspen T89 lines (*Populus tremula* × *tremuloides*), was grown and incubated under short-day conditions (8 h light/16 h dark) at 22 °C, according to a previous report^40^. Rice seeds (*Oryza sativa*, cv. Nipponbare) were germinated under long-day conditions (16 h light/8 h dark) at 30 °C in Hoagland solution^41^. For rice callus induction, dehusked mature seeds were sterilized and inoculated into N6D medium at 30 °C^42^. After 4 weeks, the proliferated calli (1 mm diameter) derived from the scutella were used for CPP assay.

#### Peptide synthesis

Fifty-five CPPs were synthesized using standard 9-fluorenylmethoxycarbonyl (Fmoc) solid phase peptide synthesis^43^ and were included the Lys modified with TAMRA (approximate excitation/emission maxima 546/579 nm) via Lys side chain amine at the C-terminus (Fig. S14). The TAMRA-labeled CPPs were purified using high-performance liquid chromatography (HPLC), and the resultant purities of these peptides were characterized by HPLC with a Gemini-NX 5μ C18 110 A column (Phenomenex Inc., CA) at 25 °C (Fig. S15). The mobile phase comprised 10–67% CH_3_CN containing 0.1% TFA. The flow rate was 1.0 mL/min. The molecular weights were confirmed by matrix-assisted laser desorption/ionization-time-of-flight (MALDI-TOF) mass spectrometry. IX (Peptide No. 18), 2× ppTG1 (Peptide No. 34) and G53-4 (Peptide No. 50) were dissolved into DMSO before adding into culture media or MilliQ water for infiltrations (final DMSO concentration: 2.5%), because of their poor water solubility.

#### Circular dichroism spectroscopy

The secondary structure of the CPPs dissolved in MilliQ water were characterized by circular dichroism (CD, JASCO J-820, JASCO, Tokyo, Japan). The CPP concentration was 10 µM. Background scans were obtained for MilliQ water. Measurements were made using a quartz cuvette with a 0.1 cm path length. Each spectrum represented the average of ten scans from 190 to 240 nm with a 1 nm resolution obtained at 200 nm/min with a bandwidth of 1 nm. The secondary structure content of each peptide was calculated by the DichroWeb online CD analysis server using SELCON3 algorithms in combination with reference dataset 4 optimized for data in the range of 190 to 240 nm in accordance with two previous studies^44,45^.

#### Cell penetration efficiency

The penetration efficiency of CPPs into BY-2 suspension cells and young true leaves of *N. benthamiana, A. thaliana*, Moneymaker tomato, *O. sativa* Nipponbare, and poplar, and *O. sativa* callus was determined using confocal laser scanning microscopy (CLSM, ZeissLSM 700, Carl Zeiss, Oberkochen, Germany). The intracellular localization of TAMRA-labeled CPP was observed using excitation at 555 nm for the detection of TAMRA fluorescence. The total number of cells was counted using differential interfering contrast images. The efficiency was determined as follows:

$${\rm{Cell}}\,{\rm{penetration}}\,{\rm{efficiency}}\,( \% )=({\rm{the}}\,{\rm{number}}\,{\rm{of}}\,{\rm{stained}}\,{\rm{cells}})/({\rm{the}}\,{\rm{number}}\,{\rm{of}}\,{\rm{total}}\,{\rm{cells}})\times {\rm{100}}$$

Various BY-2 cell concentrations (230 to >1000 cells/µL) of BY-2 cells were tested to determine the optimal cell concentration. CPP concentrations between 20 and 150 µg/mL were also prepared and tested for optimal penetration condition in BY-2 cells. CPPs were incubated with BY-2 suspension for 3 h. For the young true leaves, CPPs were infiltrated and incubated for 3 h. The infiltration method was summarized in a previous movie^16^. The cell penetration efficiency into the true leaves was calculated by counting the fully-stained and non-stained epidermal cells. Guard cells were not considered for this calculation because guard cells are readily stained and differ from the epidermal cells in terms of peptide penetration efficiency. For rice callus, callus of 50 mg was immersed in 200 µL of 0.1 mg/mL CPP solution, subsequently, depressurized it at 0.5 atm for 1 min. After the treatment, we incubated the callus on the medium for 3 hours at 30 °C. The cell penetration efficiency into the callus was calculated by counting the fully-stained and non-stained cells.

#### Evans blue assay

The cell viability against CPPs were evaluated by the Evans blue (EB) assay^46^. For the assay, CPP (final concentration: 0.1 mg/mL) were incubated with BY-2 cells (OD_600_: 0.5) for 1 hour at 26.5 °C under dark condition. The incubated BY-2 cells were washed with Milli-Q water and mixed with 50 µg/mL Evans blue for 10 min. The stained BY-2 cells were washed with Milli-Q water and were treated with methanol/SDS solution for 2 hours. The lysates were centrifuged and the supernatants were measured in OD600. For a positive control (100% dead cells), BY-2 cells were treated 70% ethanol for 60 min to kill completely then 50 µg/mL Evans blue were added for 10 min and washed 3 times.

#### Statistical analysis

Tukey’s Honestly Significant Difference (HSD) test was used in conjunction with one-way analysis of variance (ANOVA) for single step multiple comparisons to analyze the results using IBM SPSS Statistics for Macintosh v. 22 (IBM, Armonk, NY). Differences between two means were considered statistically significant at *P* < 0.05. The mean data are labeled with different letters (*x*, *y*, and *z*). Data in experiments are expressed as the means ± standard deviation (n = 3).

### Data availability statement

All data generated or analyzed during this study are included in this published article (and its Supplementary Information file).

## Electronic supplementary material

Supplementary information
