# Supplementary material for: Library screening of cell-penetrating peptide for BY-2 cells, leaves of Arabidopsis, tobacco, tomato, poplar, and rice callus
Source: Sci Rep. 2018 Jul 20;8:10966. doi: 10.1038/s41598-018-29298-6 (PMC6054692; doi:10.1038/s41598-018-29298-6)
Supplement: Supplementary file 1 — Supplementary information [file 41598_2018_29298_MOESM1_ESM.pdf]

Supporting Information

Library screening of cell-penetrating peptide for BY-2 cells, leaves of Arabidopsis, tobacco, tomato, poplar, and rice callus

Keiji Numata[1]*, Yoko Horii[1], Kazusato Oikawa[1], Yu Miyagi[1], Taku Demura[1,2] and Misato Ohtani[1,2]

[1]Biomacromolecules Research Team, RIKEN Center for Sustainable Resource Science, 2-1 Hirosawa, Wako-shi, Saitama 351-0198, Japan.

[2]Graduate School of Biological Sciences, Nara Institute of Science and Technology, Ikoma, Nara 630-0192, Japan.

Figure S1. CD spectra of 55 types of CPP.
Figure S2. Effects of BY-2 growth phase on the cell penetrating efficiency of BP100.
Figure S3. CLSM images of BY-2 cells incubated with 55 types of CPP.
Figure S4. BY-2 cells before and after incubation with TAMRA alone.
Figure S5. BY-2 cell death rate of 55 types of CPPs.
Figure S6. Relationship between the cell penetrating efficiencies into BY-2 cells and *Nicotiana benthamiana* leaves and the numbers of Lys and Arg in the amino acid sequences of CPPs.
Figure S7. Summary of CLSM images of *N. benthamiana* leaf epidermal cells after infiltration of 55 types of CPP.
Figure S8. CLSM images of *N. benthamiana* leaf epidermal cells after infiltration of 55 types of CPP.
Figure S9. CLSM images of *N. benthamiana* cotyledons epidermal cells after infiltration of BP100.
Figure S10. CLSM images of *A. thaliana* leaf epidermal cells after infiltration of three groups of CPPs.
Figure S11. CLSM images of Moneymaker tomato leaf epidermal cells after infiltration of three groups of CPPs.
Figure S12. CLSM images of poplar leaf epidermal cells after infiltration of three groups of CPPs.
Figure S13. CLSM images of rice (*O. sativa*) callus after infiltration of 55 CPPs.
Figure S14. Reaction scheme to synthesize TAMRA-labeled CPP.
Figure S15. HPLC profiles of the peptides used in this study.

Table S1. Cell penetrating efficiency into various plant types of three groups of CPP.

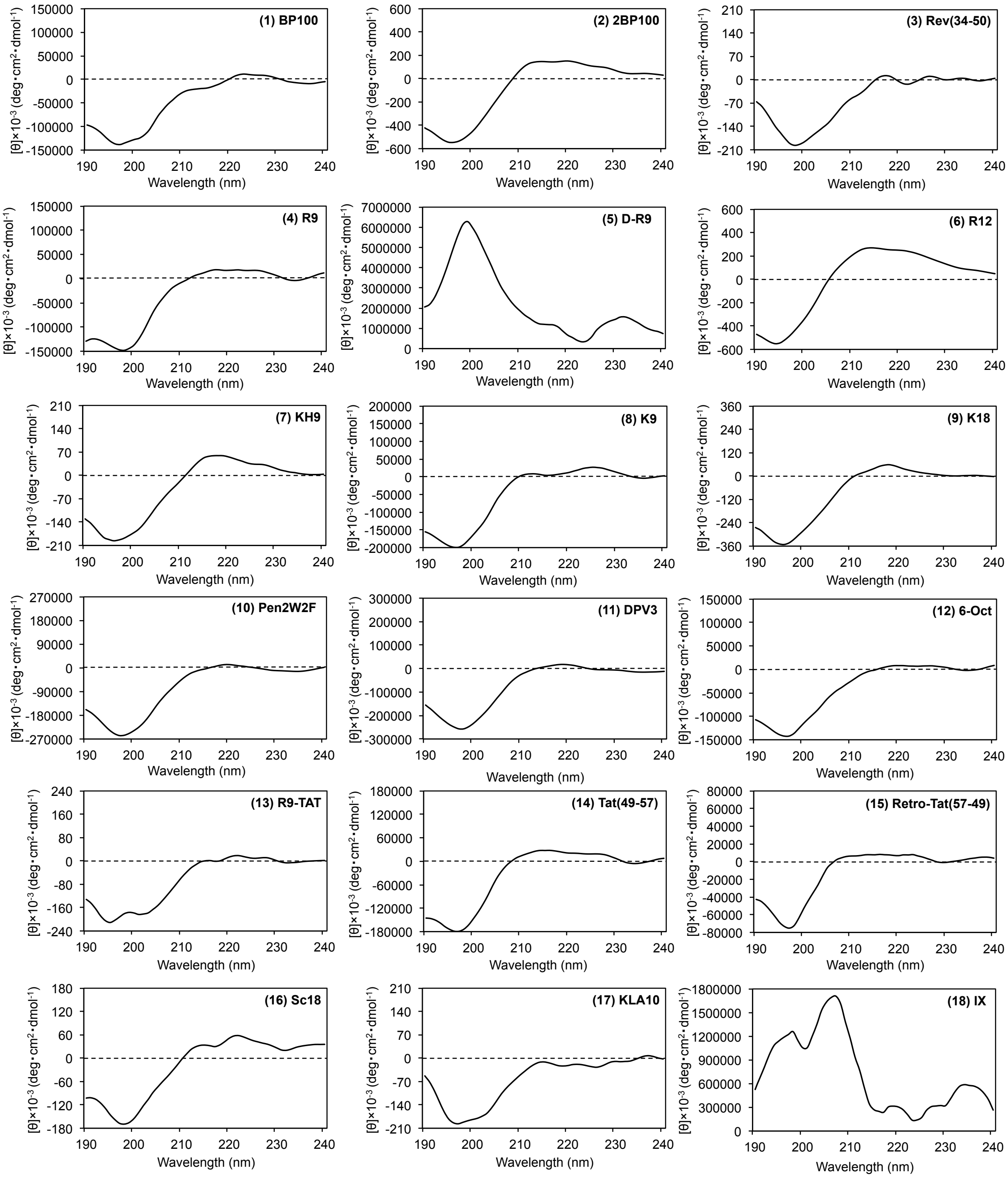
(1) BP100
(2) 2BP100
(3) Rev(34-50)
(4) R9
(5) D-R9
(6) R12
(7) KH9
(8) K9
(9) K18
(10) Pen2W2F
(11) DPV3
(12) 6-Oct
(13) R9-TAT
(14) Tat(49-57)
(15) Retro-Tat(57-49)
(16) Sc18
(17) KLA10
(18) IX
[θ]×10^{-3} (deg•cm^2•dmol^{-1})
Wavelength (nm)

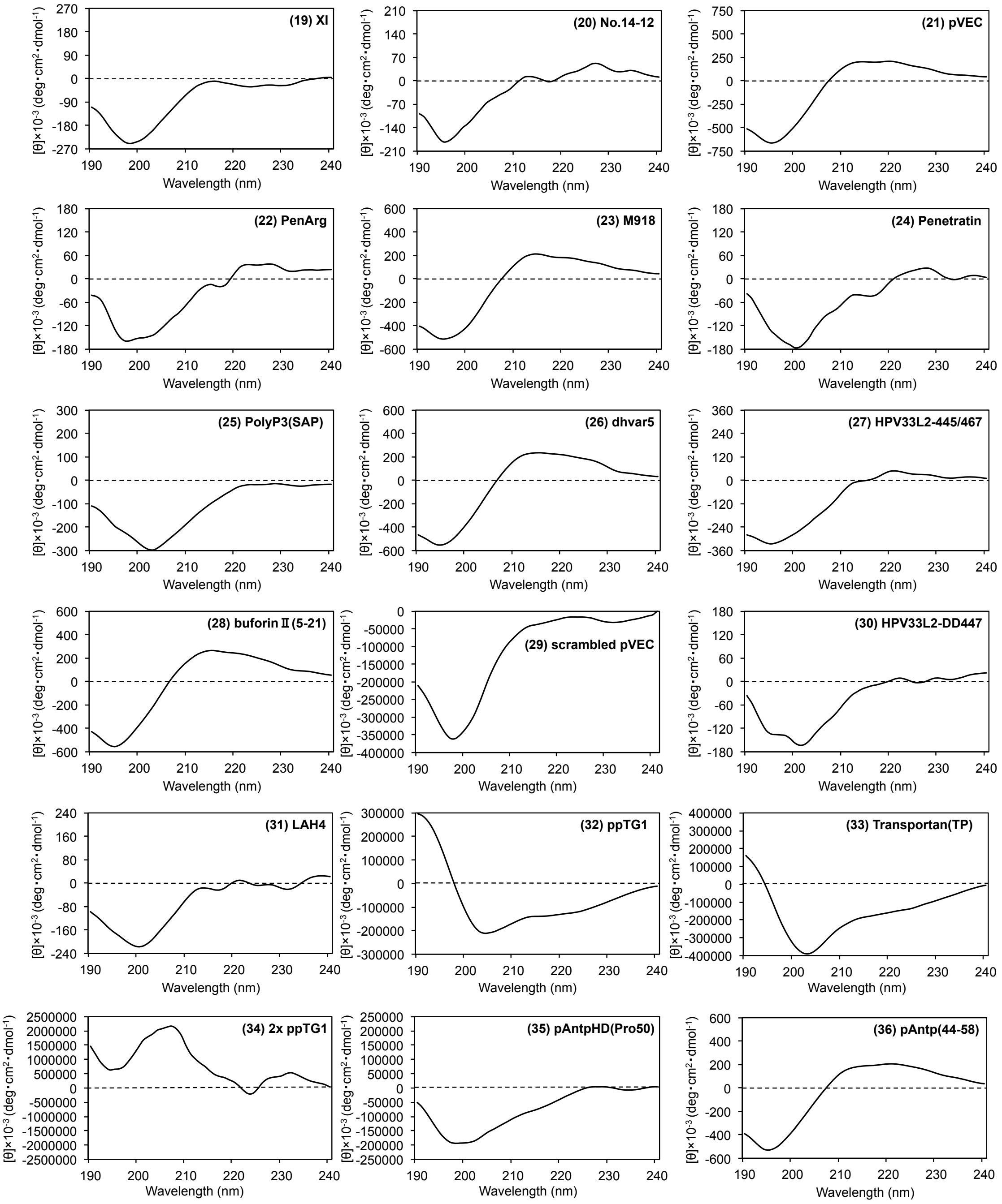
(19) XI
(20) No.14-12
(21) pVEC
(22) PenArg
(23) M918
(24) Penetratin
(25) PolyP3(SAP)
(26) dhvar5
(27) HPV33L2-445/467
(28) buforin Ⅱ (5-21)
(29) scrambled pVEC
(30) HPV33L2-DD447
(31) LAH4
(32) ppTG1
(33) Transportan(TP)
(34) 2x ppTG1
(35) pAntpHD(Pro50)
(36) pAntp(44-58)
[θ]×10⁻³ (deg•cm²•dmol⁻¹)
Wavelength (nm)

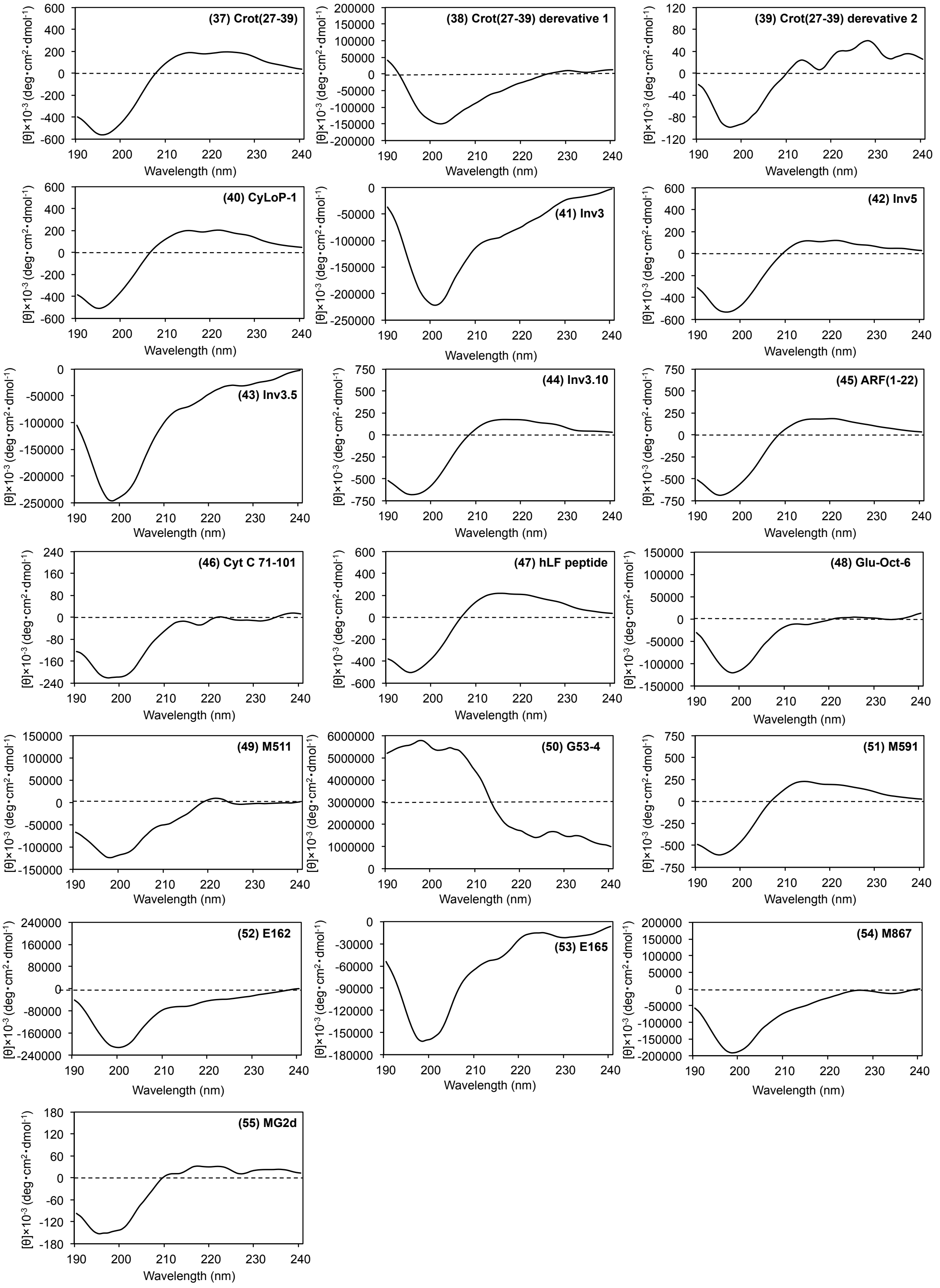
(37) Crot(27-39)
(38) Crot(27-39) derevative 1
(39) Crot(27-39) derevative 2
(40) CyLoP-1
(41) Inv3
(42) Inv5
(43) Inv3.5
(44) Inv3.10
(45) ARF(1-22)
(46) Cyt C 71-101
(47) hLF peptide
(48) Glu-Oct-6
(49) M511
(50) G53-4
(51) M591
(52) E162
(53) E165
(54) M867
(55) MG2d
[θ]×10-3 (deg•cm2•dmol-1)
Wavelength (nm)

Figure S1. CD spectra of 55 types of CPP. The type of CPP is shown by peptide number and name as listed in Table 1.

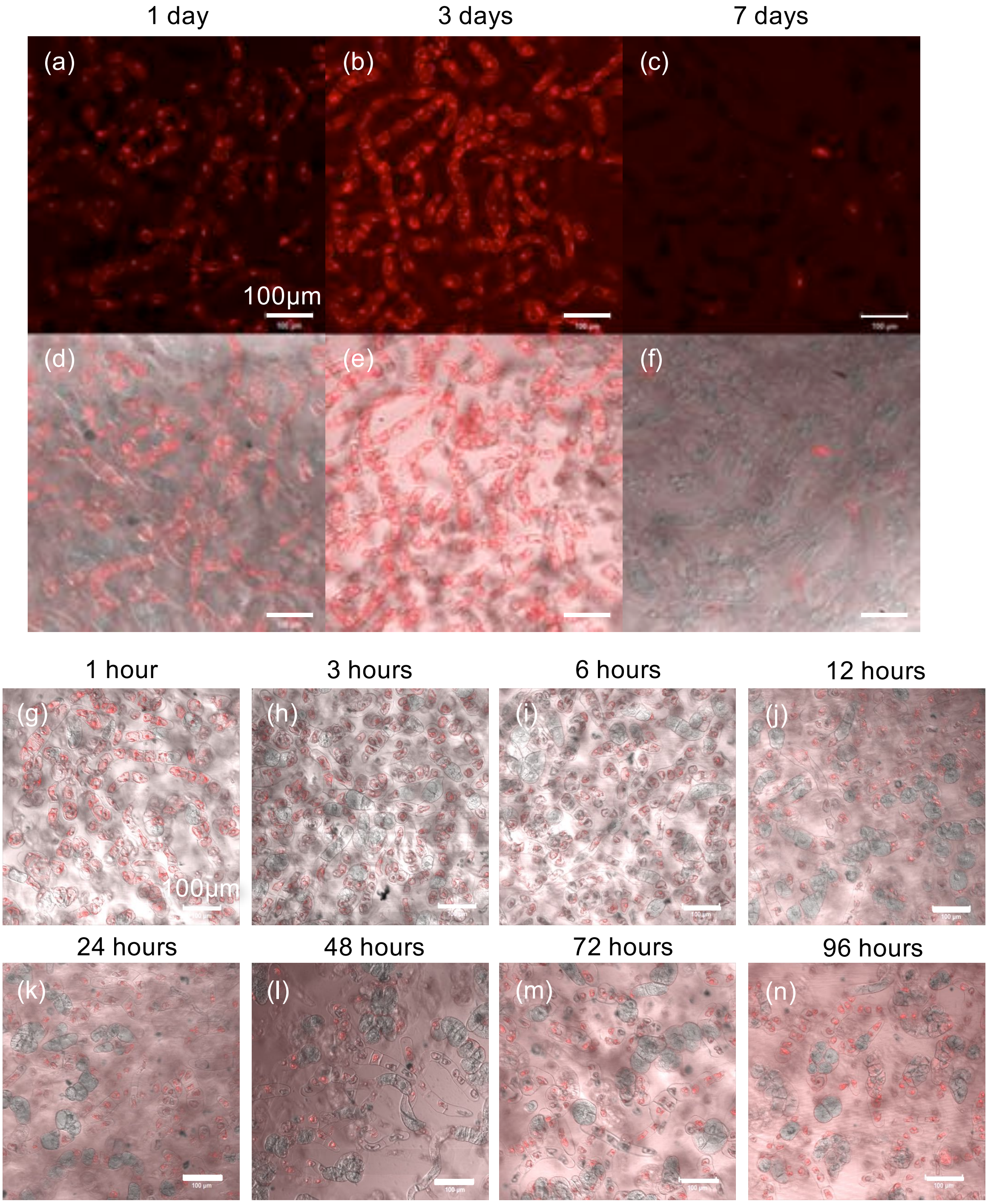

Figure S2. Effects of BY-2 growth phase on the cell penetrating efficiency of BP100 (a-f). BY-2 cells at 1-day (a,d), 3-day (b,e) and 7-day (c,f) after subculture were incubated with TAMRA-labelled BP100 for 3 h at 26ºC. (a-c) TAMRA fluorescence image and (d-

f) overlay image generated by CLSM. Scale bar, 100 µm. Time course CLSM observations of B Y-2 cells incubated with TAMRA-labelled BP100 (g-n).

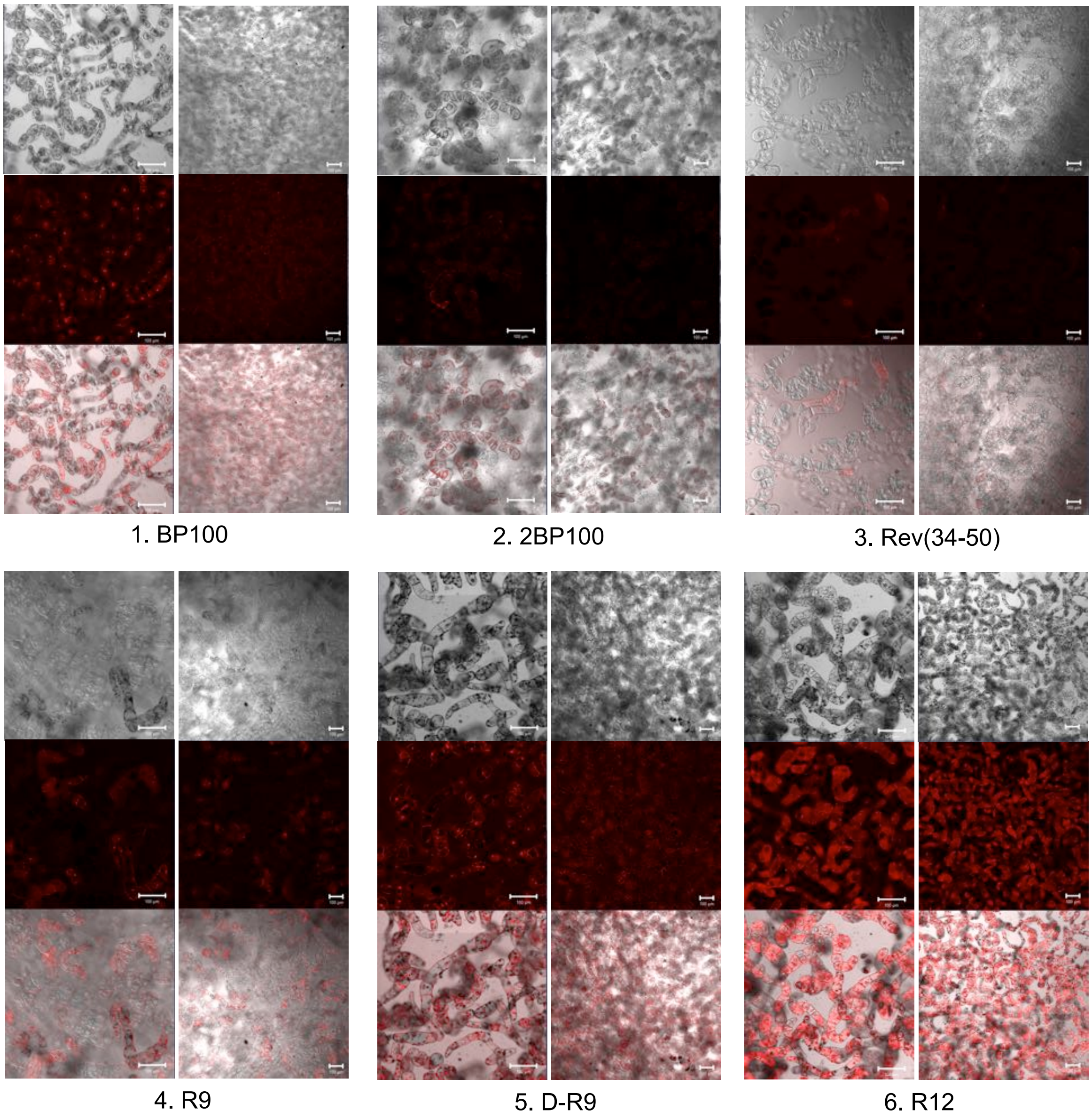

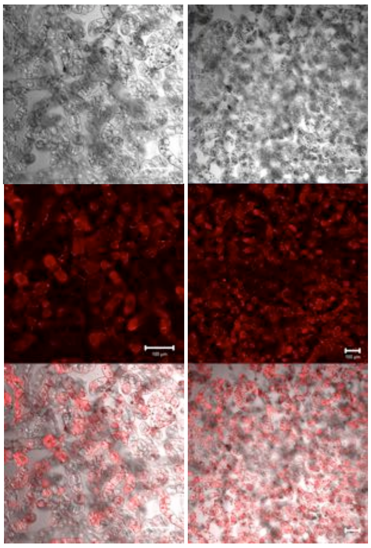

7. KH9

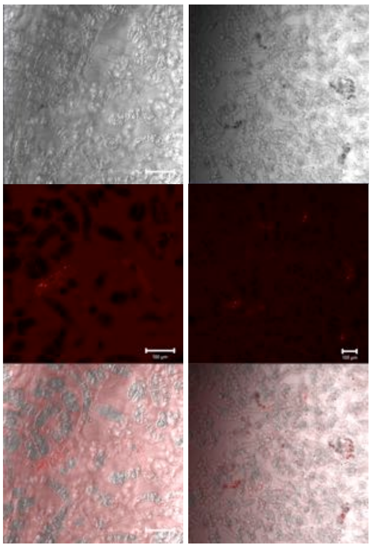

8. K9

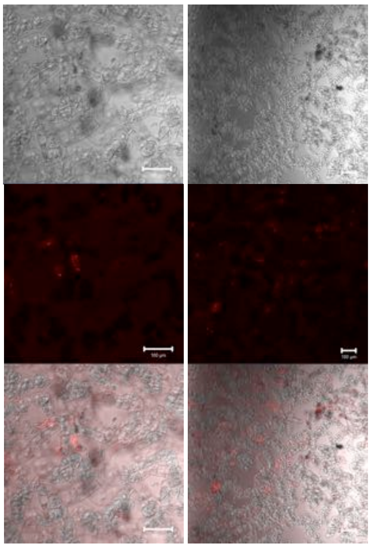

9. K18

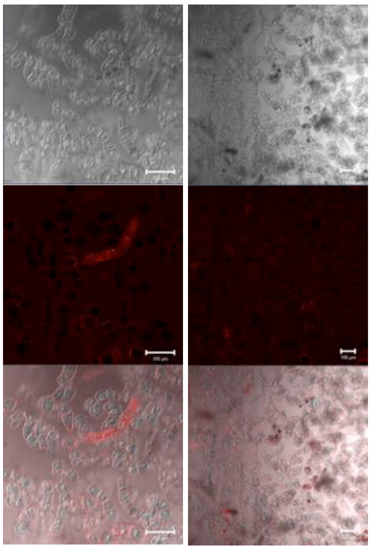

10. Pen2W2F

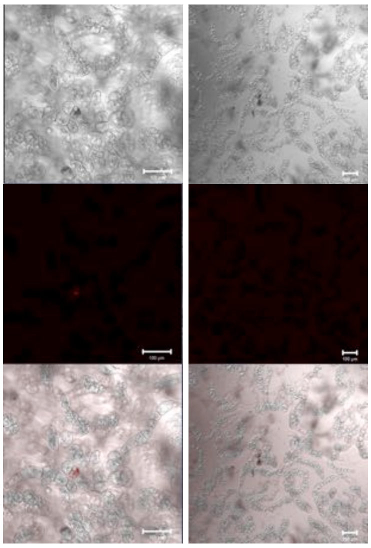

11. DPV3

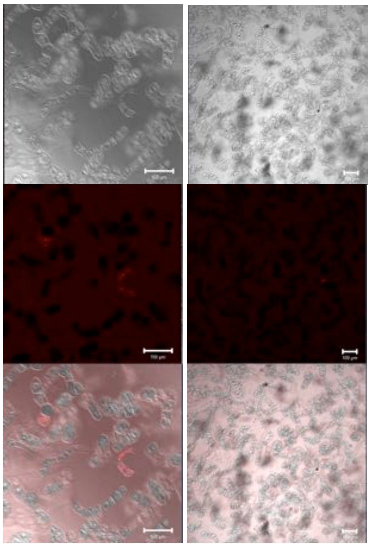

12. 6-Oct

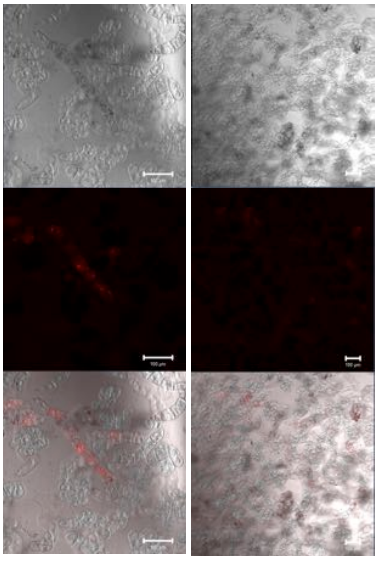

13. R9-TAT

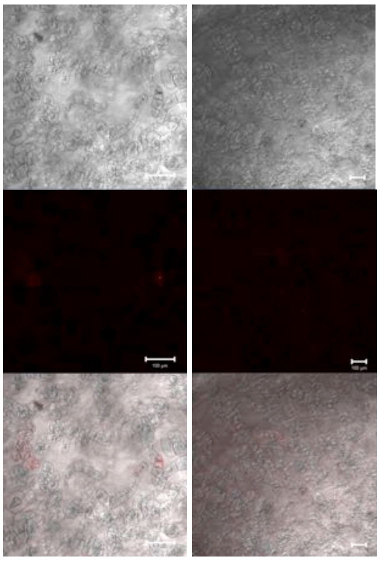

14. Tat(49-57)

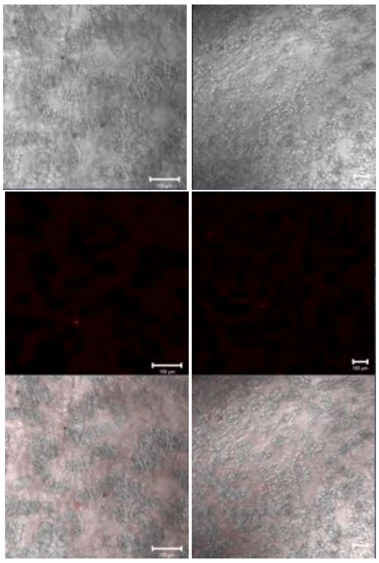

15. RetroTat(57-49)

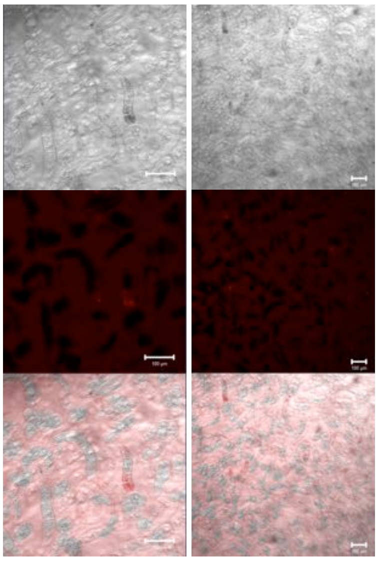

16. K18

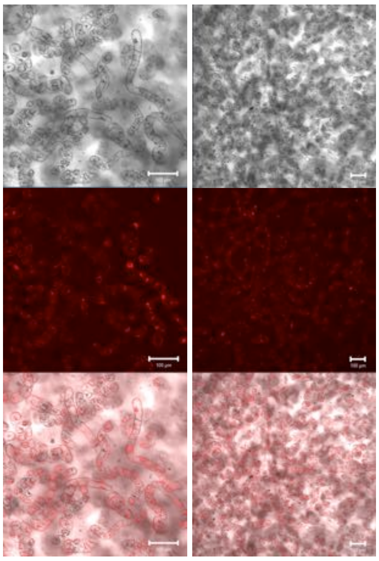

17. KLA10

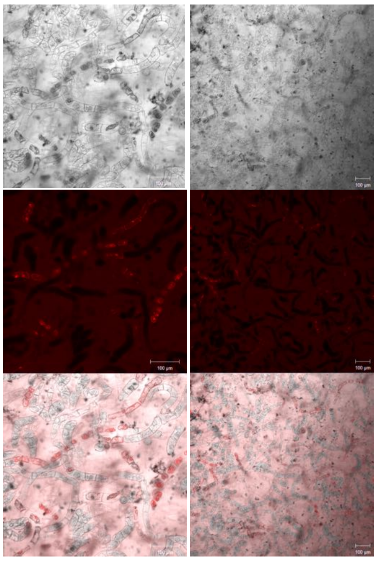

18. IX

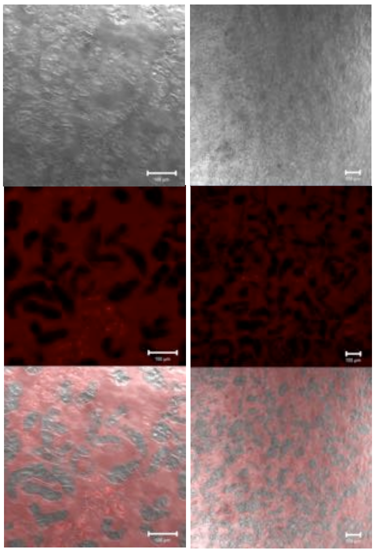

19. XI

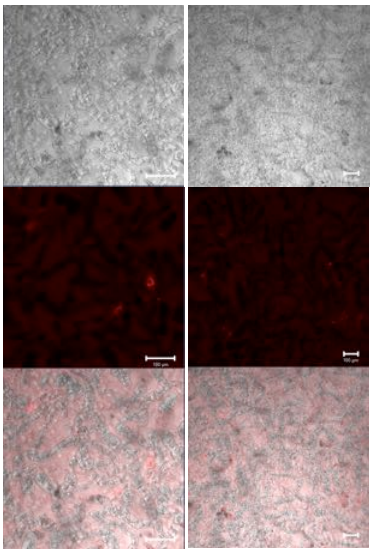

20. No.14-12

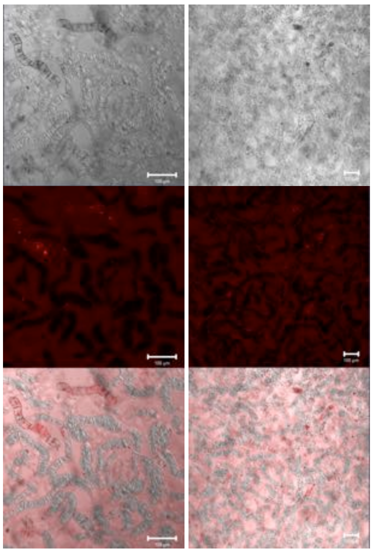

21. pVEC

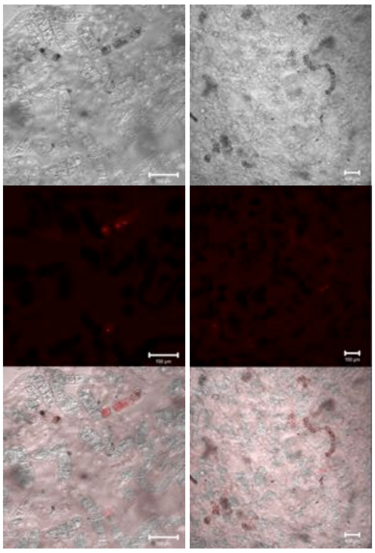

22. PenArg

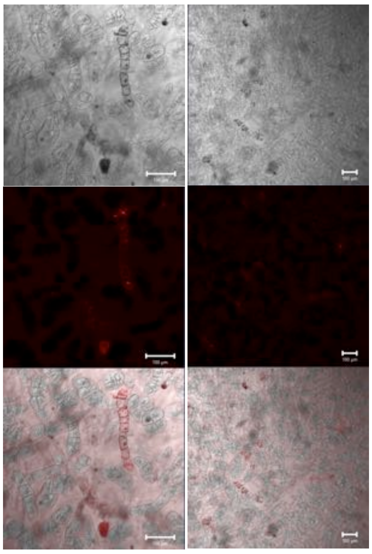

23. M918

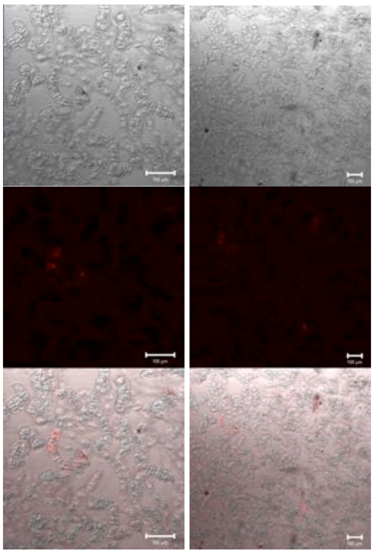

24. Penetratin

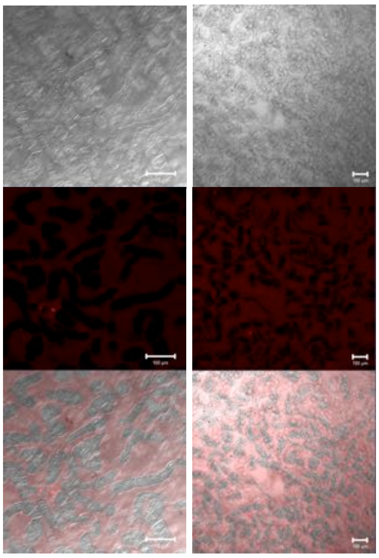

25. PolyP3(SAP)

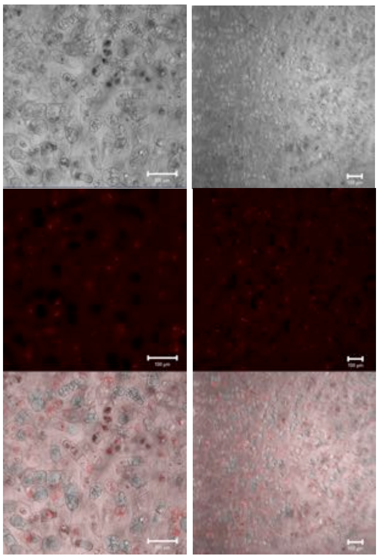

26. dhvar5

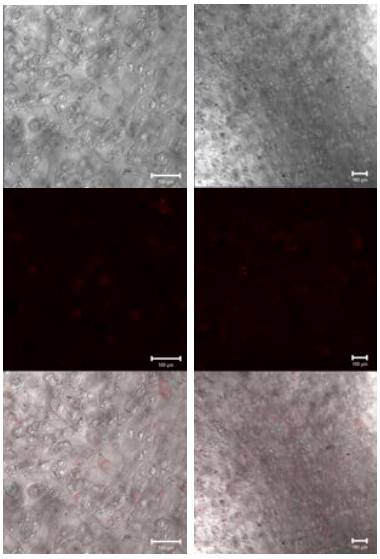

27.HPV33L2-445/467

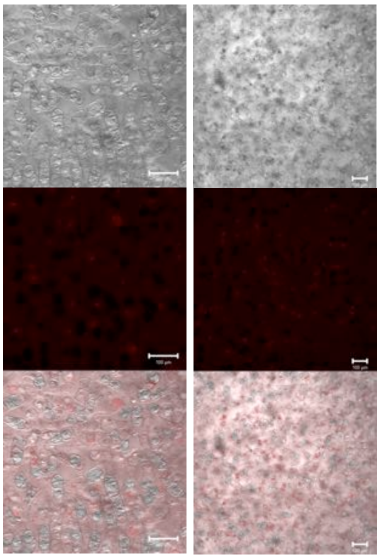

28. Buforin Ⅱ (5-21)

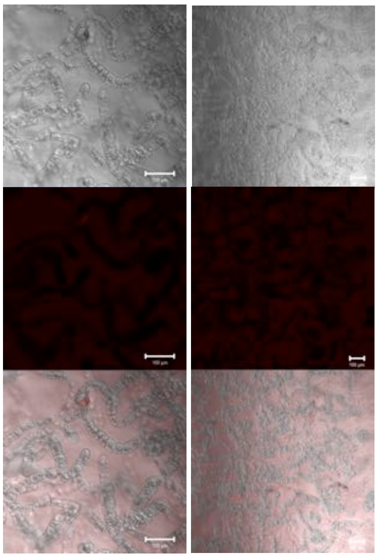

29. Scrambled pVEC

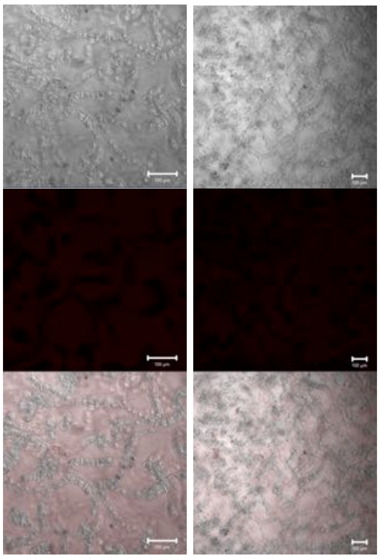

30. HPV33L2-DD447

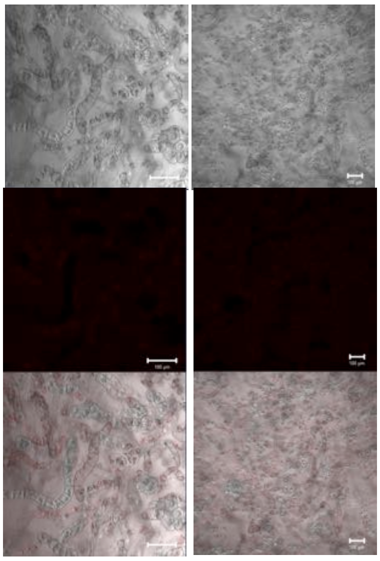

31. LAH4

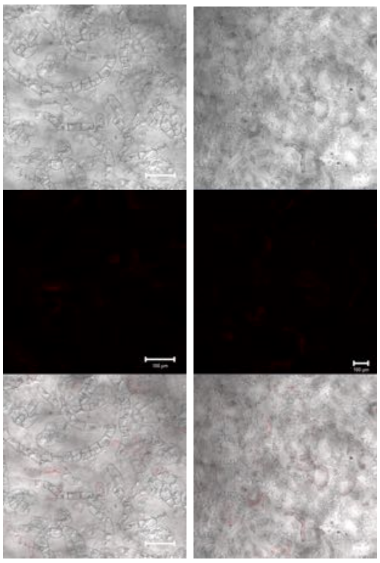

32. ppTG1

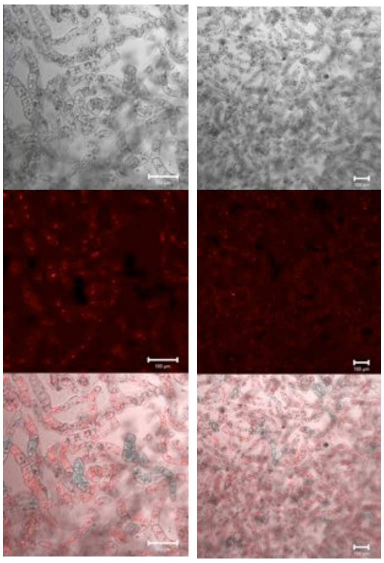

33. transportan(TP)

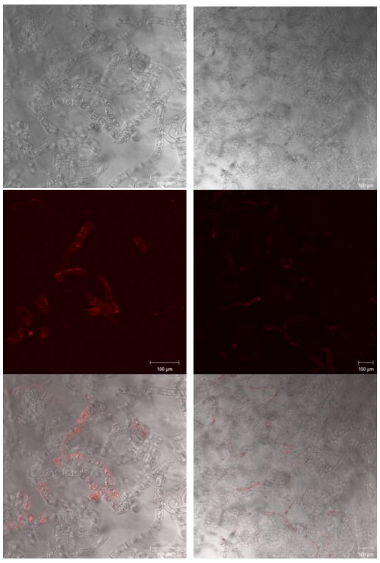

34. 2x ppTG1

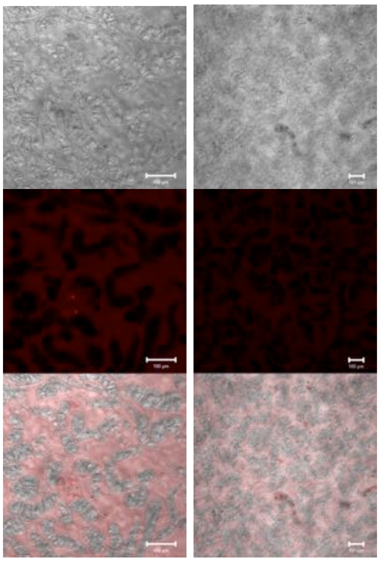

35. pAntpHD(Pro50)

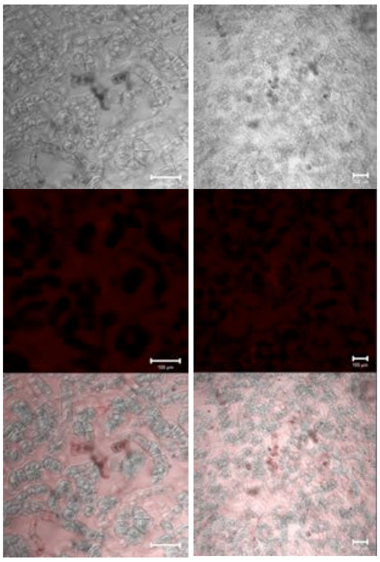

36. pAntp(44-58)

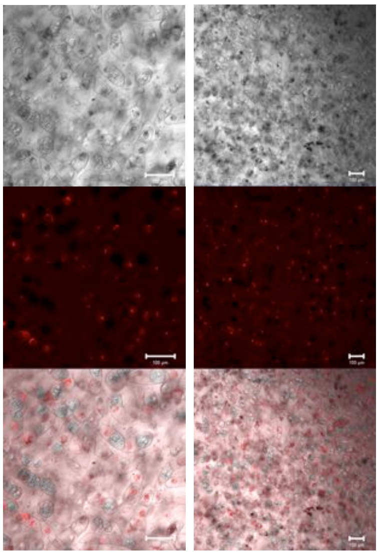

37. Crot(27-39)

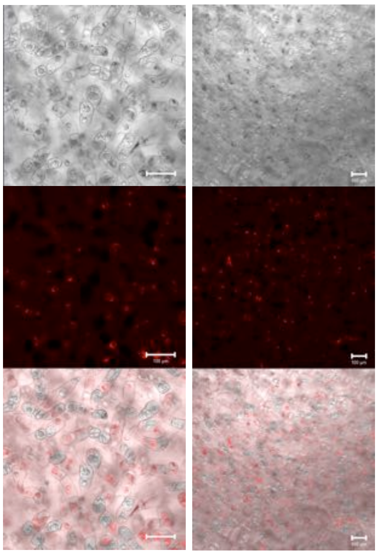

38. Crot(27-39) derevative1

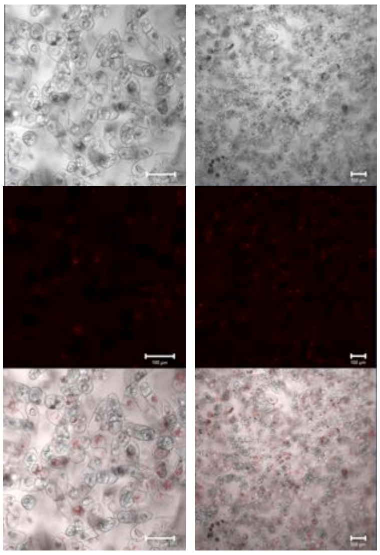

39. Crot(27-39) derevative2

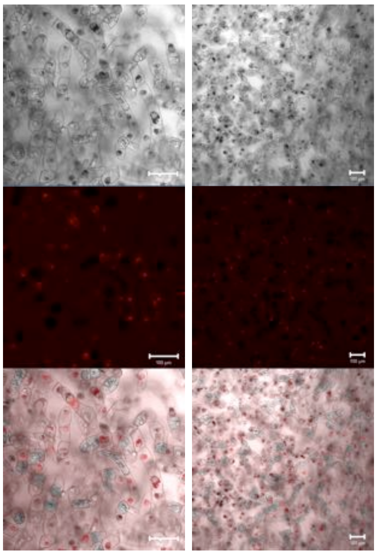

40. CyLoP-1

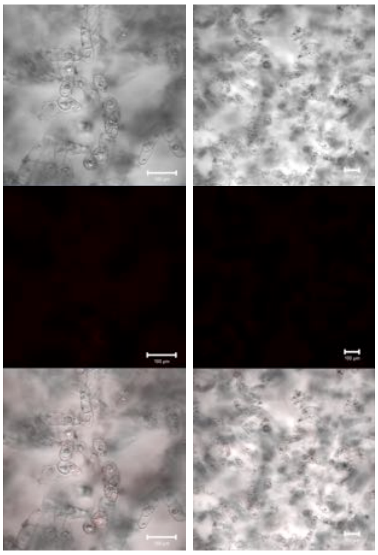

41. Inv3

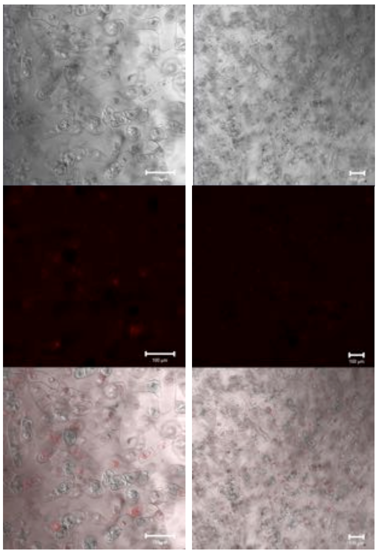

42. Inv5

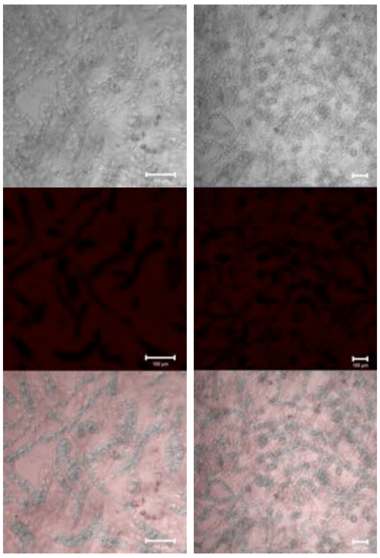

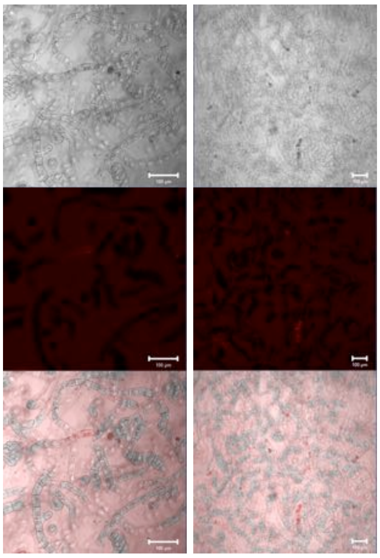

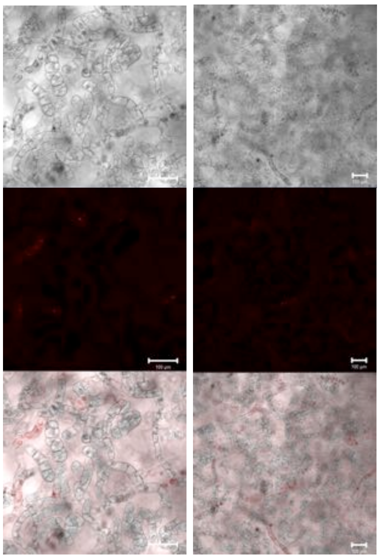

43. Inv3.5

44. Inv3.10

45. ARF(1-22)

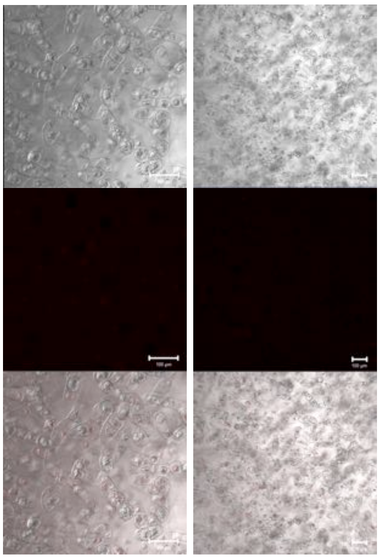

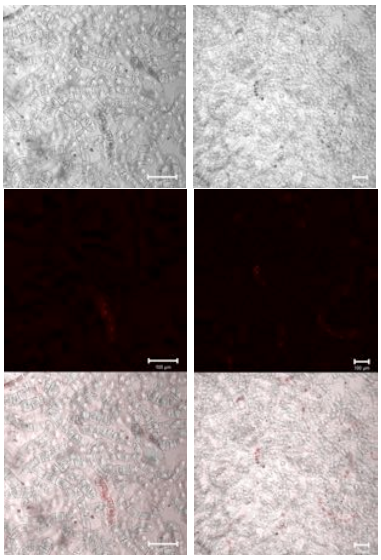

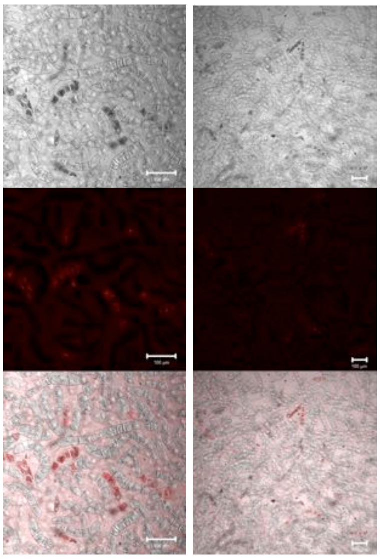

46. Cyt C 71-101

47. hLF peptide

48. Glu-Oct-6

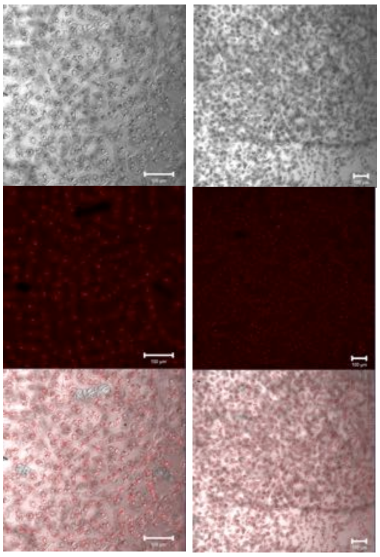

49. M511

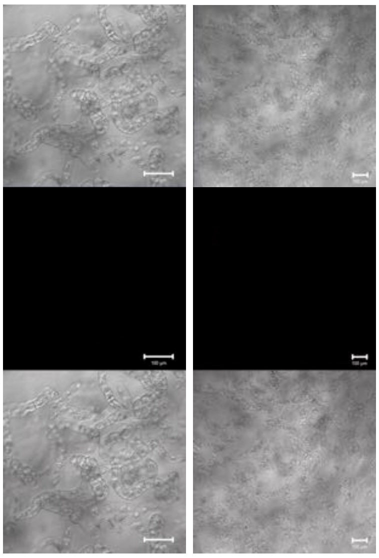

50. G53-4

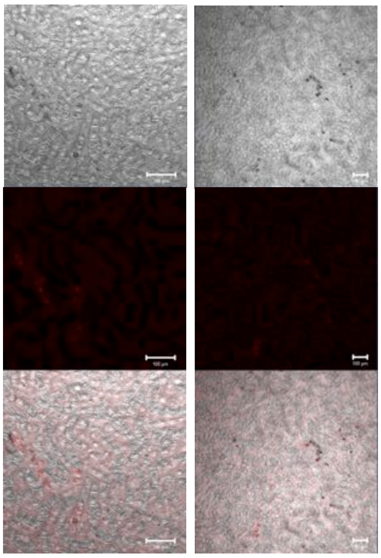

51. M591

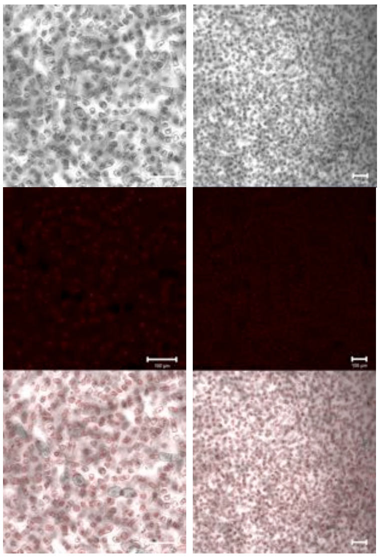

52. E162

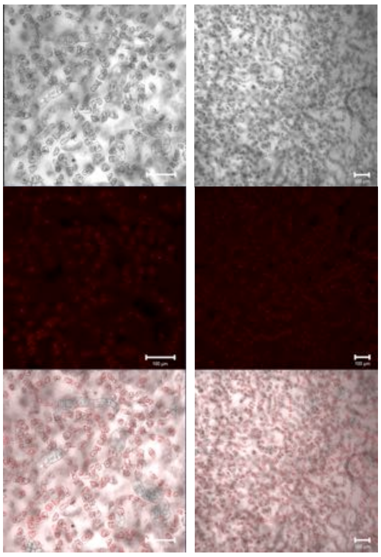

53. E165

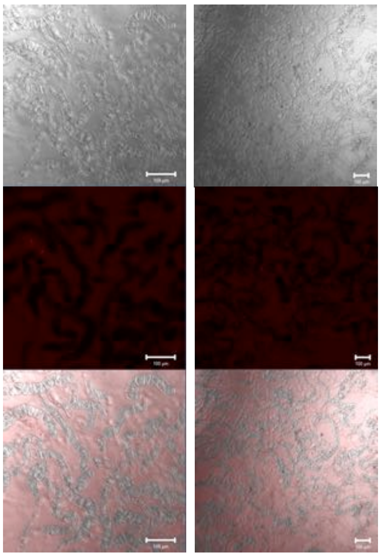

54. M867

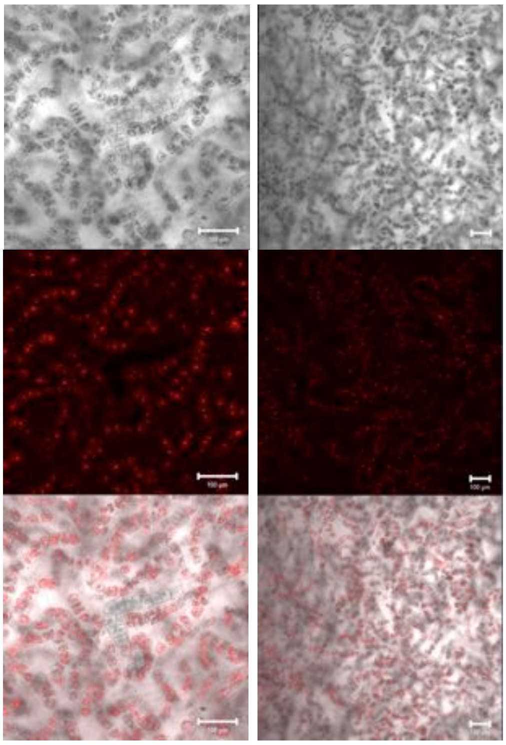

55. MG2d

Figure S3. CLSM images of BY-2 cells incubated with 55 types of CPP for 3 hours at 26ºC. Top, middle and bottom images are DIC, TAMRA fluorescence and overlay images, respectively. The type of CPP is shown by peptide number and name as listed in Table 1. The peptide concentration was 100 μg/mL. The cell density was approximately 380 cells/μL.

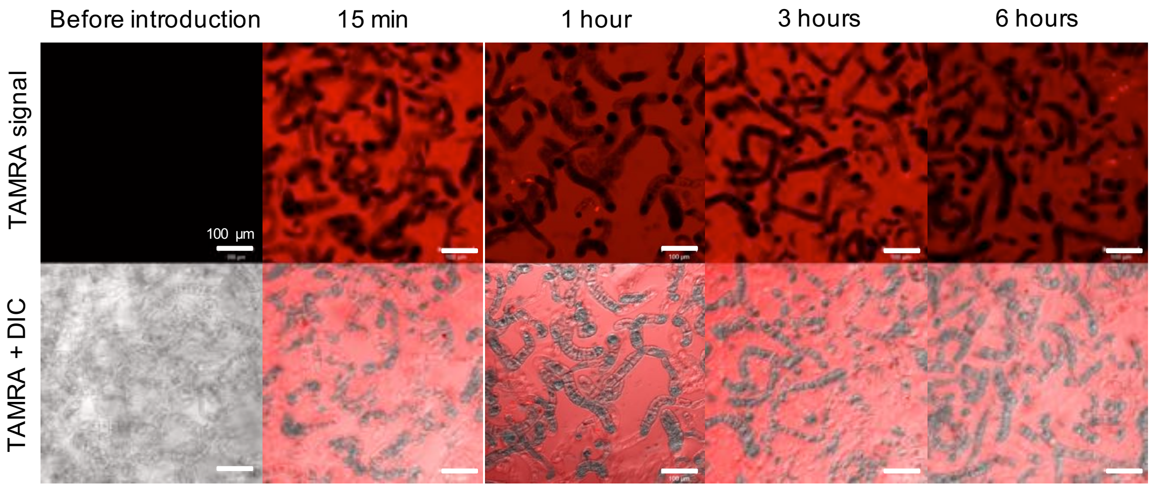

Figure S4. BY-2 cells before and after incubation with TAMRA alone for 15 min, 1 h, 3 h and 6 h at 26ºC. BY-2 cells at 3 days after subculture were used. The top and bottom images are TAMRA fluorescence image and overlay image of TAMRA signal and DIC image generated by CLSM. Scale bar, 100 µm.

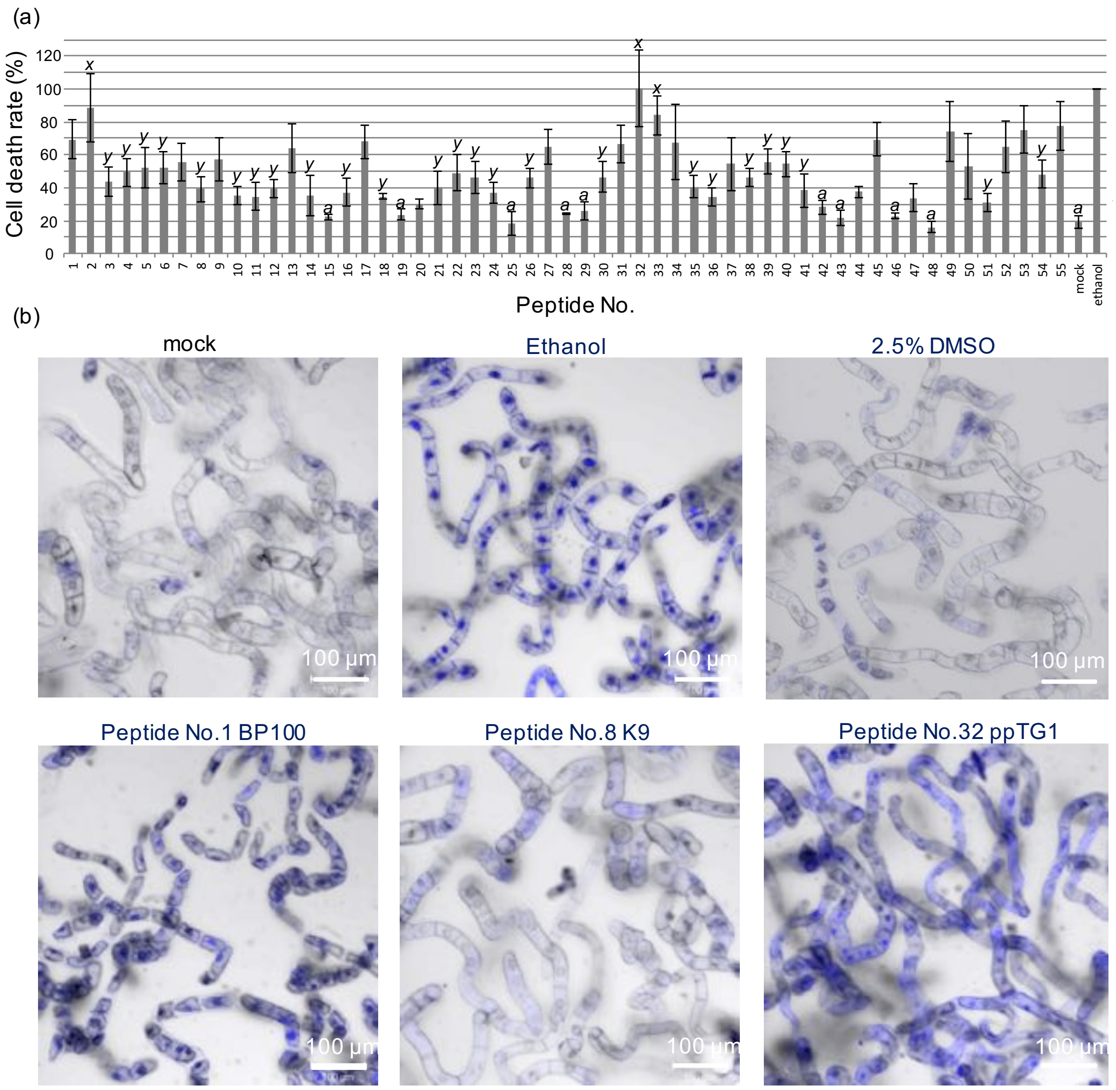

Figure S5. Evans blue assays with BY-2 cells. (a) BY-2 cell death rate of 55 types of CPPs. The type of CPP is shown as peptide number and is listed in Table 1. BY-2 cell was evaluated after a 3-h incubation at 26ºC. For cell death rate, the peptide concentration and cell density were 300 µg/mL and approximately 380 cells/µL, respectively. “Mock” indicates the result with BY-2 cell without CPP (live cells). “Ethanol” indicates the results of ethanol-treated BY-2 cell (dead cells). All data are expressed as the means ± S.D. from triplicate tests. The mean data labeled with different letters (*x* and *y*) are significantly different (Tukey’s HSD test, $P < 0.05$). The mean data labeled with *a* are not significantly different from the mean value of the live cell. (b) CLSM overlay images of Evans Blue signals and DIC of BY-2 cells. Dead cells are indicated by blue color due to the Evans Blue staining. Non-stained cells are live cells.

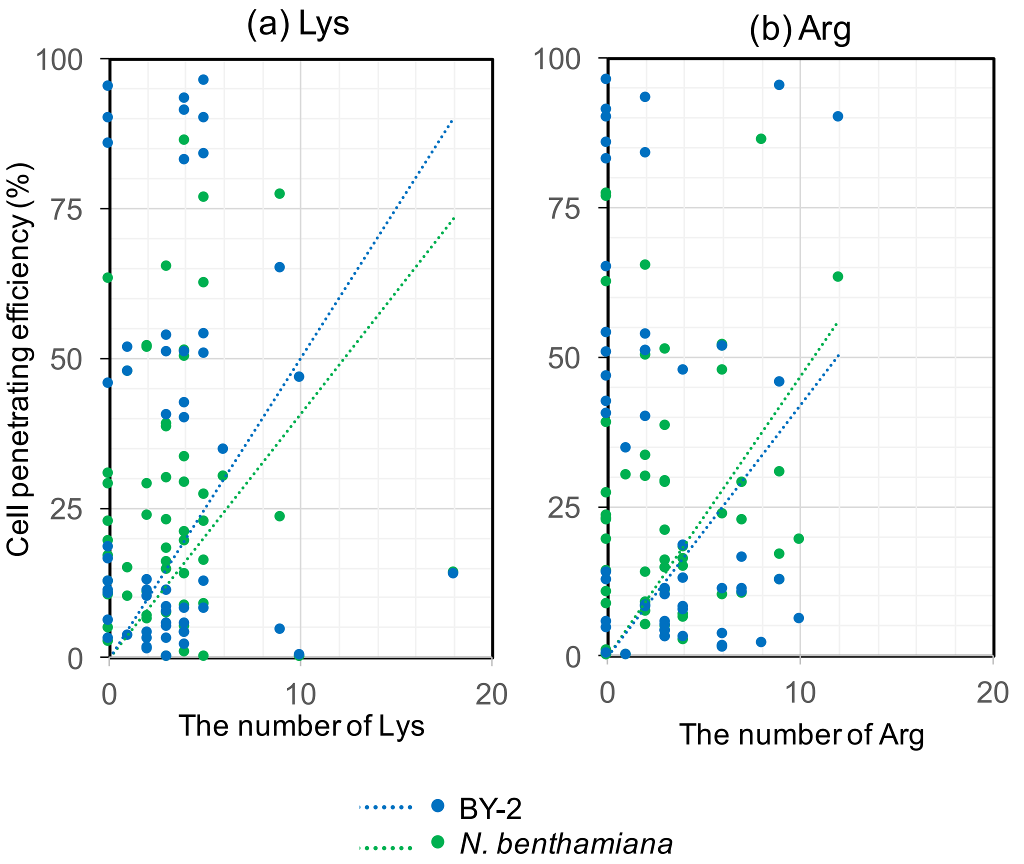

Figure S6. Relationship between the cell penetrating efficiencies into BY-2 cells and *N. benthamiana* leaves and the numbers of Lys (a) and Arg (b) in the amino acid sequences of CPPs. The broken lines are fitting curves using linear least squares analysis through the origin.

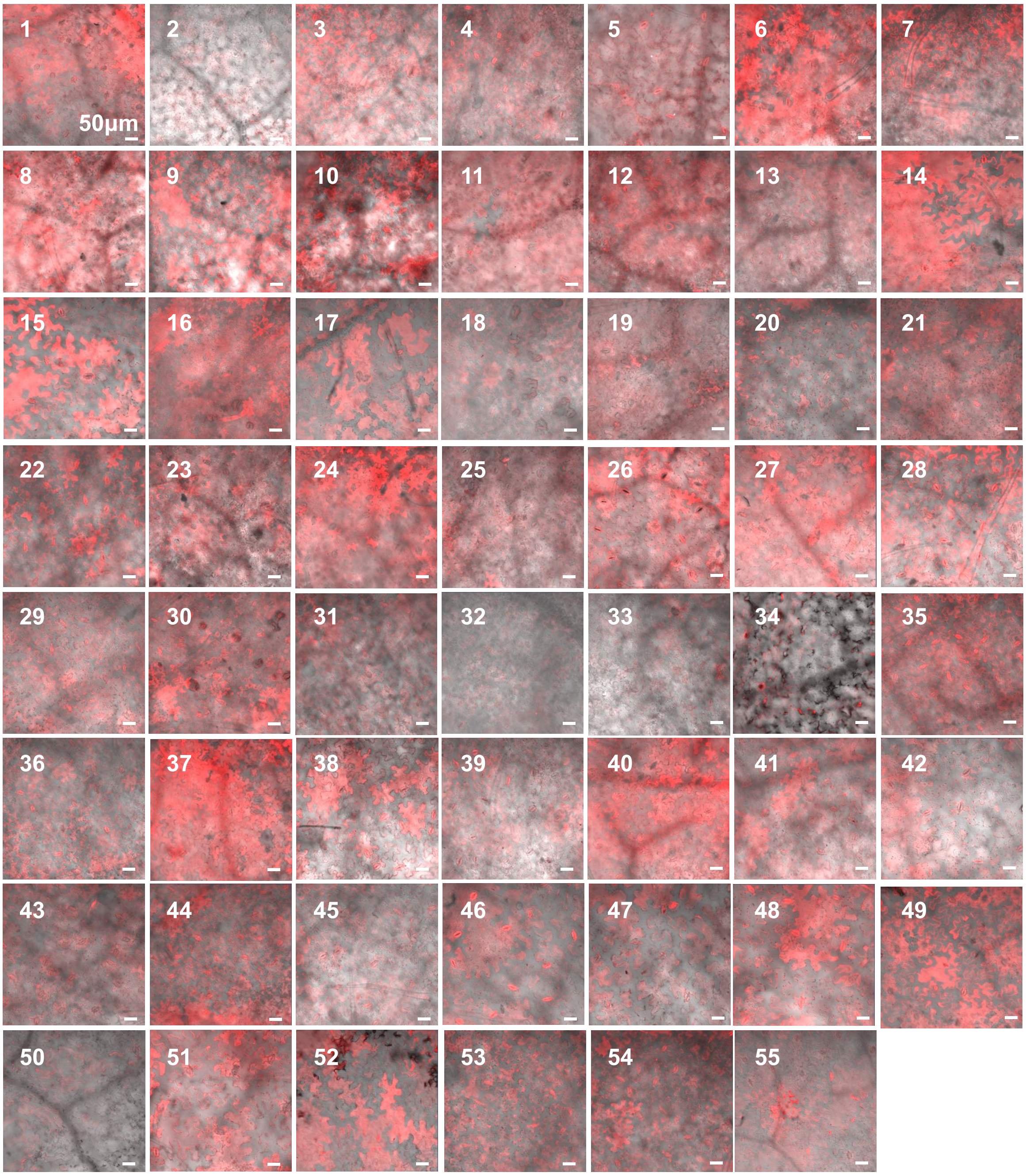

Figure S7. CLSM images of *N. benthamiana* leaf epidermal cells at 2 hours after infiltration of 55 types of CPP at 30ºC. The images are overlays of DIC (grey scale) and TAMRA fluorescence (red). The type of CPP is shown by peptide number and name as listed in Table 1.

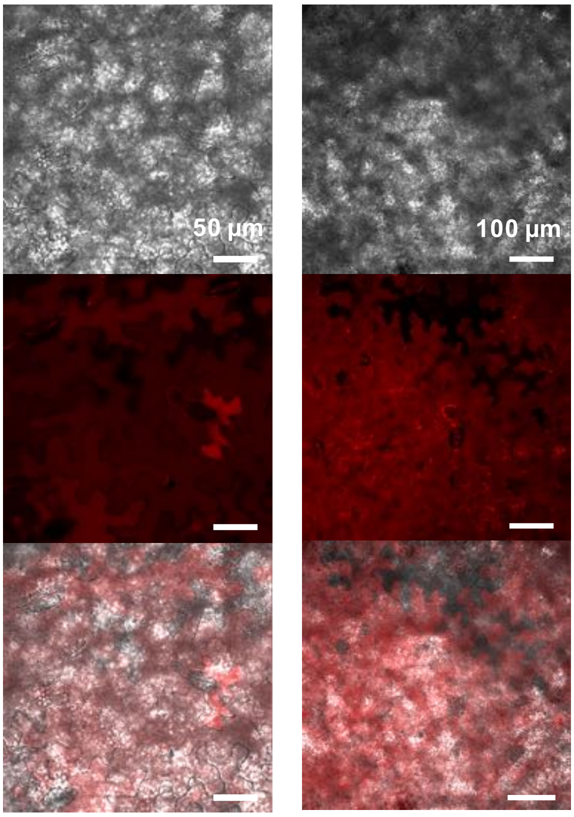

Figure S8. CLSM images of *N. benthamiana* cotyledons epidermal cells at 2 hours after infiltration of BP100 at 30ºC. Top, middle and bottom images are DIC, TAMRA fluorescence and overlay images, respectively.

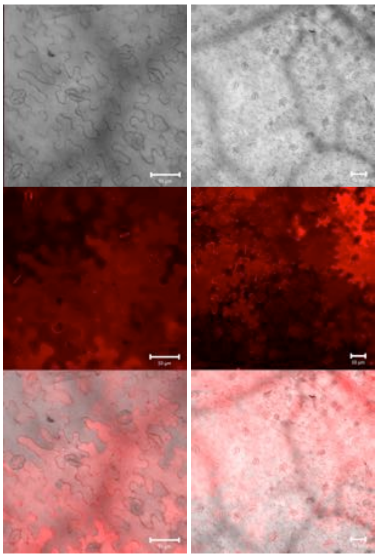

1. BP100

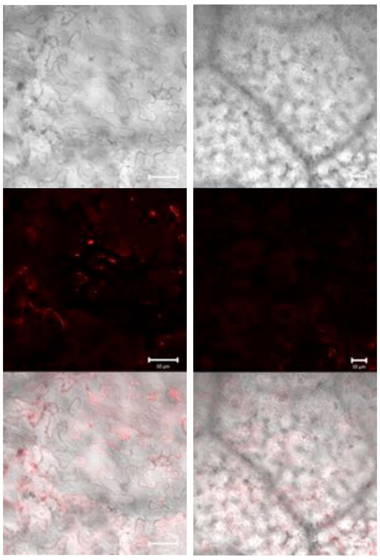

2. 2BP100

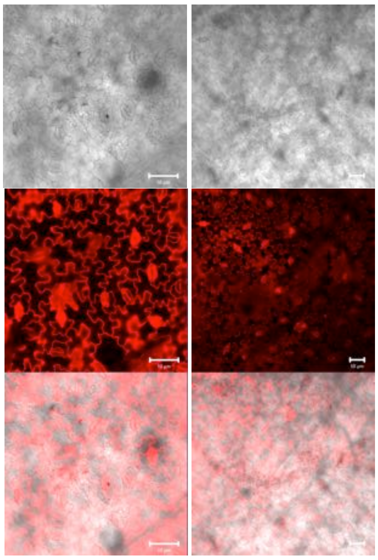

3. Rev(34-50)

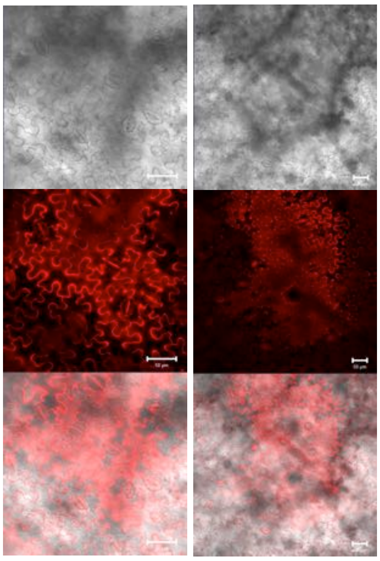

4. R9

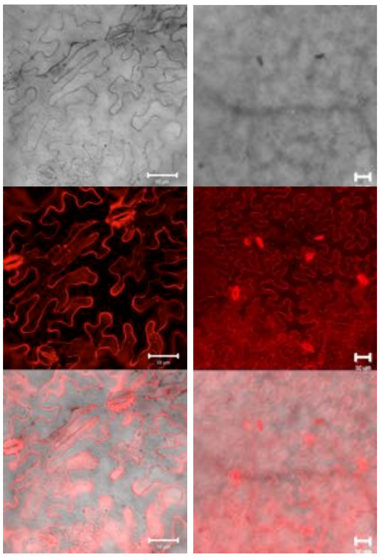

5. D-R9

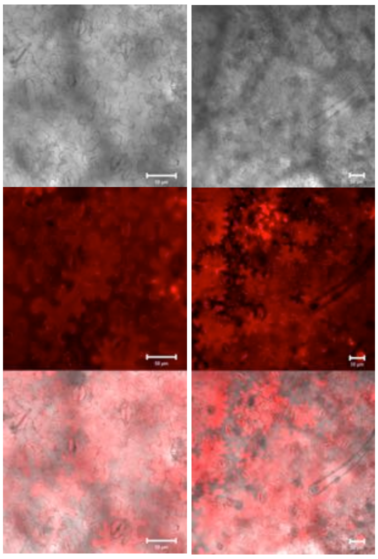

6. R12

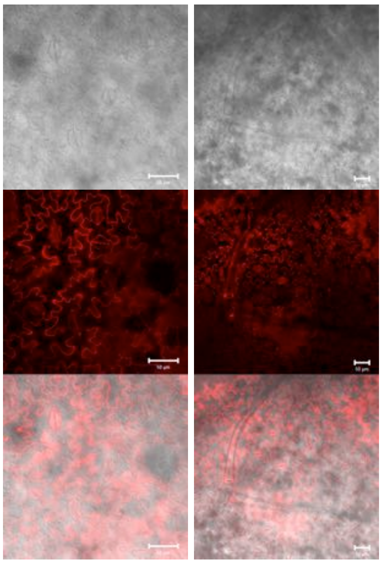

7. KH9

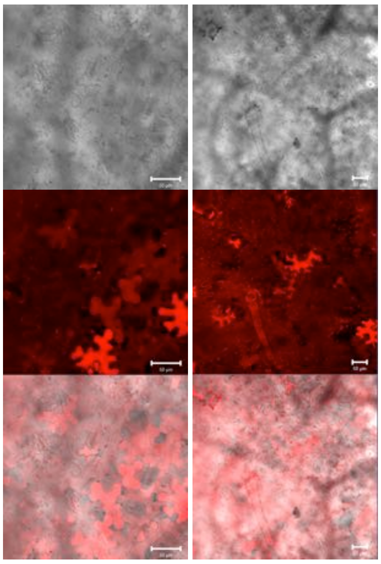

8. K9

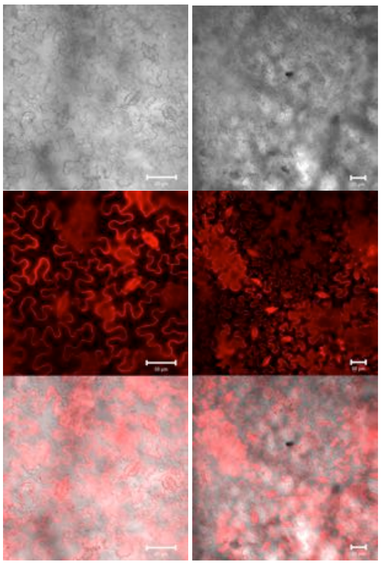

9. K18

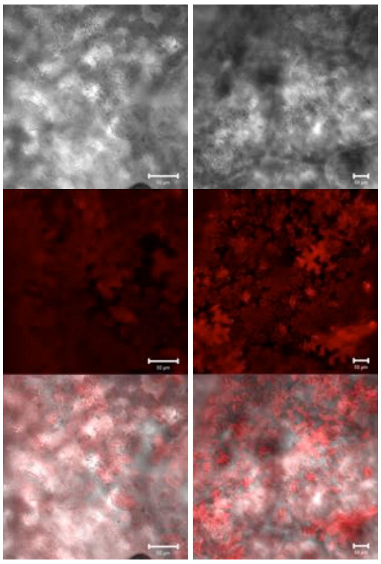

10. Pen2W2F

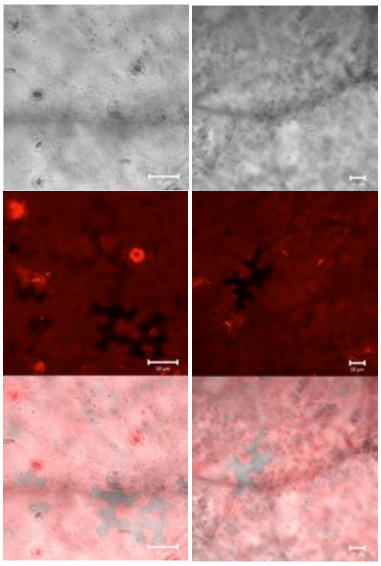

11. DPV3

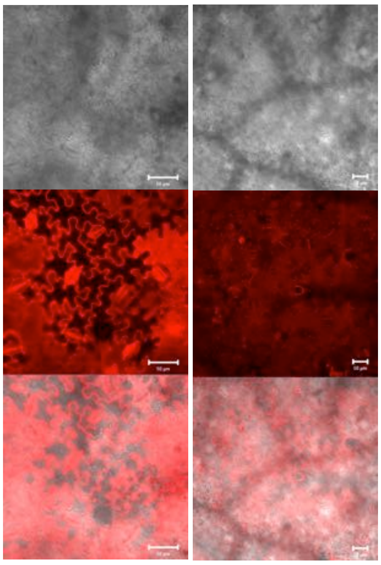

12. 6-Oct

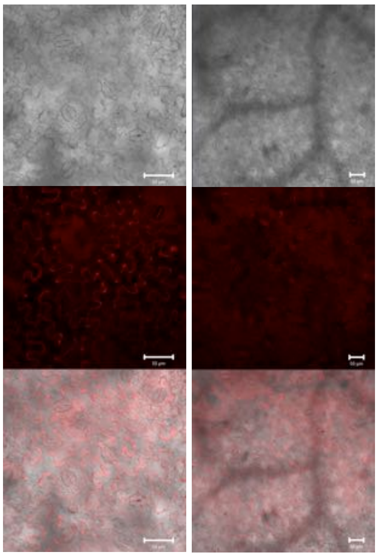
13. R9-TAT

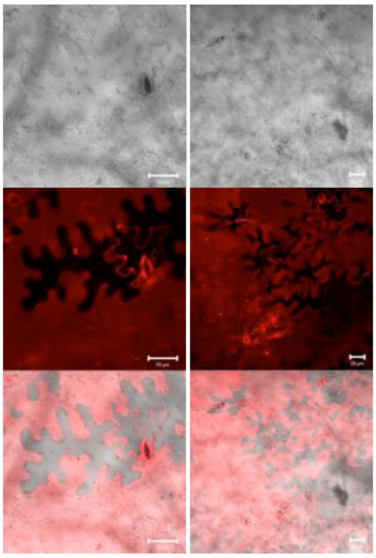
14. Tat(49-57)

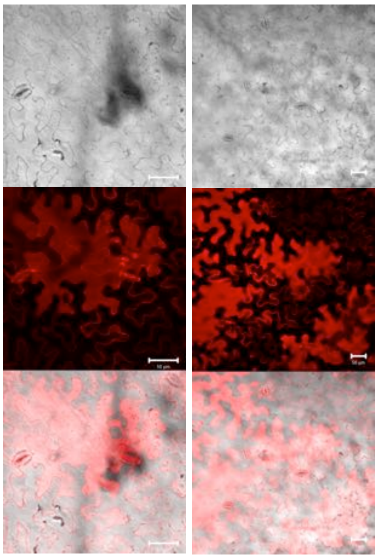
15. RetroTat(57-49)

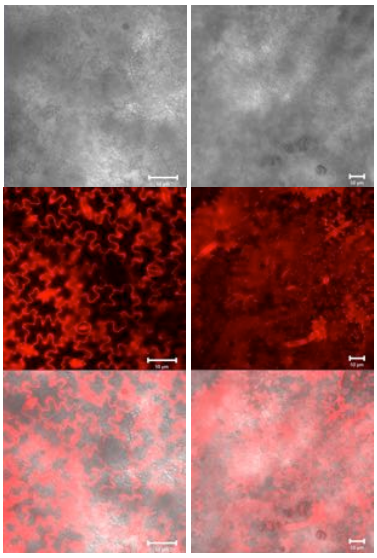
16. K18

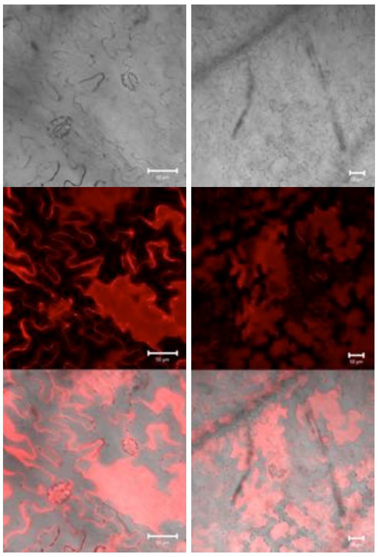
17. KLA10

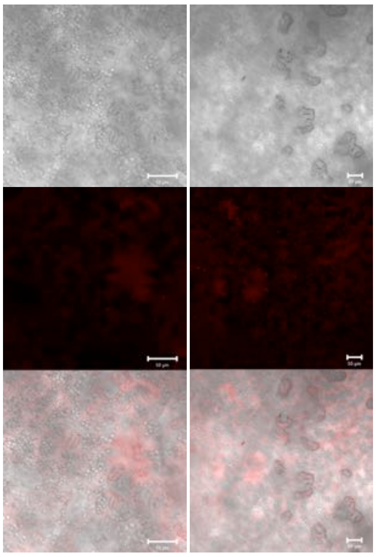
18. IX

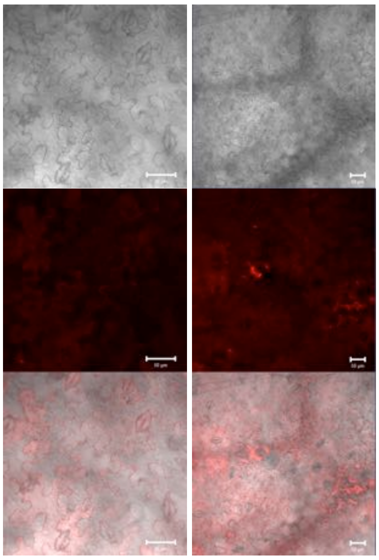

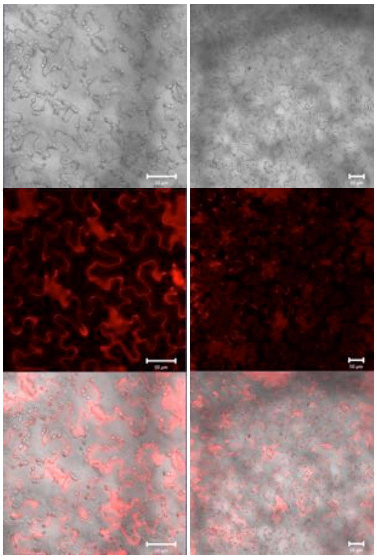

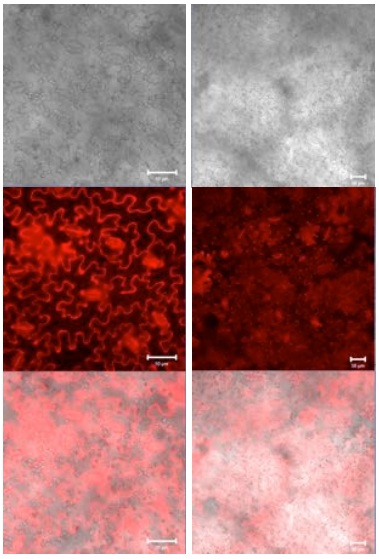

19. XI

20. No.14-12

21. pVEC

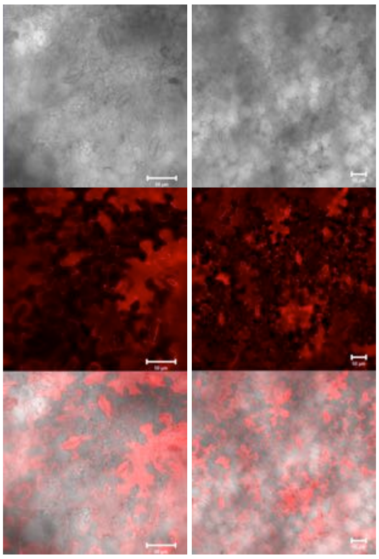

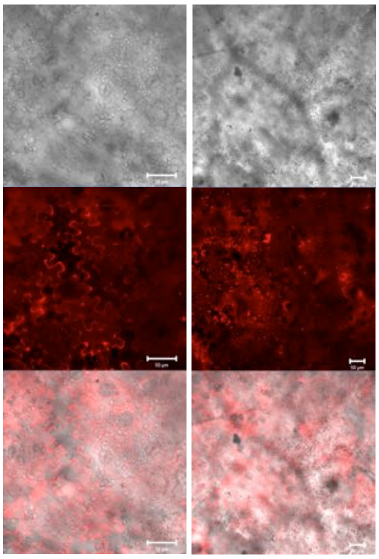

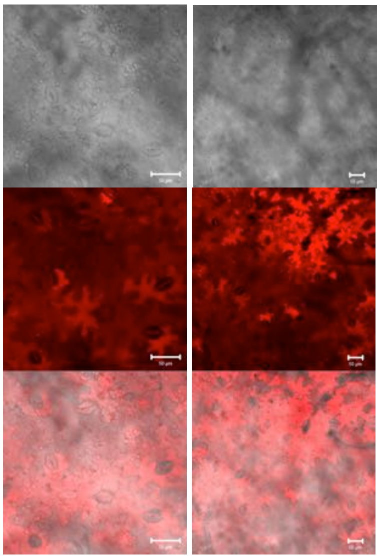

22. PenArg

23. M918

24. Penetratin

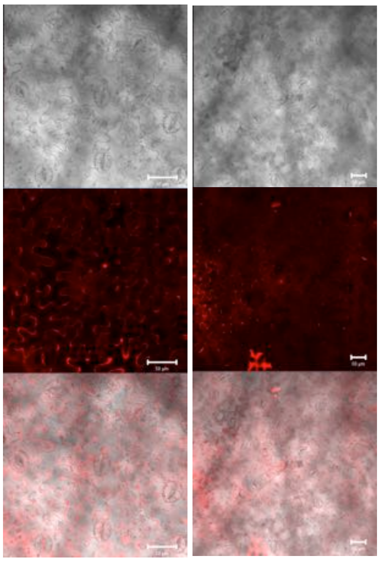

25. PolyP3(SAP)

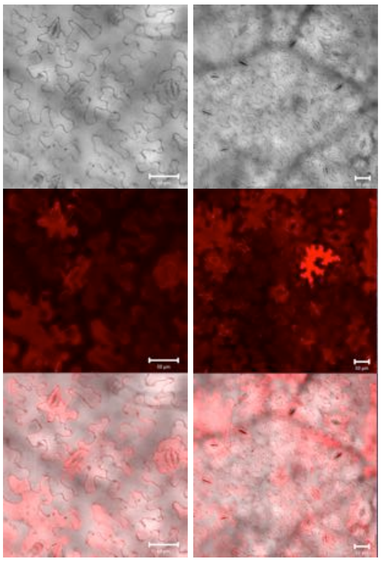

26. dhvar5

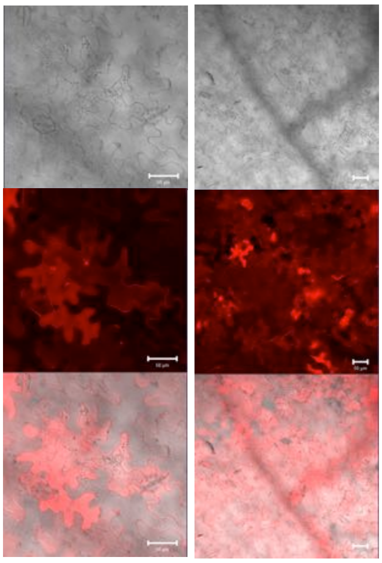

27.HPV33L2-445/467

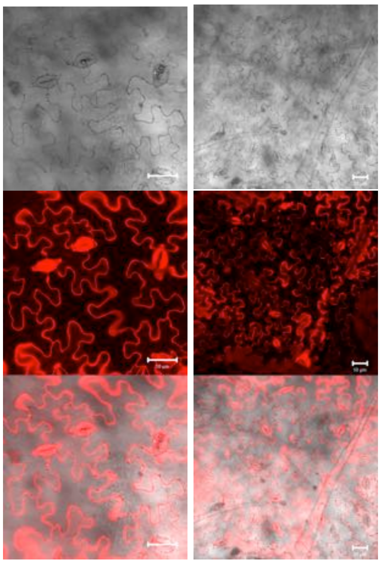

28. Buforin Ⅱ (5-21)

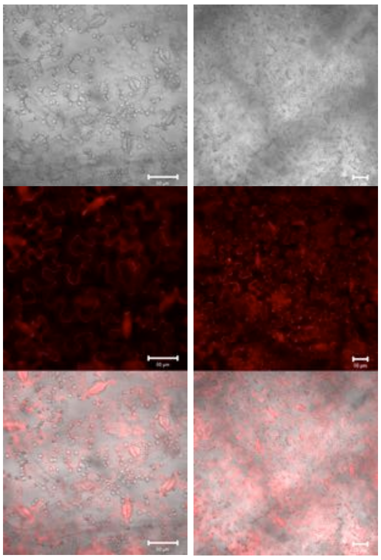

29. Scrambled pVEC

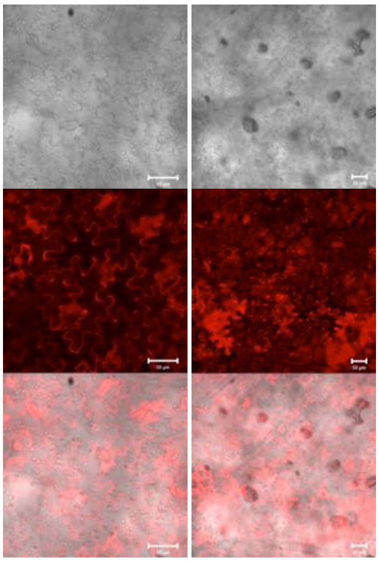

30. HPV33L2-DD447

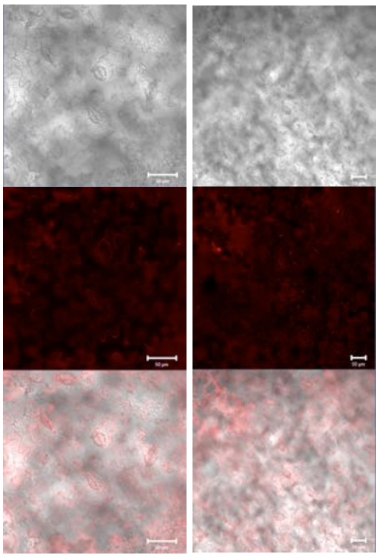

31. LAH4

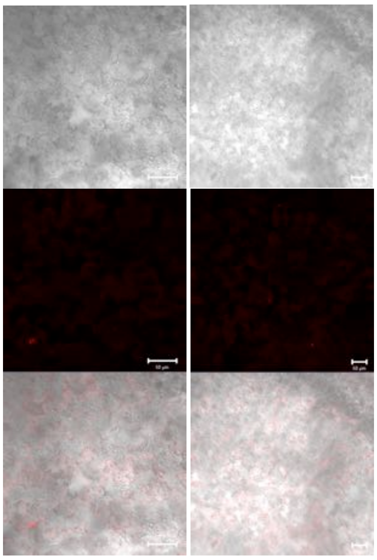

32. ppTG1

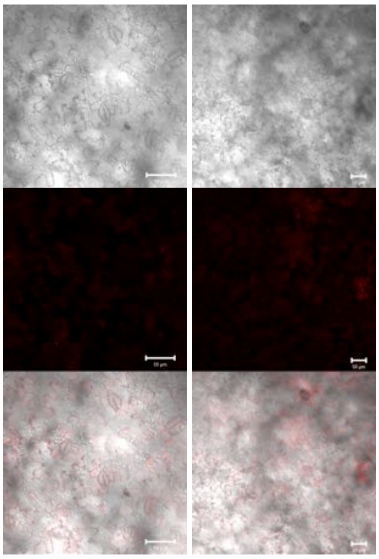

33. transportan(TP)

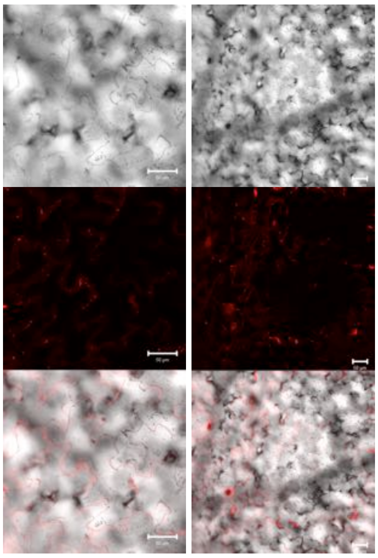

34. 2x ppTG1

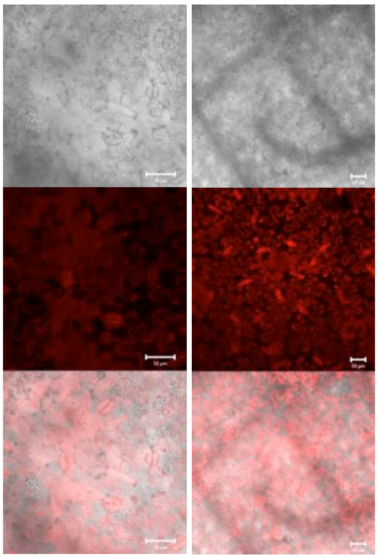

35. pAntpHD(Pro50)

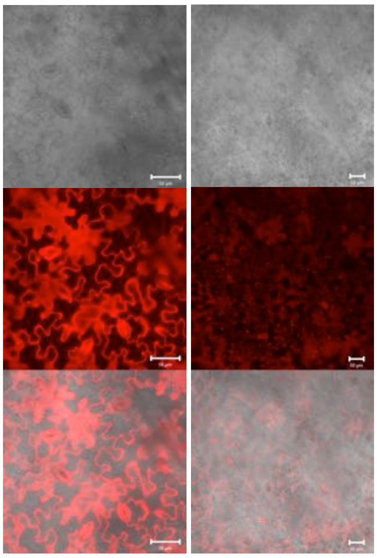

36. pAntp(44-58)

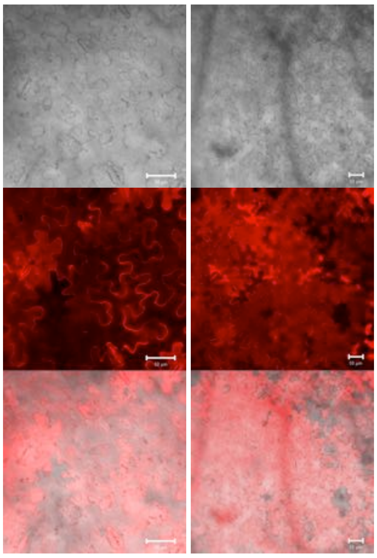

37. Crot(27-39)

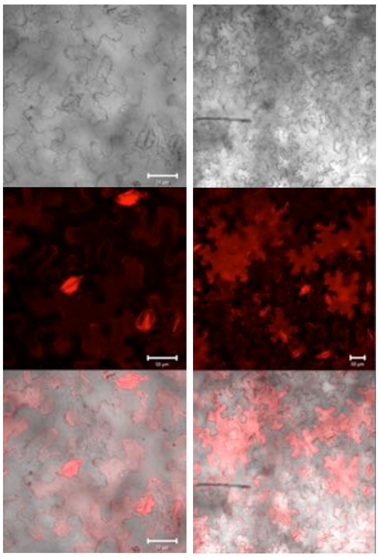

38. Crot(27-39) derevative1

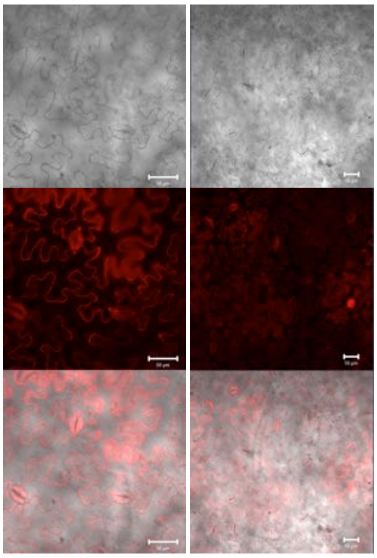

39. Crot(27-39) derevative2

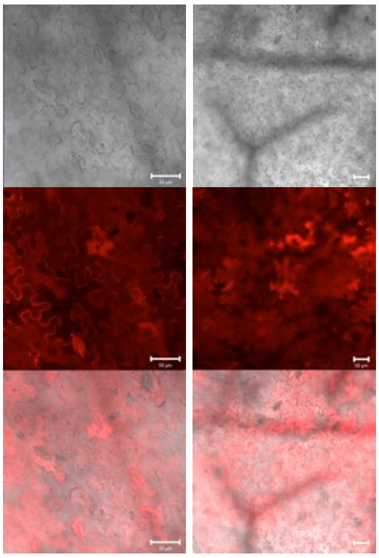

40. CyLoP-1

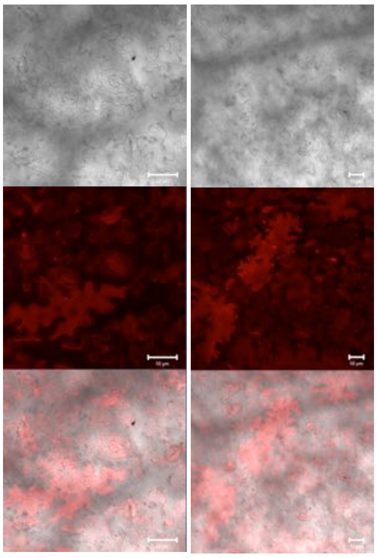

41. Inv3

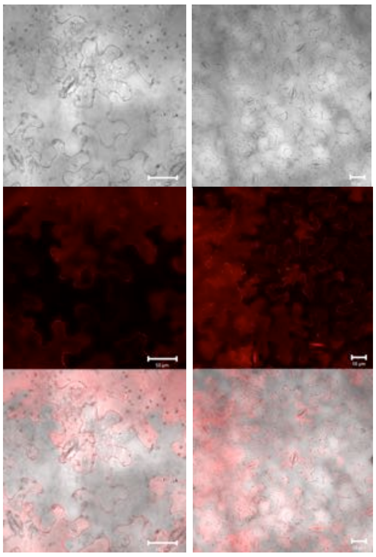

42. Inv5

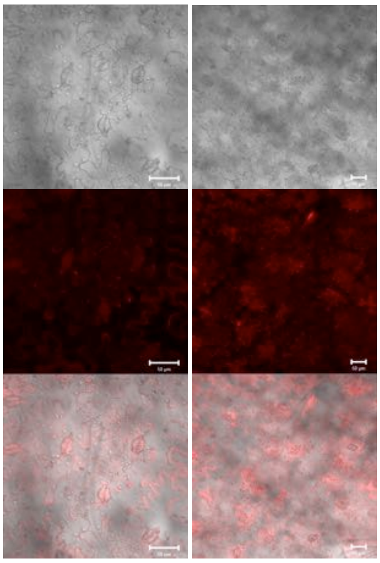

43. Inv3.5

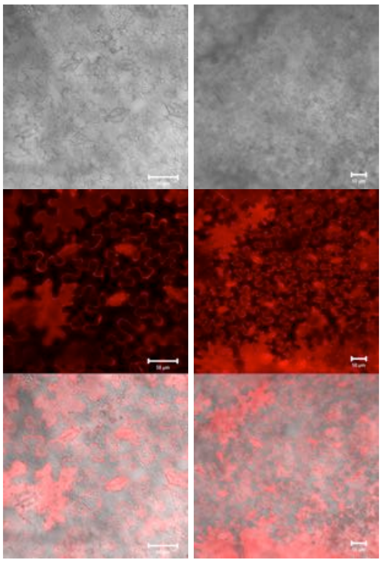

44. Inv3.10

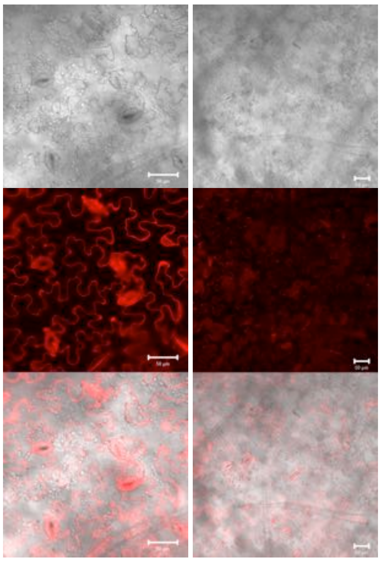

45. ARF(1-22)

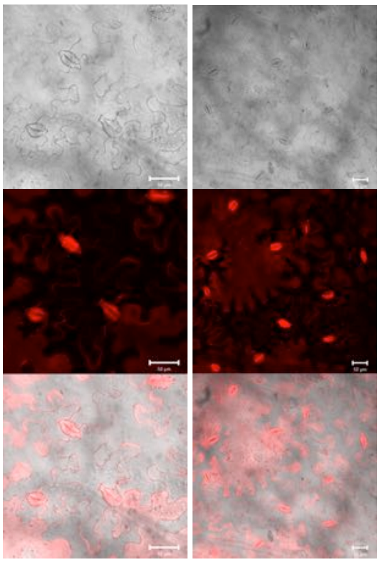

46. Cyt C 71-101

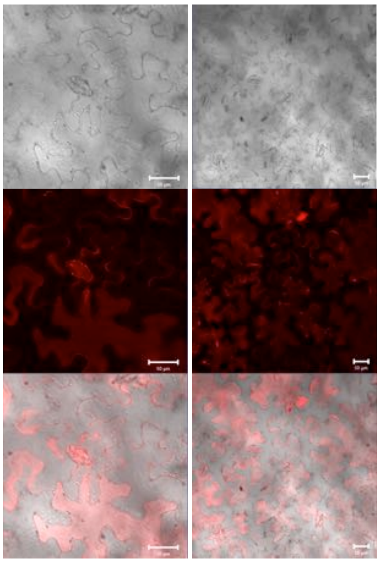

47. hLF peptide

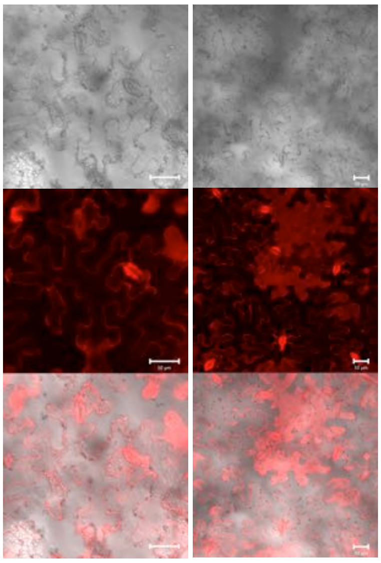

48. Glu-Oct-6

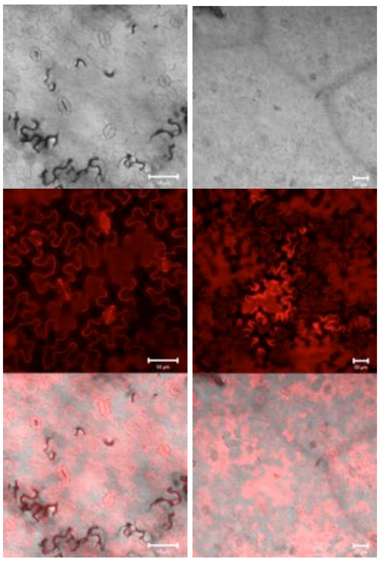

49. M511

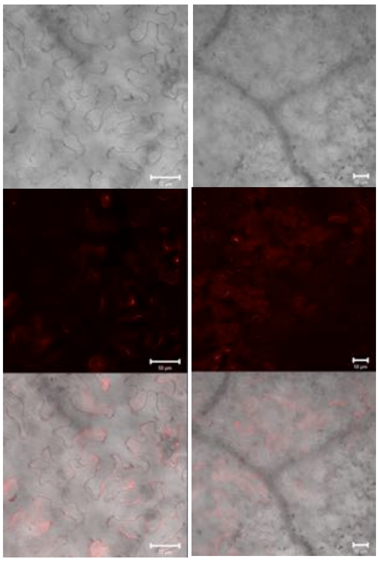

50. G53-4

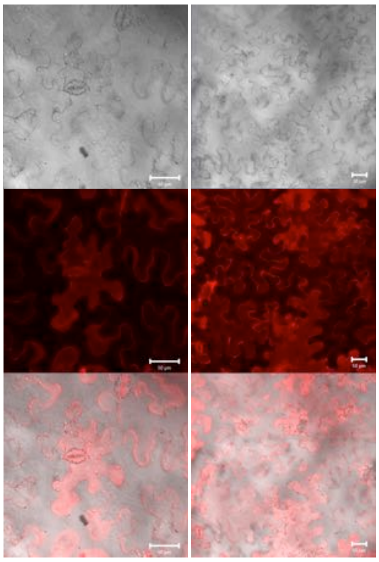

51. M591

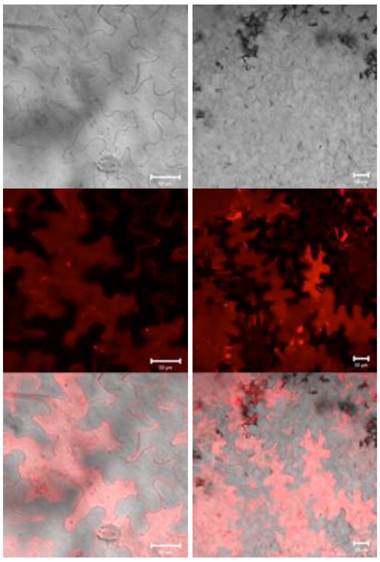

52. E162

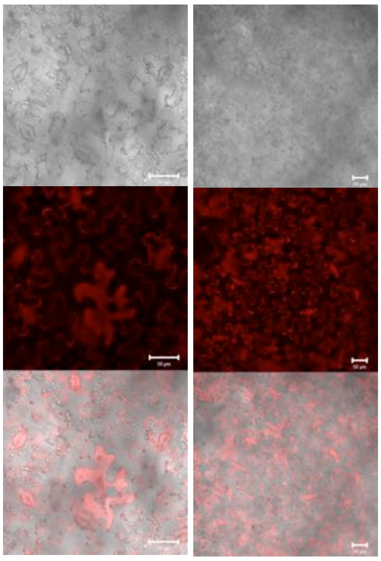

53. E165

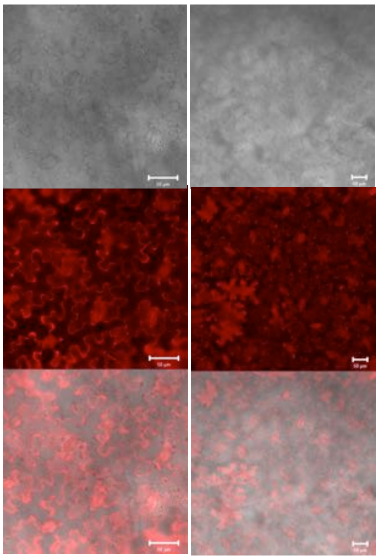

54. M867

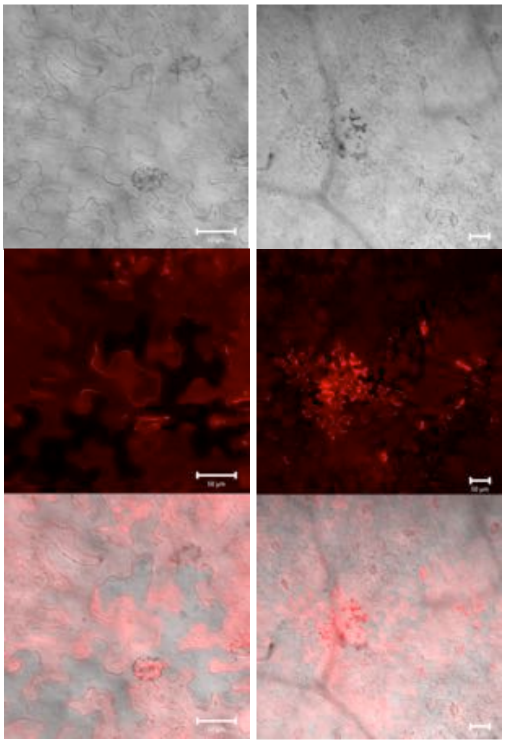

55. MG2d

Figure S9. CLSM images of *N. benthamiana* leaf epidermal cells at 2 hours after infiltration of 55 types of CPP at 30ºC. Top, middle and bottom images are DIC, TAMRA fluorescence and overlay images, respectively. The type of CPP is shown by peptide number and name as listed in Table 1.

Arabidopsis thaliana
High efficiency peptides

(a)

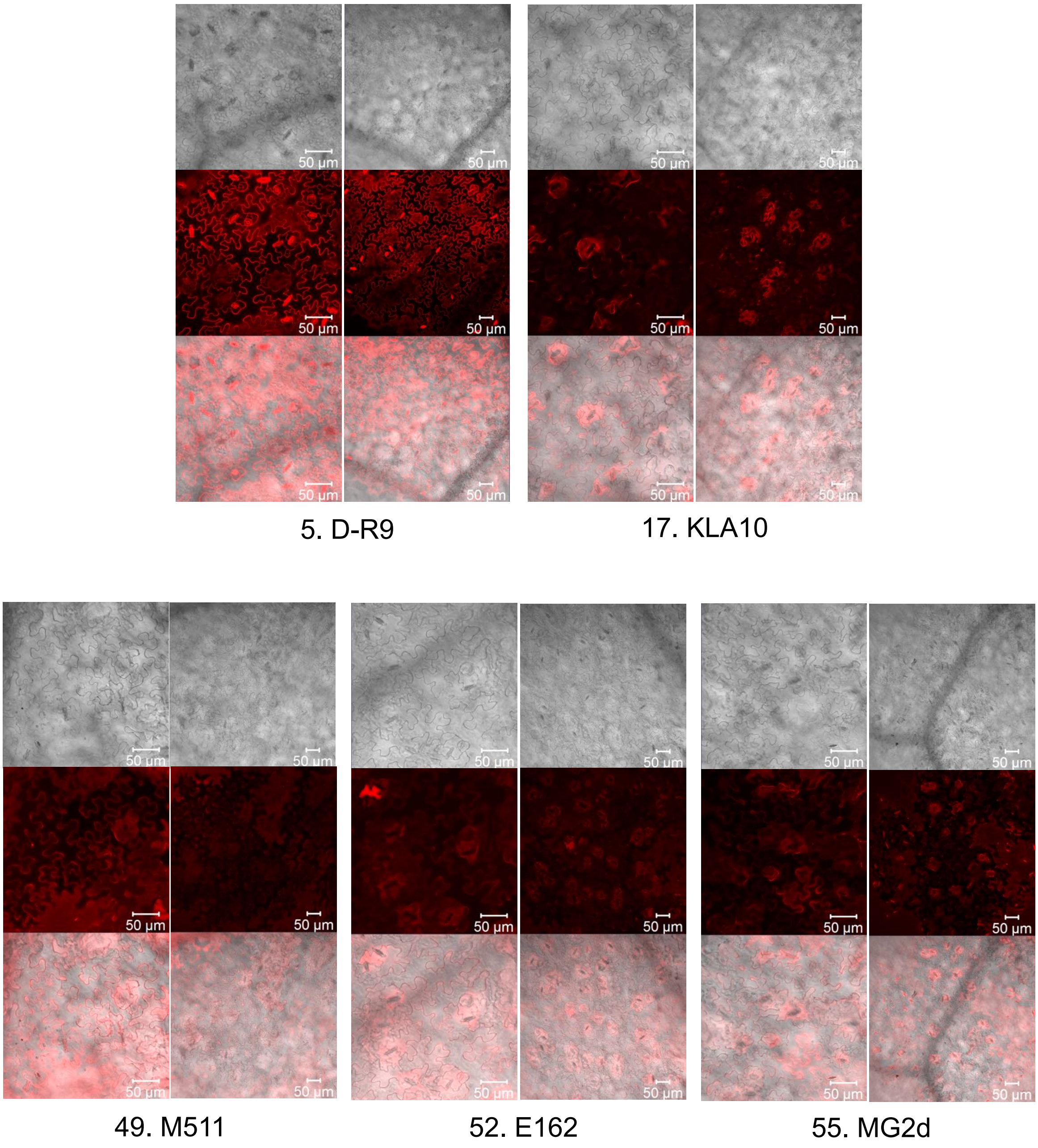

(b)

Arabidopsis thaliana

Middle efficiency peptides

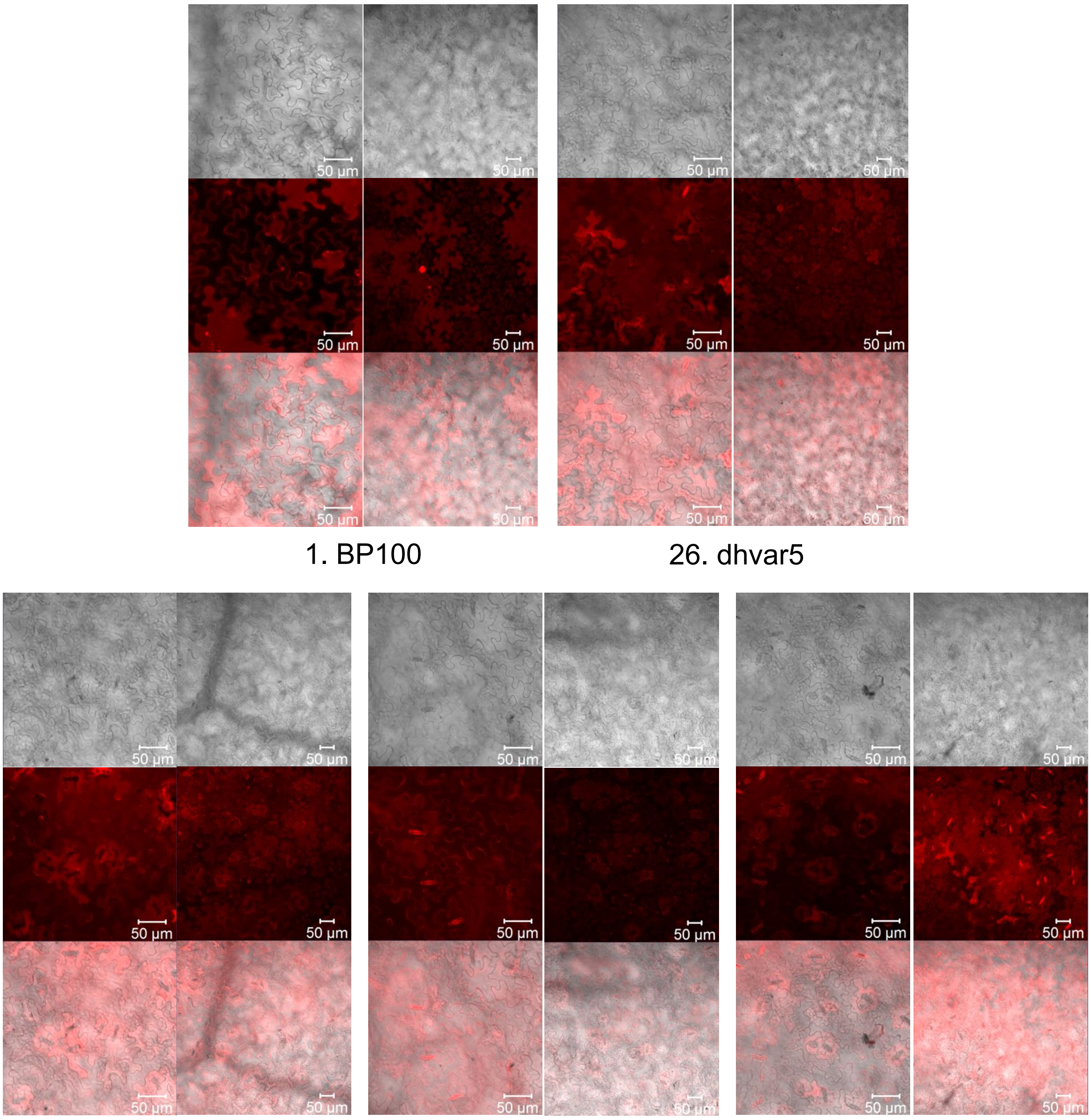

Arabidopsis thaliana

Low efficiency peptides

(c)

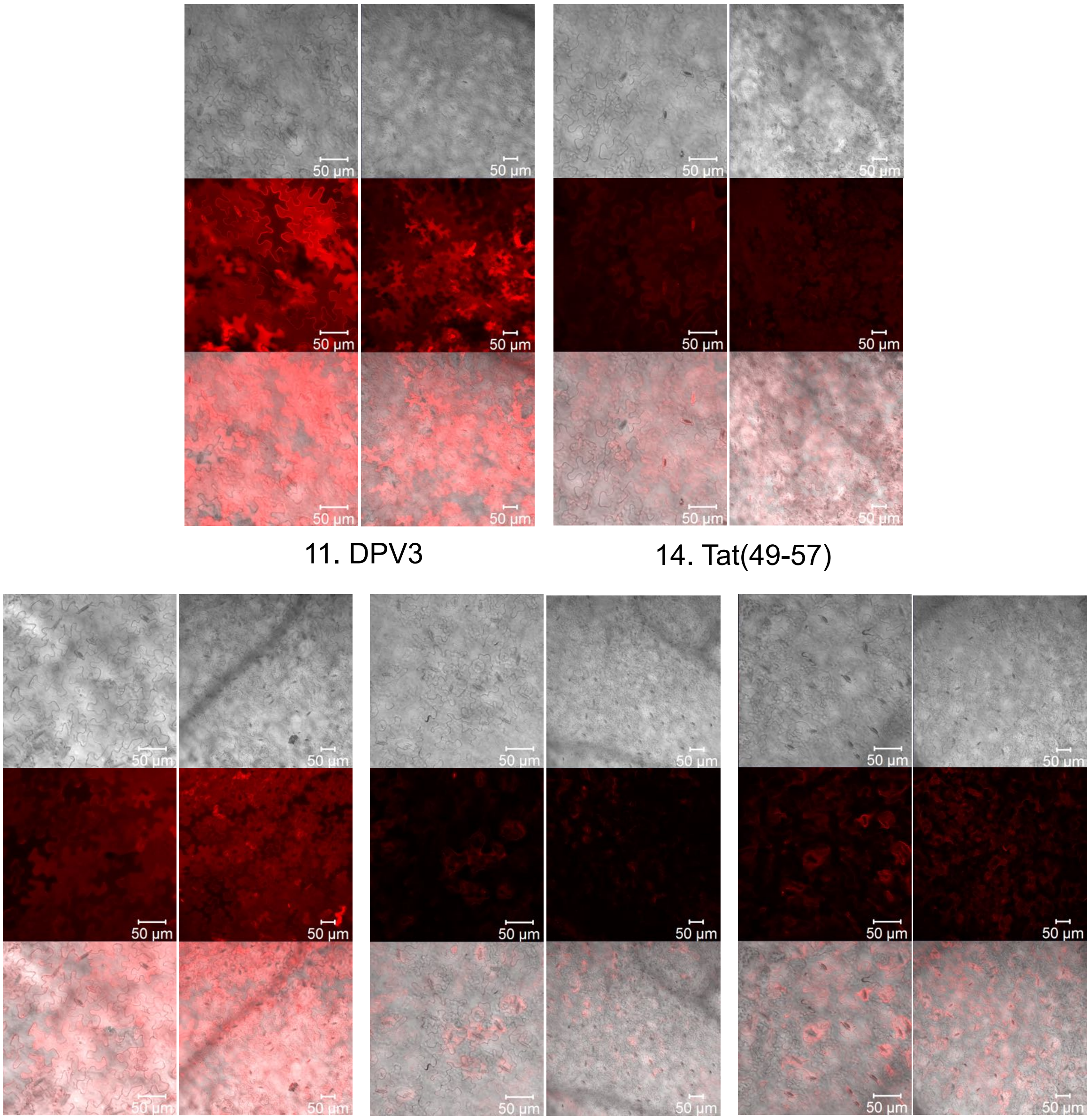

11. DPV3

14. Tat(49-57)

15. RetroTat(57-49)

34. 2x ppTG1

50. G53-4

Figure S10. CLSM images of *A. thaliana* leaf epidermal cells at 2 hours after infiltration of three groups of CPPs at 21ºC. Based on the results with BY-2 cells, the high efficient (a), middle efficient (b) and low efficient CPPs (c) were classified and assayed. Top, middle and bottom

images are DIC, TAMRA fluorescence and overlay images, respectively. The type of CPP is shown by peptide number and name as listed in Table 1.

(a)

Moneymaker
High efficiency peptides

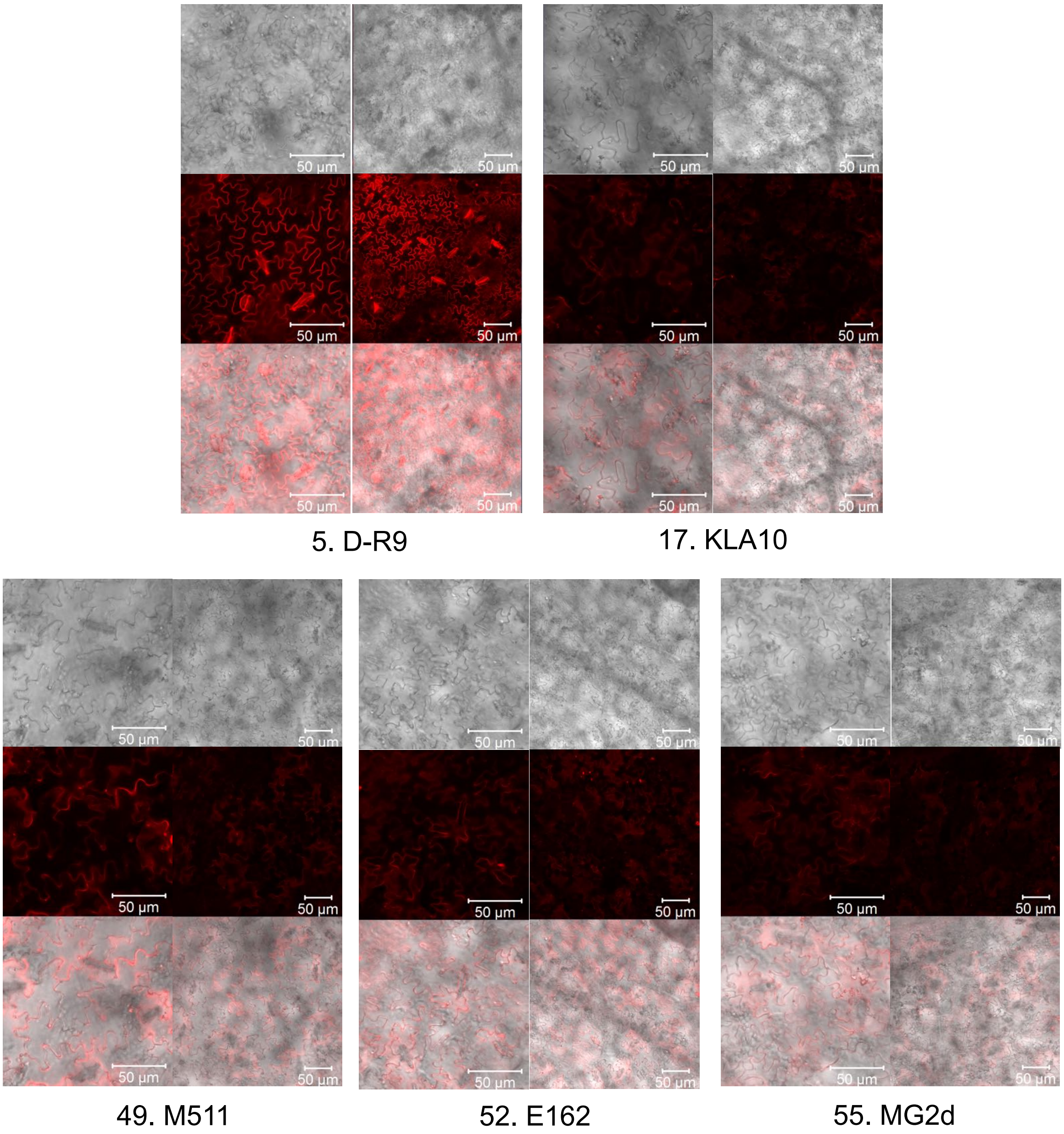

(b)

Moneymaker
Middle efficiency peptides

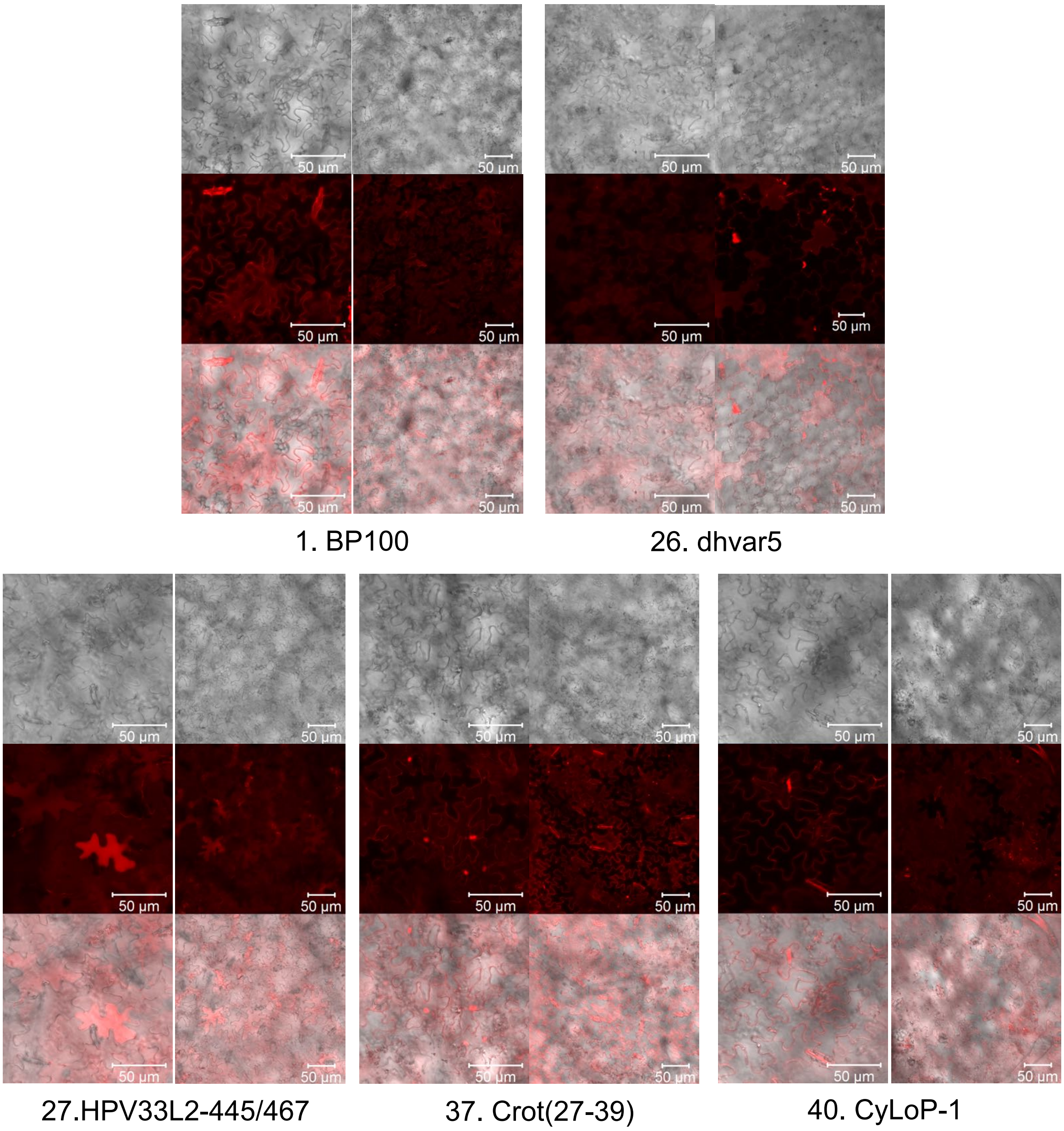

(c)

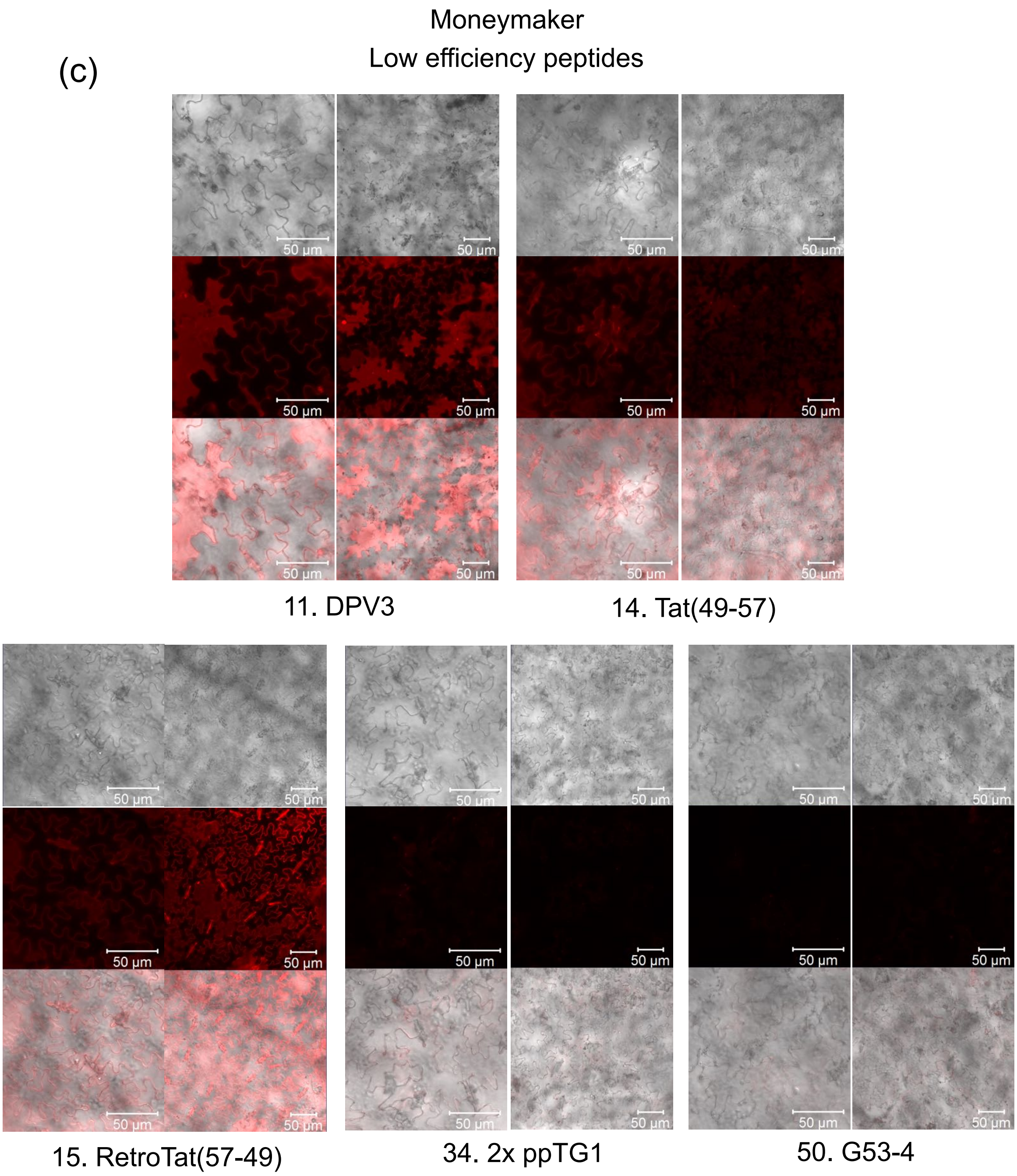

Figure S11. CLSM images of Moneymaker (*S. lycopersicum*) leaf epidermal cells at 2 hours after infiltration of three groups of CPPs at 26ºC. Based on the results with BY-2 cells, the high efficient (a), middle efficient (b) and low efficient CPPs (c) were classified and assayed with intact Moneymaker leaves. Top, middle and bottom images are DIC, TAMRA fluorescence and

overlay images, respectively. The type of CPP is shown by peptide number and name as listed in Table 1.

Poplar
High efficiency peptides

(a)

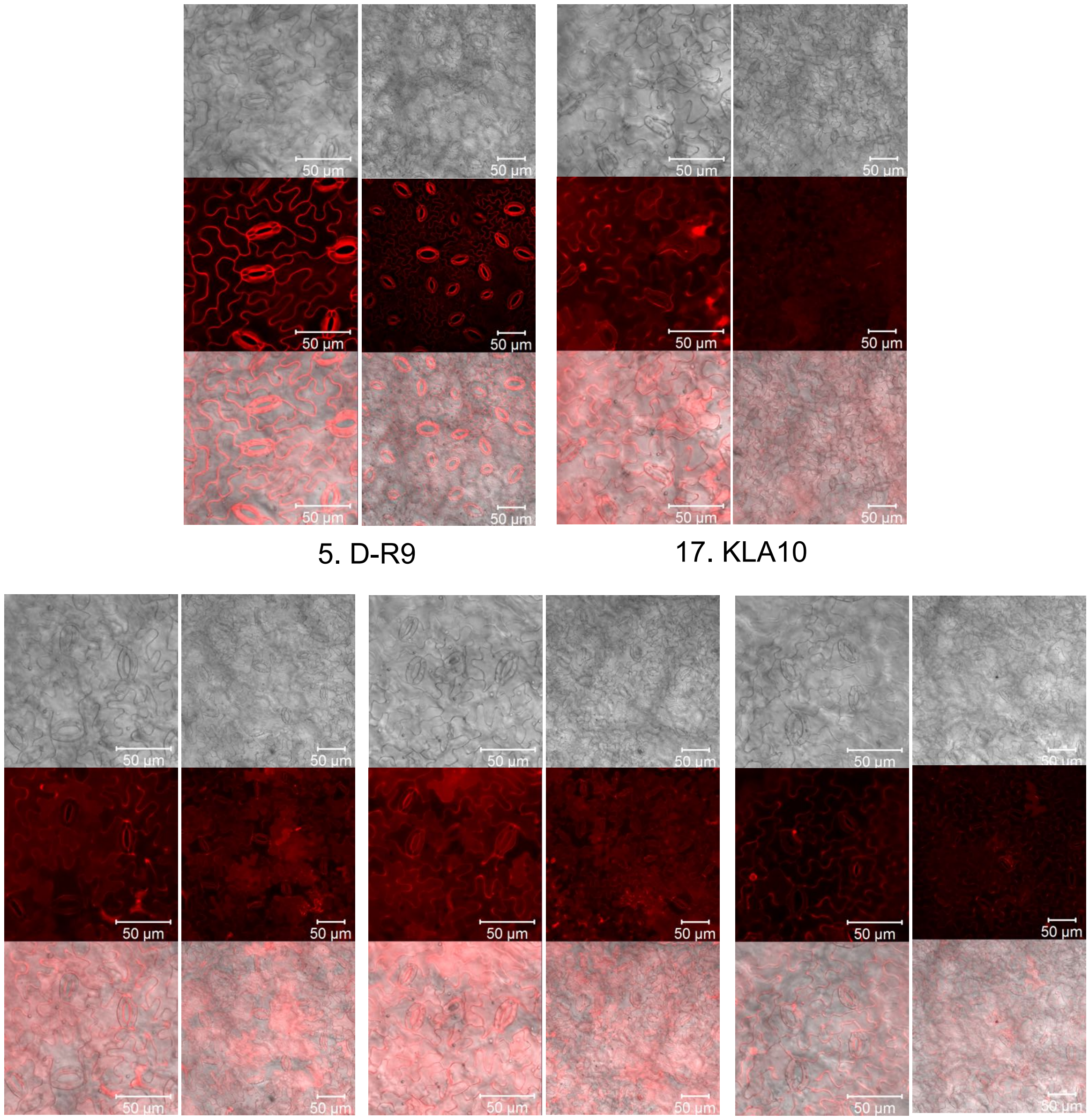

5. D-R9 17. KLA10

49. M511 52. E162 55. MG2d

(b)

Poplar

Middle efficiency peptides

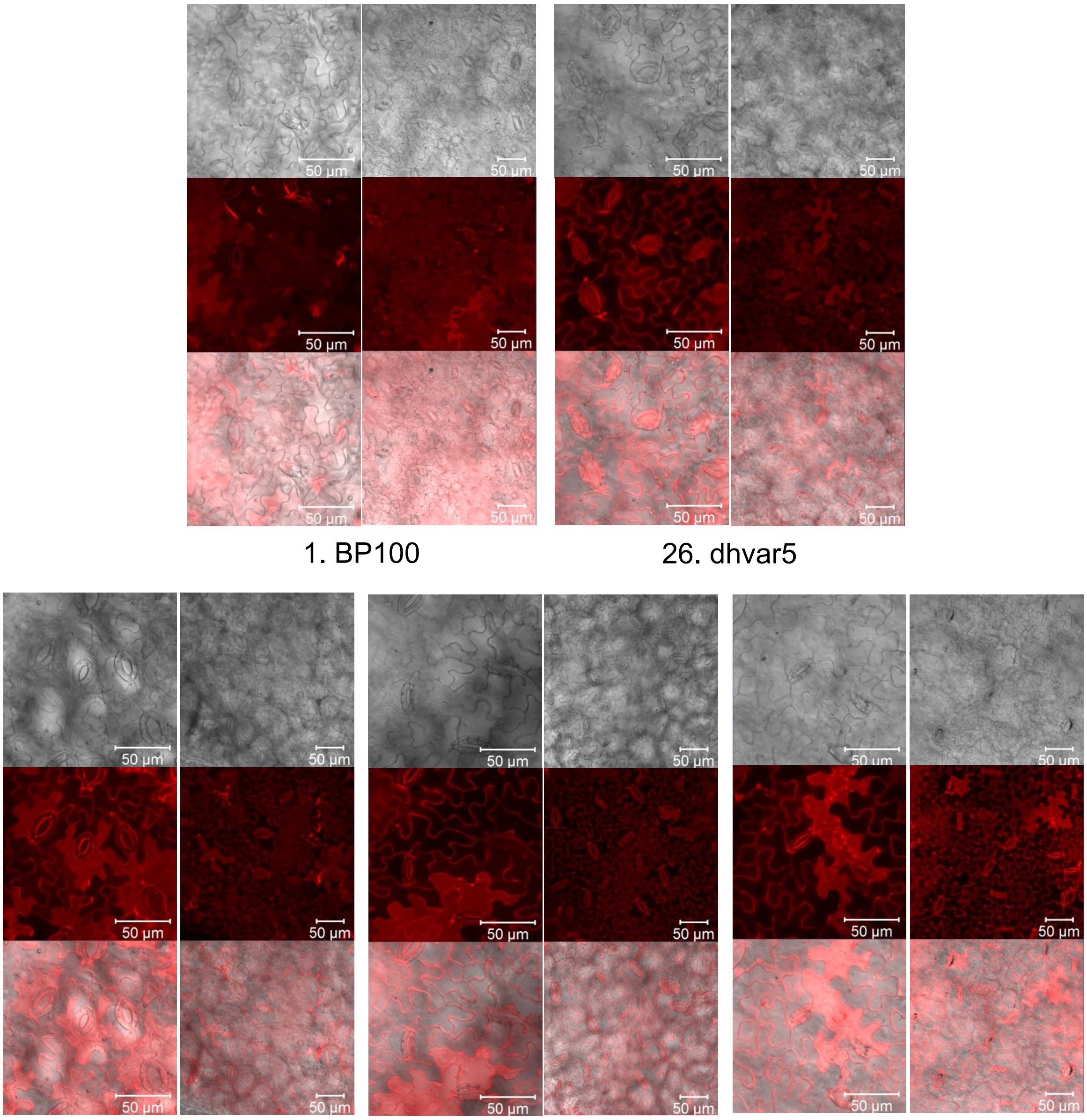

1. BP100 26. dhvar5

27.HPV33L2-445/467 37. Crot(27-39) 40. CyLoP-1

Poplar

Low efficiency peptides

(c)

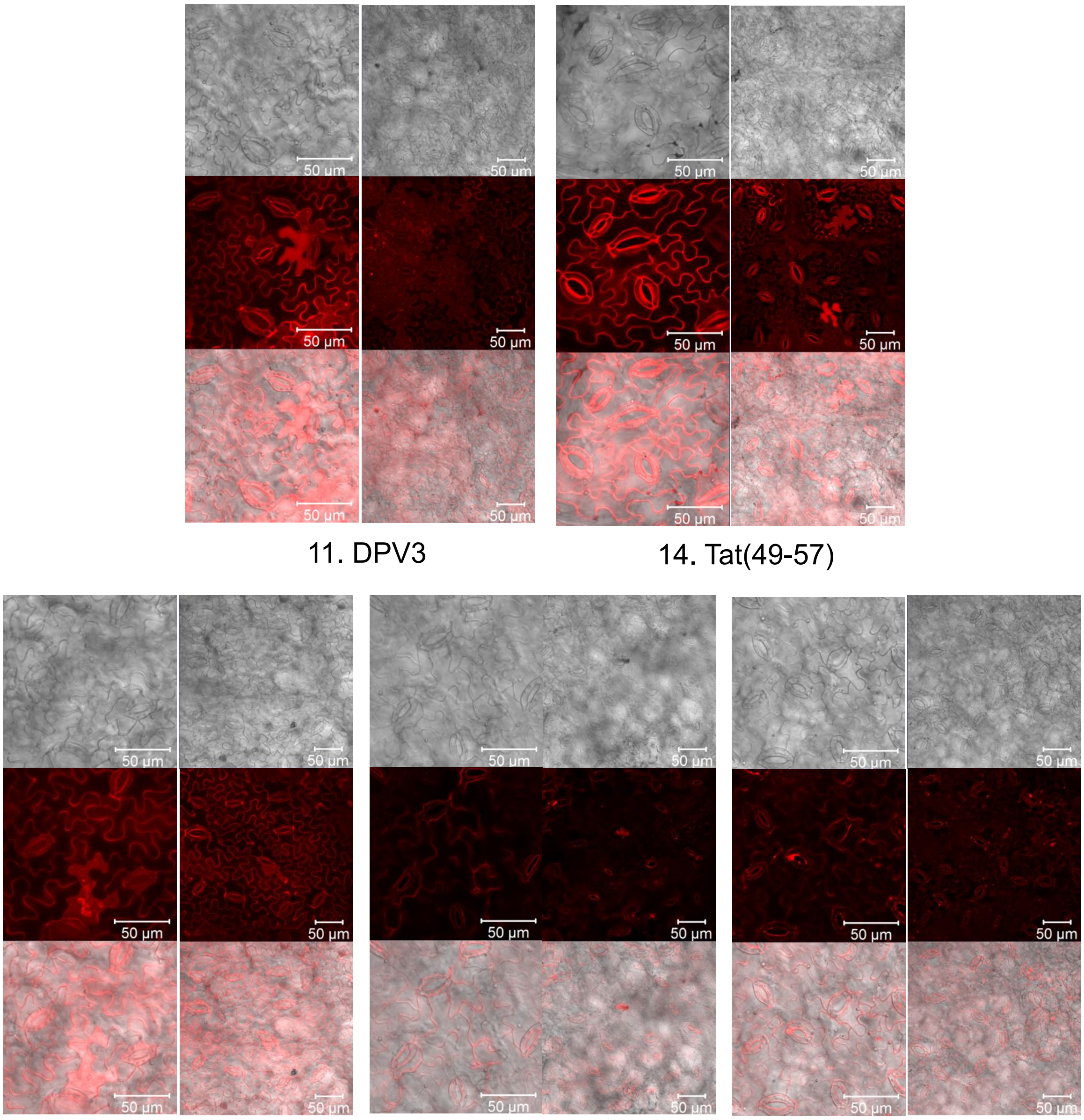

11. DPV3 14. Tat(49-57)

15. RetroTat(57-49) 34. 2x ppTG1 50. G53-4

Figure S12. CLSM images of poplar leaf epidermal cells at 2 hours after infiltration of three groups of CPPs at 22ºC. Based on the results with BY-2 cells, the high efficient (a), middle efficient (b) and low efficient CPPs (c) were classified and assayed with intact poplar leaves. Top, middle and bottom images are DIC, TAMRA fluorescence and overlay images, respectively. The type of CPP is shown by peptide number and name as listed in Table 1.

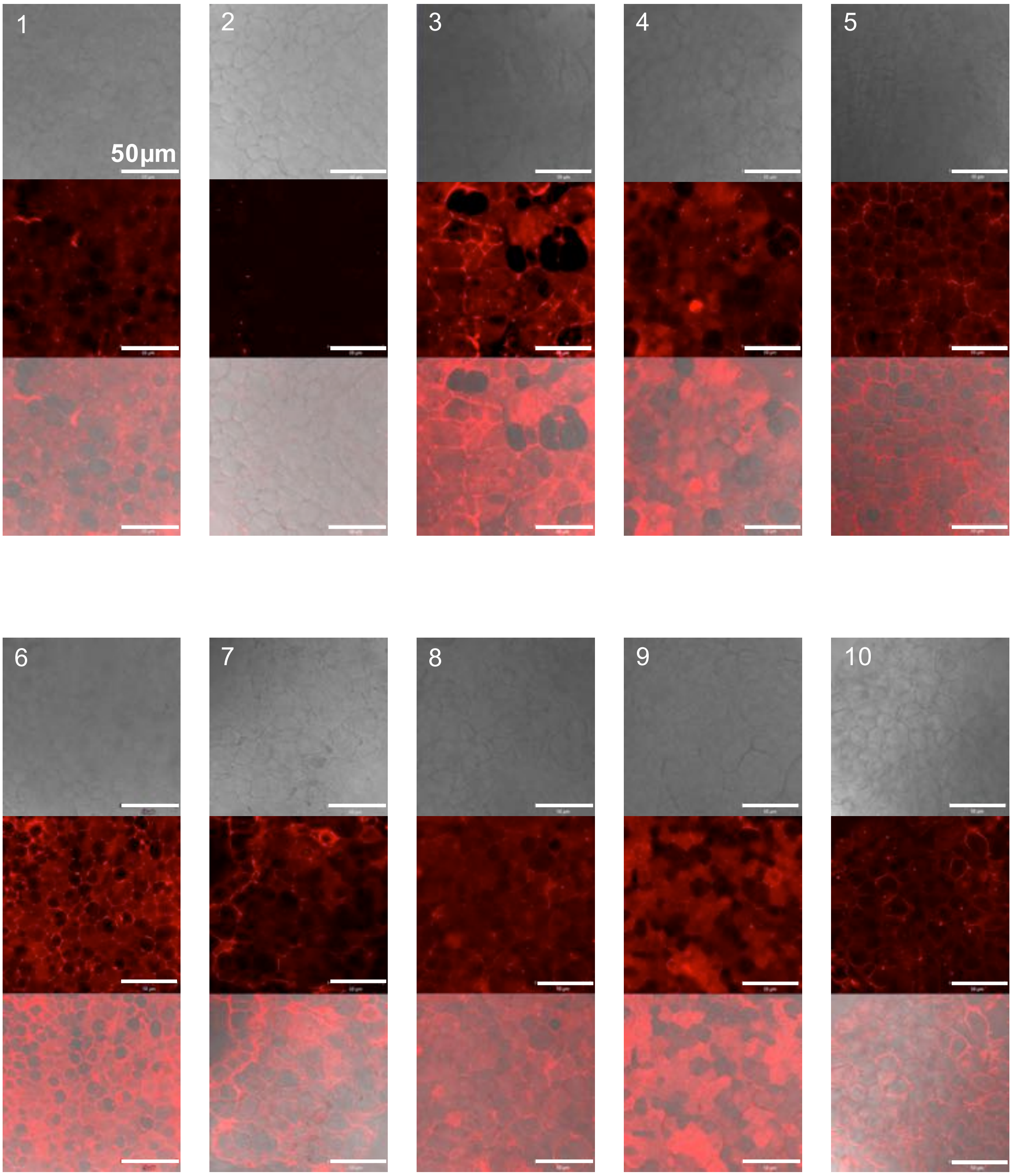
1
2
3
4
5
50µm
6
7
8
9
10

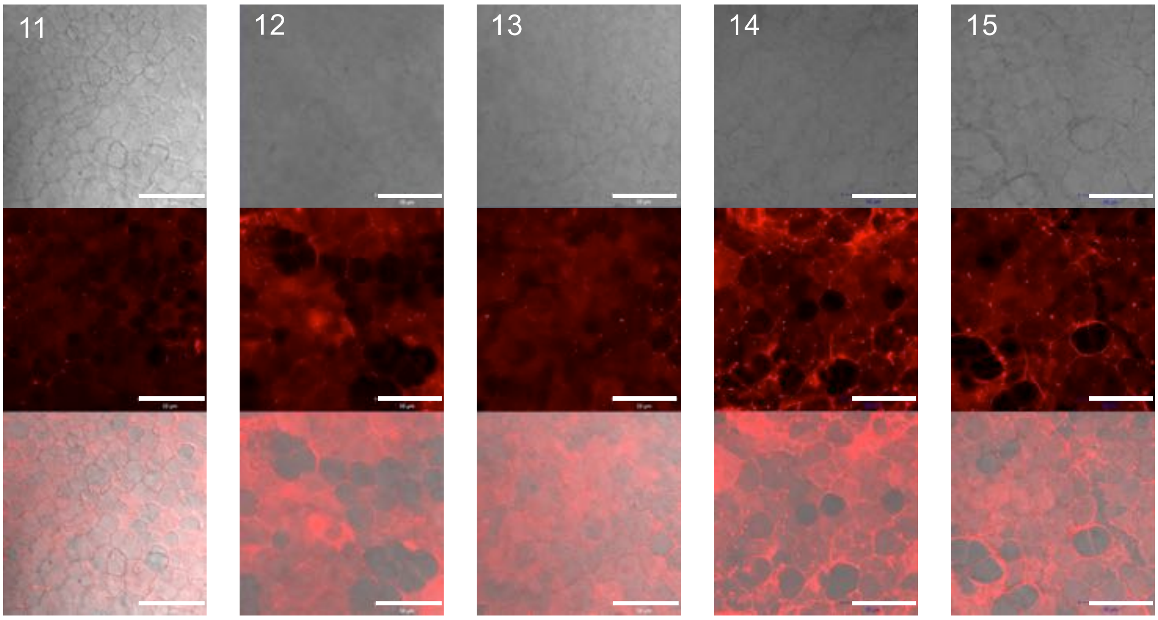
11
12
13
14
15

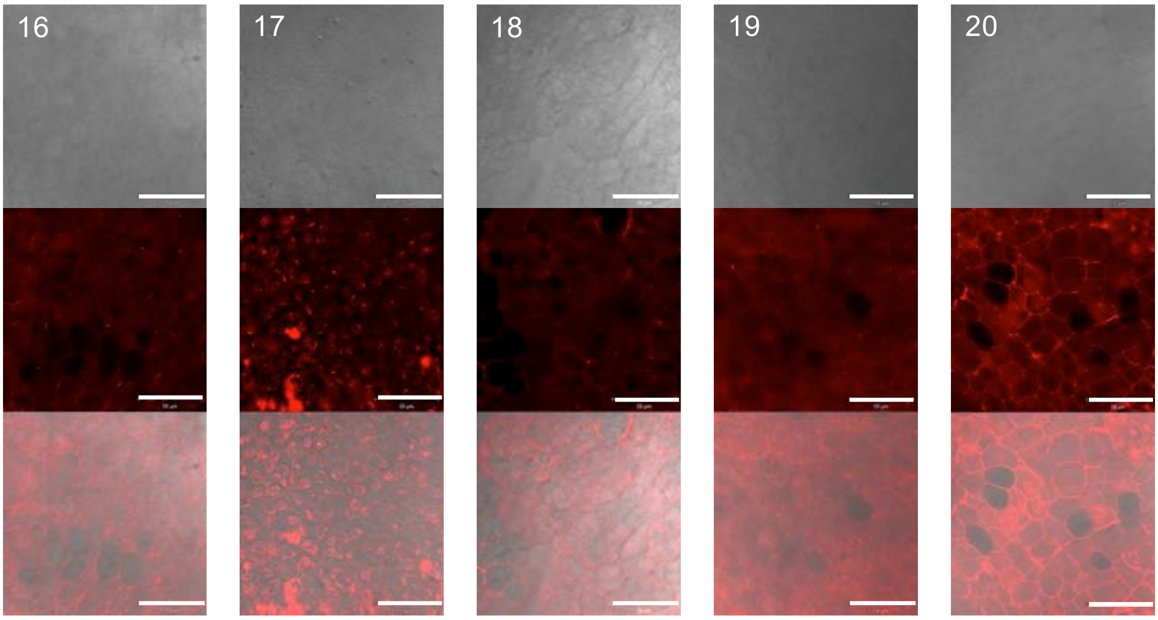
16
17
18
19
20

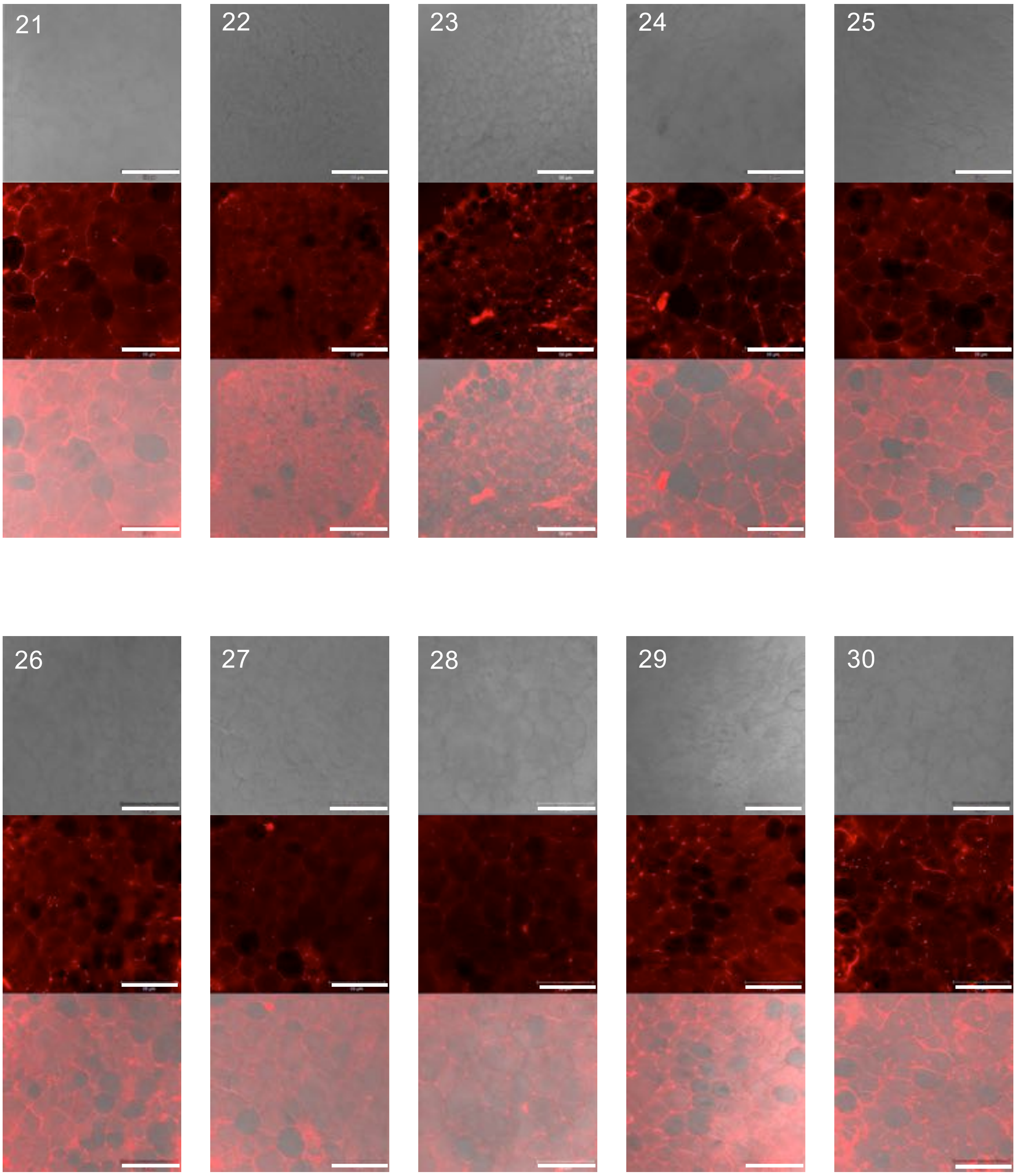
21
22
23
24
25
26
27
28
29
30

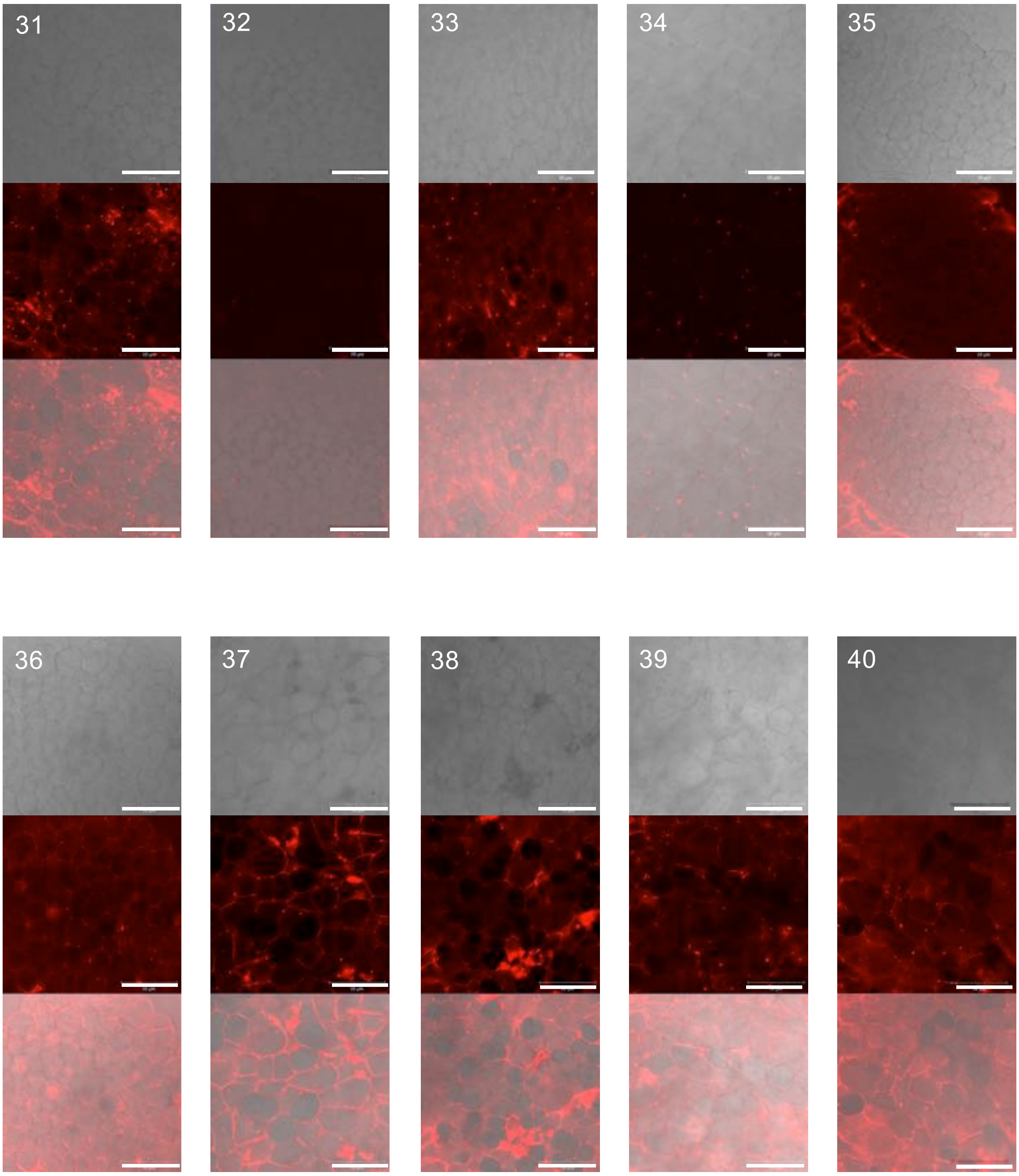
31
32
33
34
35
36
37
38
39
40

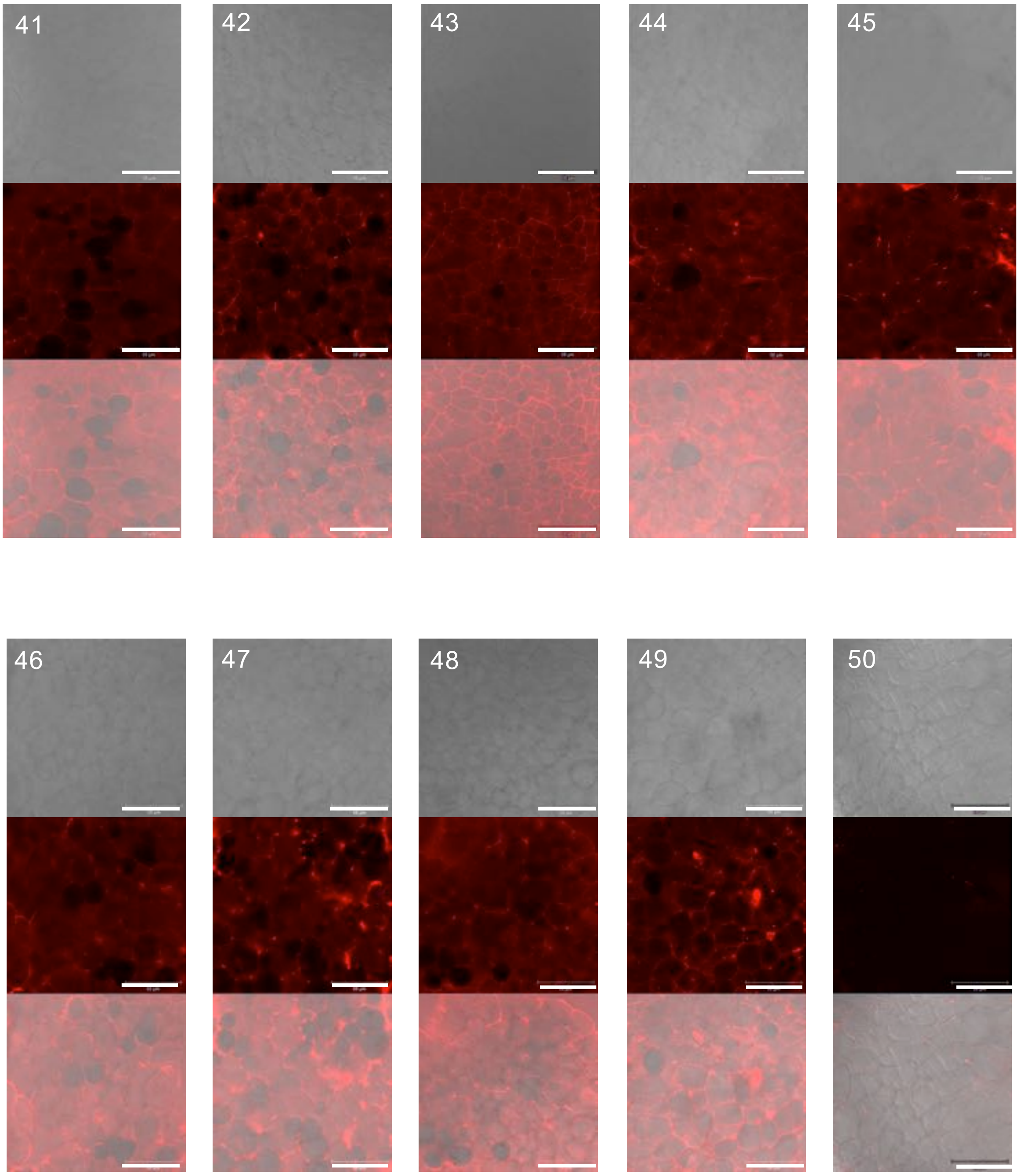
41
42
43
44
45
46
47
48
49
50

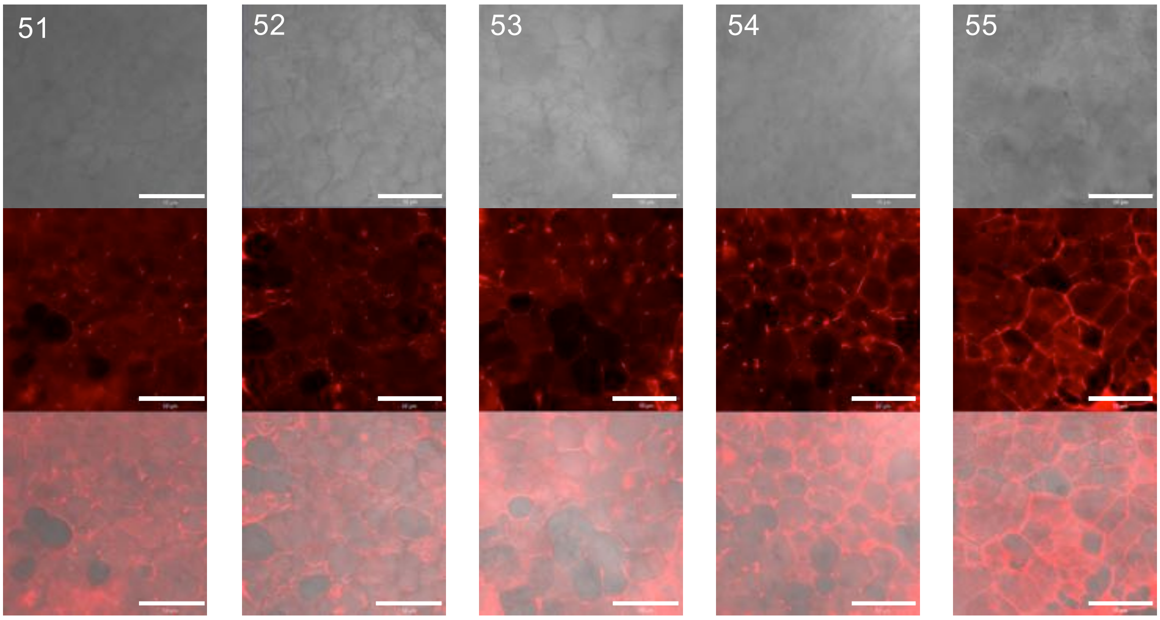

Figure S13. CLSM images of rice (*O. sativa*) callus at 2 hours after infiltration of 55 CPPs at 22ºC. Top, middle and bottom images are DIC, TAMRA fluorescence and overlay images, respectively. The type of CPP is shown by peptide number and name as listed in Table 1. Scale bar, 50 µm.

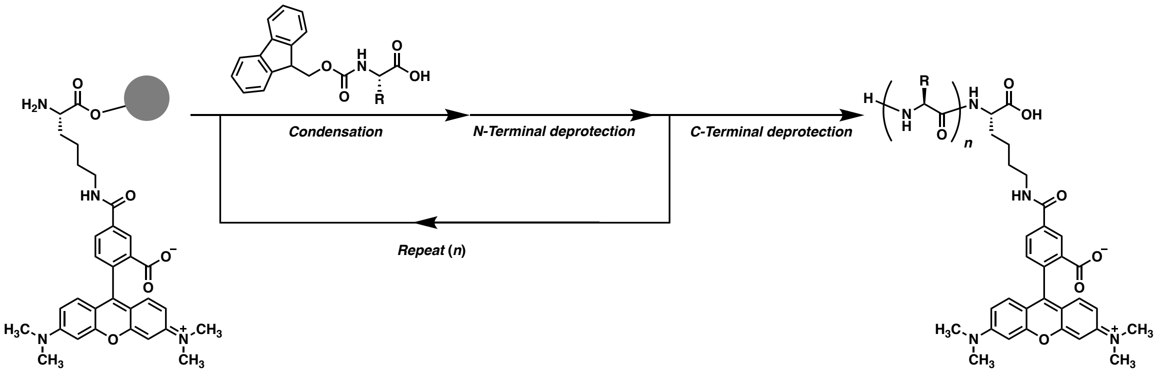

Figure S14. Reaction scheme to synthesize TAMRA-labeled CPP.

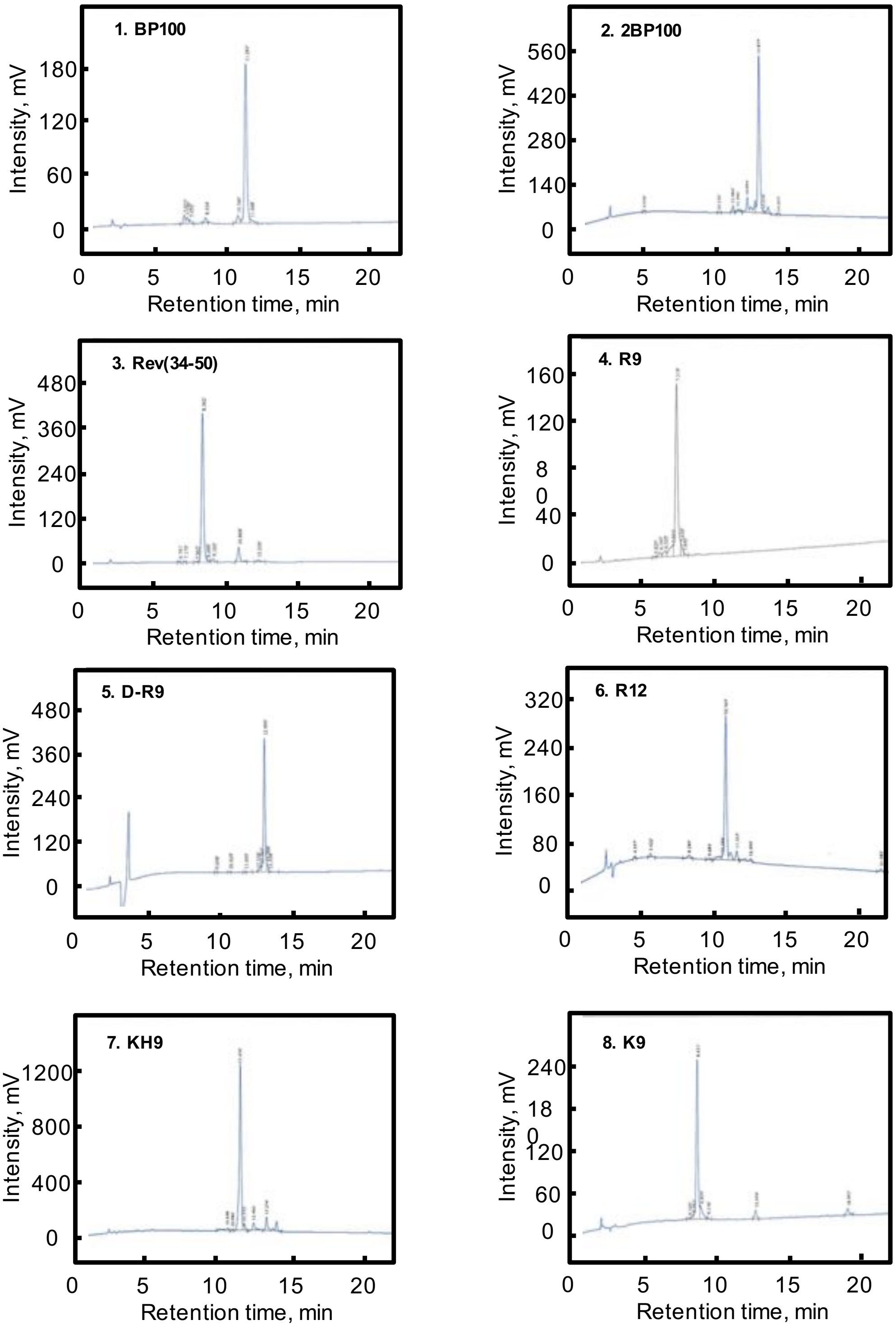
1. BP100
2. 2BP100
3. Rev(34-50)
4. R9
5. D-R9
6. R12
7. KH9
8. K9
Intensity, mV
Retention time, min

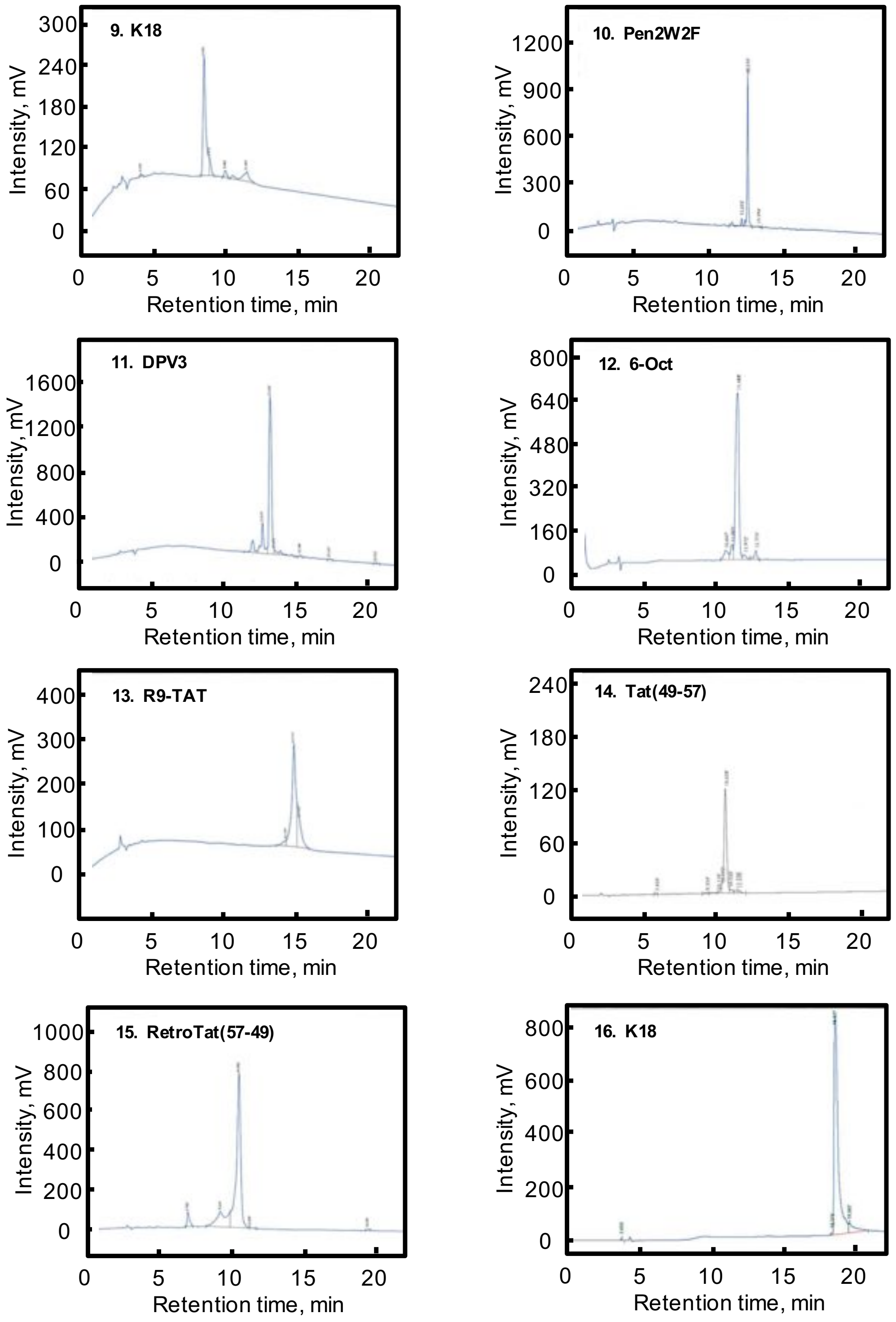
9. K18
10. Pen2W2F
11. DPV3
12. 6-Oct
13. R9-TAT
14. Tat(49-57)
15. RetroTat(57-49)
16. K18
Intensity, mV
Retention time, min

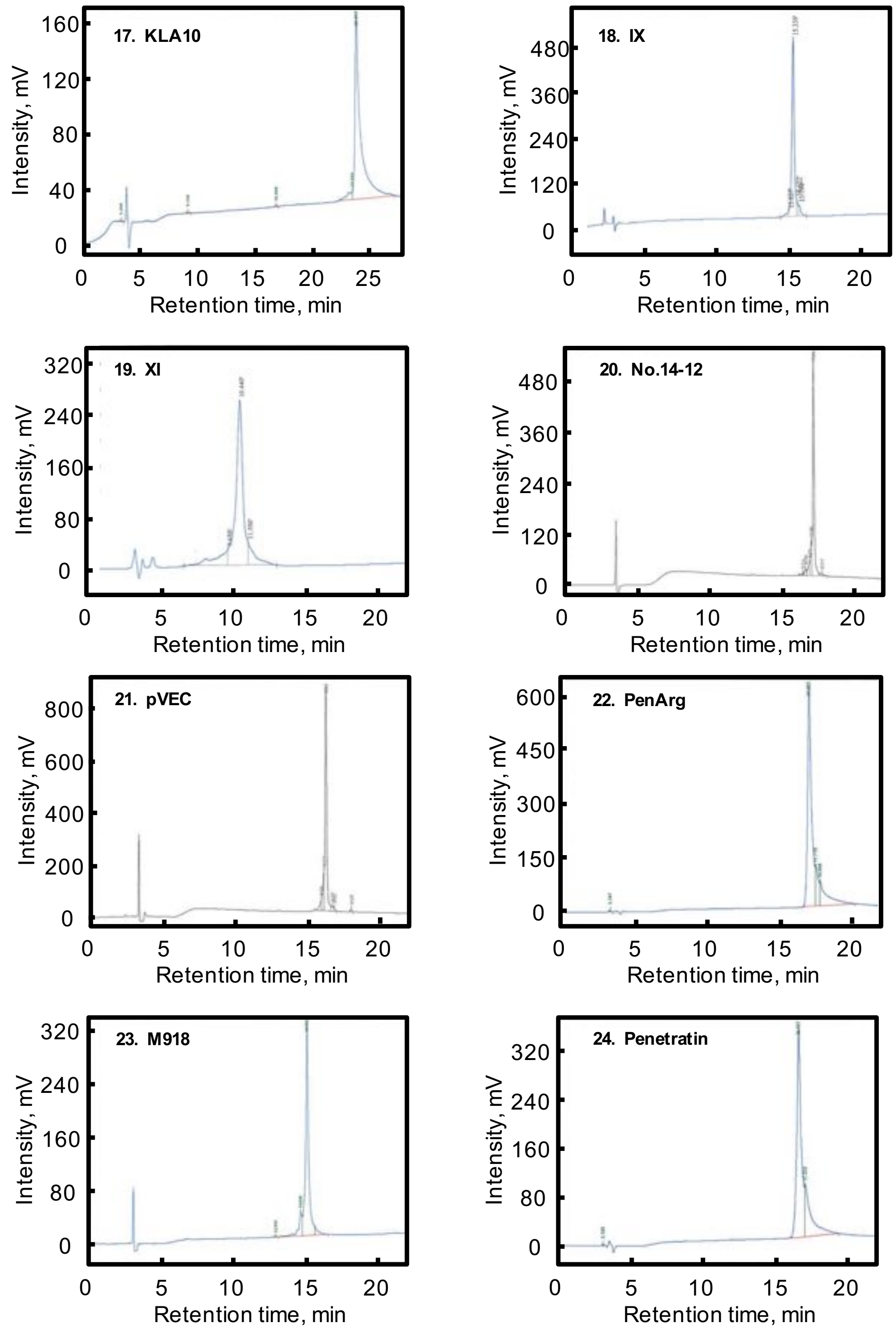
17. KLA10
18. IX
19. XI
20. No.14-12
21. pVEC
22. PenArg
23. M918
24. Penetratin
Intensity, mV
Retention time, min

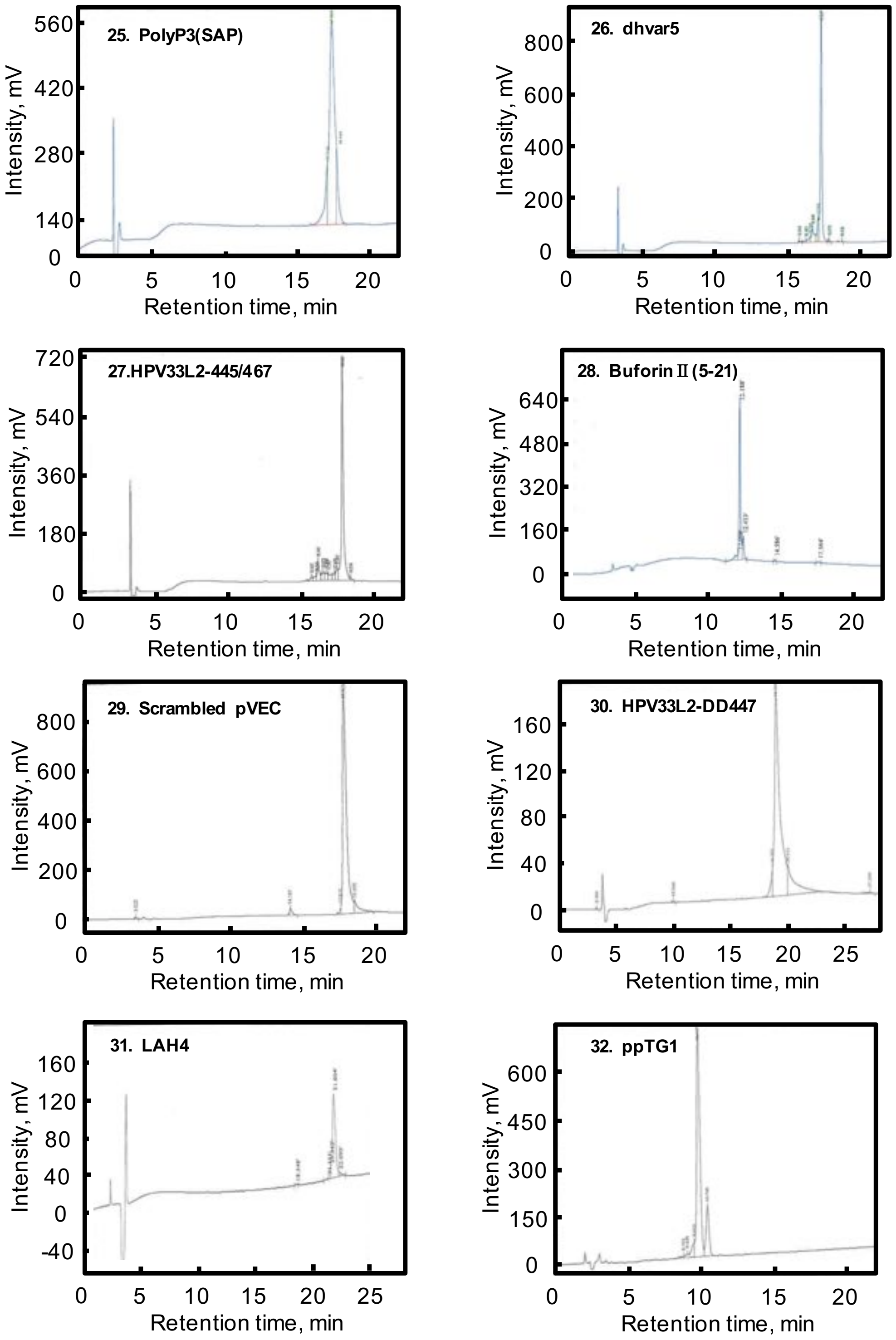
25. PolyP3(SAP)
26. dhvar5
27.HPV33L2-445/467
28. Buforin II (5-21)
29. Scrambled pVEC
30. HPV33L2-DD447
31. LAH4
32. ppTG1
Intensity, mV
Retention time, min

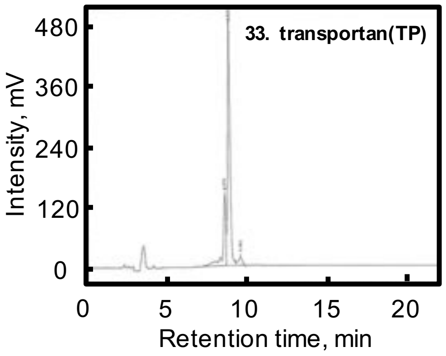
33. transportan(TP)
Intensity, mV
Retention time, min

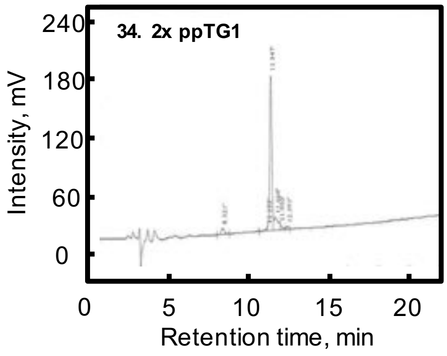
34. 2x ppTG1
Intensity, mV
Retention time, min

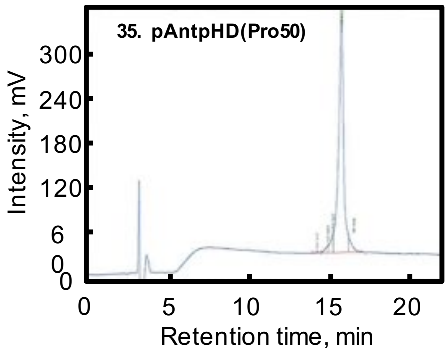
35. pAntpHD(Pro50)
Intensity, mV
Retention time, min

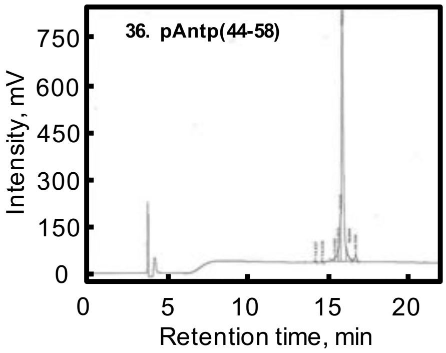
36. pAntp(44-58)
Intensity, mV
Retention time, min

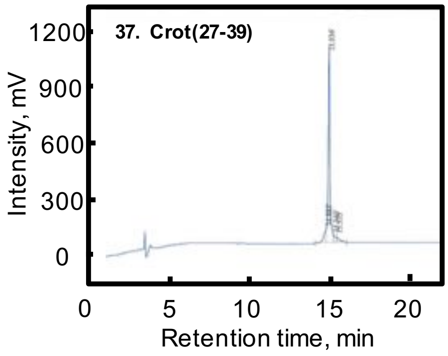
37. Crot(27-39)
Intensity, mV
Retention time, min

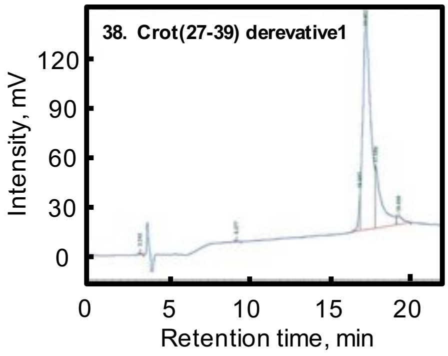
38. Crot(27-39) derevative1
Intensity, mV
Retention time, min

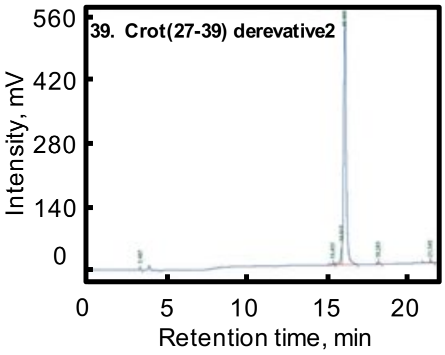
39. Crot(27-39) derevative2
Intensity, mV
Retention time, min

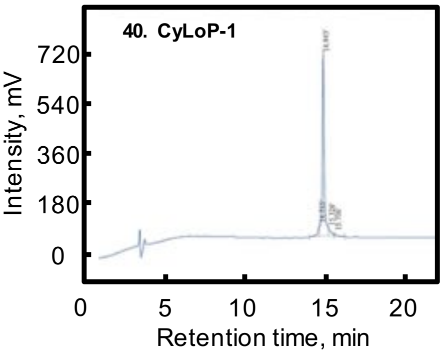
40. CyLoP-1
Intensity, mV
Retention time, min

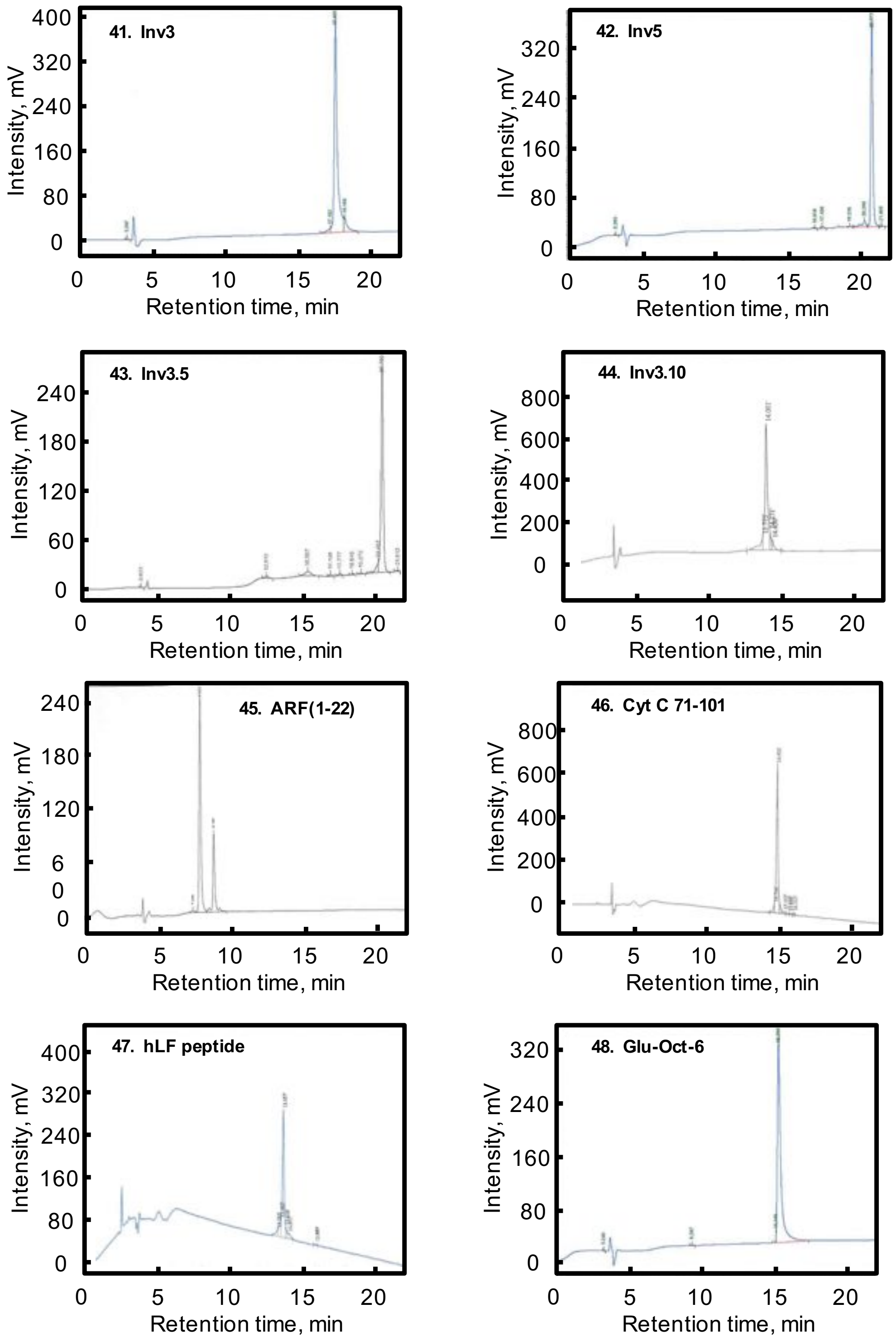
41. Inv3
42. Inv5
43. Inv3.5
44. Inv3.10
45. ARF(1-22)
46. Cyt C 71-101
47. hLF peptide
48. Glu-Oct-6
Intensity, mV
Retention time, min

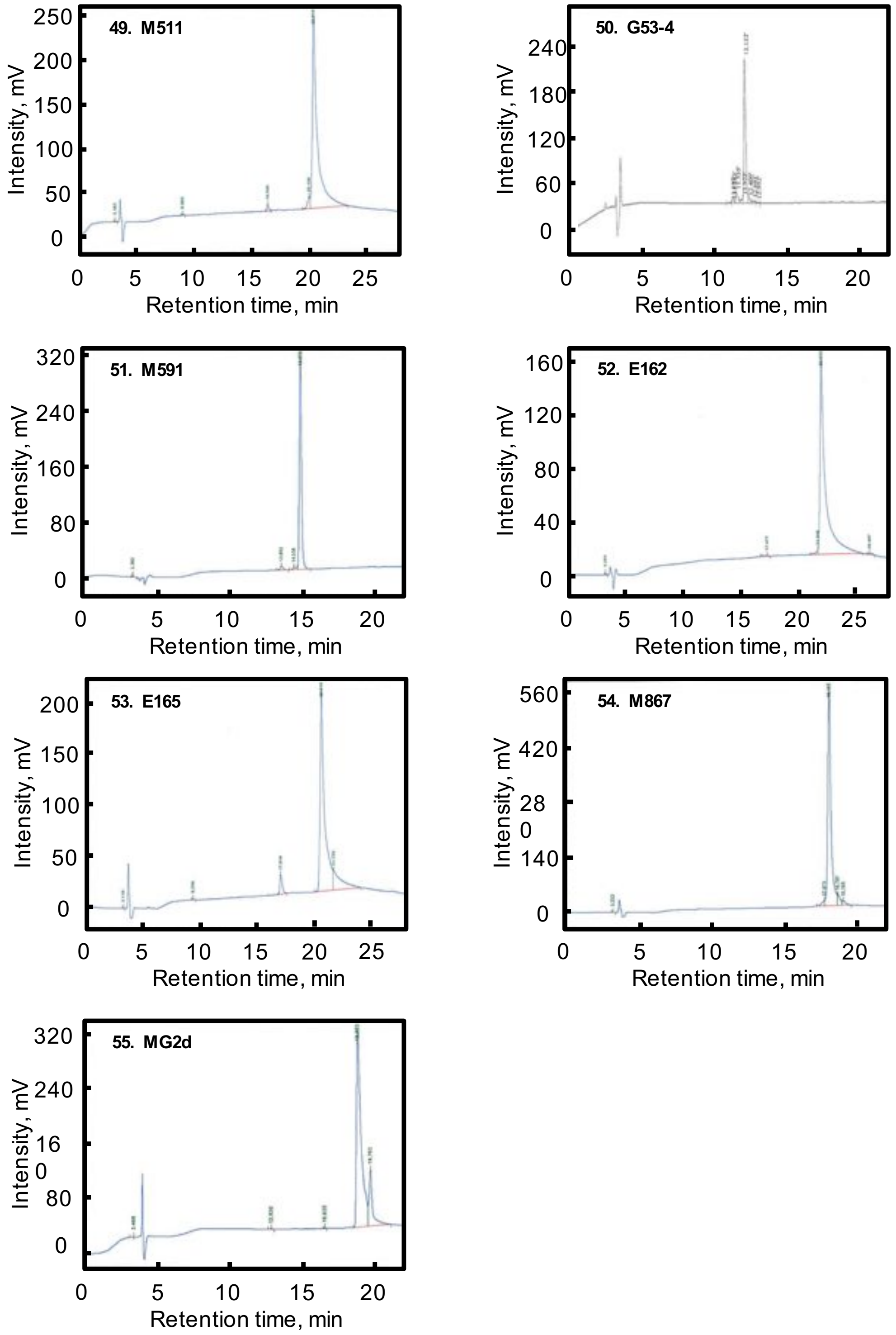

Figure S15. HPLC profiles of the peptides used in this study.

Table S1. Cell penetrating efficiency into various plant types of three groups of CPP. Based on the results with BY-2 cells, the high efficient (a), middle efficient (b) and low efficient CPPs (c) were classified and assayed with their leaves.

Peptide No.	Peptide	BY-2	*N. benthamiana*	*A. thaliana*	poplar	*S. lycopersicum*	*O. sativa*
5	D-R9	95.3±3.1	16.9±14.7	0.7±1.2	5.3±9.2	1.3±0.6	n.d.
17	KLA10	96.3±3.5	22.6±13.4	22.8±11.2	33.3±14.5	5.2±4.8	n.d.
49	M 511	90.0±4.6	27.1±10.0	21.8±8.4	30.7±18.0	3.0±1.7	n.d.
52	E162	93.3±4.5	33.3±5.9	25.5±16.1	22.0±16.4	3.7±2.7	n.d.
55	MG2d	91.3±5.5	19.3±10.5	25.4±8.2	2.7±4.6	18.0±2.2	n.d.
1	BP100	59.3±4.2	76.6±10.4	42.2±3.0	80.6±16.3	14.5±9.6	n.d.
26	dhvar5	54.0±2.6	62.6±25.3	44.3±16.1	12.6±9.9	13.4±4.5	n.d.
27	HPV33L2-445/467	51.7±2.1	47.7±7.5	70.2±7.8	27.1±12.5	65.5±19.6	n.d.
37	Crot(27-39)	51.0±4.6	50.1±13.8	10.1±1.5	21.7±12.2	21.8±9.6	n.d.
40	CyLoP-1	51.0±7.5	65.2±1.0	37.7±15.3	10.7±3.1	51.4±20.7	n.d.
11	DPV3	2.0±2.6	86.3±5.7	70.8±6.0	25.7±3.2	20.2±6.4	n.d.
14	Tat(49-57)	1.7±1.2	51.9±21.7	44.3±11.2	11.4±10.4	28.2±8.4	n.d.
15	Retro-Tat(57-49)	1.3±0.6	51.6±3.5	49.8±19.4	14.0±11.1	17.7±3.2	n.d.
34	2x ppTG1	0.3±0.6	n.d.	n.d.	n.d.	n.d.	n.d.
50	G53-4	n.d.	n.d.	3.1±3.4	21.3±12.2	n.d.	n.d.

n.d. means "not determined".
